# Investigation of alpha-glucosidase inhibition activity of *Artabotrys sumatranus* leaf extract using metabolomics, machine learning and molecular docking analysis

**DOI:** 10.1371/journal.pone.0313592

**Published:** 2025-01-03

**Authors:** Dela Rosa, Berna Elya, Muhammad Hanafi, Alfi Khatib, Eka Budiarto, Syamsu Nur, Muhammad Imam Surya

**Affiliations:** 1 Department of Pharmacy, Faculty of Pharmacy, Indonesia University, Depok, Indonesia; 2 Department of Pharmacy, Faculty of Health Science, Pelita Harapan University, Tangerang, Indonesia; 3 Chemistry Research Centre, National Research and Innovation Agency, Science and Technology Research Centre, Serpong, Indonesia; 4 Department of Pharmaceutical Chemistry, Kulliyah of Pharmacy, International Islamic University Malaysia, Kuantan, Malaysia; 5 Department of Information Technology, Faculty of Engineering and Information Technology, Swiss German University, Tangerang, Indonesia; 6 Department of Pharmaceutical Analysis and Medicinal Chemistry, Almarisah Madani University, Makasar, Indonesia; 7 Research Centre for Plant Conservation, Botanic Gardens and Forestry, National Research and Innovation Agency, Bogor, Indonesia; Monash University Malaysia, MALAYSIA

## Abstract

One way to treat diabetes mellitus type II is by using α-glucosidase inhibitor, that will slow down the postprandial glucose intake. Metabolomics analysis of *Artabotrys sumatranus* leaf extract was used in this research to predict the active compounds as α-glucosidase inhibitors from this extract. Both multivariate statistical analysis and machine learning approaches were used to improve the confidence of the predictions. After performance comparisons with other machine learning methods, random forest was chosen to make predictive model for the activity of the extract samples. Feature importance analysis (using random feature permutation and Shapley score calculation) was used to identify the predicted active compound as the important features that influenced the activity prediction of the extract samples. The combined analysis of multivariate statistical analysis and machine learning predicted 9 active compounds, where 6 of them were identified as mangiferin, neomangiferin, norisocorydine, apigenin-7-O-galactopyranoside, lirioferine, and 15,16-dihydrotanshinone I. The activities of norisocorydine, apigenin-7-O-galactopyranoside, and lirioferine as α-glucosidase inhibitors have not yet reported before. Molecular docking simulation, both to 3A4A (α-glucosidase enzyme from *Saccharomyces cerevisiae*, usually used in bioassay test) and 3TOP (a part of α-glucosidase enzyme in human gut) showed strong to very strong binding of the identified predicted active compounds to both receptors, with exception of neomangiferin which only showed strong binding to 3TOP receptor. Isolation based on bioassay guided fractionation further verified the metabolomics prediction by succeeding to isolate mangiferin from the extract, which showed strong α-glucosidase activity when subjected to bioassay test. The correlation analysis also showed a possibility of 3 groups in the predicted active compounds, which might be related to the biosynthesis pathway (need further research for verification). Another result from correlation analysis was that in general the α-glucosidase inhibition activity in the extract had strong correlation to antioxidant activity, which was also reflected in the predicted active compounds. Only one predicted compound had very low positive correlation to antioxidant activity.

## Introduction

Diabetes is a worldwide metabolic disorder disease, which affected by estimation 463 million people in 2019. The number is estimated to increase to around 578 million people in 2030 and 700 million people in 2045 [1]. This is also a killer disease, with contribution to worldwide mortality estimated to be 11.3% in 2019 [2]. Not only the number of affected people is staggering, but also the amount of financial expenditure related to this disease is impressive. It was estimated to be 760 billion US dollar in 2019, and increasing to 825 billion US dollar in 2030 and 845 billion US dollar in 2045 [3].

This disease is characterized by metabolic disorder of insulin, either in the production (secretion), functionality, or both [4, 5]. As a result of this chronical disorder, hyperglycemia, or elevated blood sugar level, is usually the characteristic symptom of diabetes. Unfortunately, the disease does not stop here. Hyperglycemia induces complications in other organs, such as obesity, hypertension, cardiovascular diseases, hypertension, kidney diseases, blindness, and even nerve damages [5, 6]. Indeed, diabetes poses major threats in fatality, if not in mortality [7].

There are several types of diabetes, but the most prevalent one is diabetes mellitus type II, which is the type in about 90% of all cases of diabetes [5, 8, 9]. Diabetes mellitus type II is caused by a combination of insulin resistance and damage in insulin secretion. This type of diabetes most often affects adults, and the risk of developing this disease is increased by hypertension, hyperlipidemia, and genetic tendency [10]. Obesity, which can be caused by unhealthy eating pattern or lack of movements, has increased the number of diabetes mellitus type II among young adults and children [10].

Unfortunately, there are still no medicine that effectively deals directly with the insulin metabolic dysfunction, which is the cause of diabetes, including diabetes mellitus type II [5]. Since hyperglycemia is the cause of many complications, many diabetes medicines are focused to control the glucose level in the blood [8, 10]. One approach is by inhibiting the α-glucosidase enzyme to decrease the glucose intake into blood from the digestion of carbohydrates in the intestine [4–6, 9]. Unfortunately, α-glucosidase inhibitors, including acarbose, usually give negative side-effects on the digestive systems, such as flatulence, diarrhea, and discomfort in abdominal area [11, 12]. Nevertheless, medicines that inhibit α-glucosidase are still used as the chosen alternatives for patients of early diabetes mellitus type II. It is also an effective medicine that can be used for patients who cannot tolerate other kind of diabetes medicines. Some of the potent α-glucosidase inhibitors, which are also approved to be used clinically, are acarbose, voglibose, and miglitol [5].

Some of the popular α-glucosidase inhibitors, such as acarbose and vogliobose, are extracted from natural products. Even metformin, one of the most popular antidiabetic medicines, is also a natural product [5]. Indeed, natural products are important sources of for drug active compounds, with more than 1/3 of active small compounds in all globally approved medicines from 1981 to 2006 came from natural origin, or semi synthetically derived from natural products [13]. These successes motivated the search of α-glucosidase inhibitors from natural products, with the hope to find α-glucosidase inhibitors with better efficacy and/or less side-effects, that ideally can also be extracted or synthesized more easily [6, 9].

*Artabotrys* is one of genus with large number of members in the family of *Annonaceae* [14]. One of the members of this genus is *Artabotrys sumatranus* which is a climbing plant that can be found on the islands of Sumatera, Java, and Borneo in Indonesia [15]. Not many research has been done to investigate the active compounds in this plant, but a recent study [16] has found out that the leaf of this plant has potential to inhibit α-glucosidase, and moreover it was predicted that the α-glucosidase active compound in the leaf of this plant had also antioxidant activity. These double activities are beneficial since hyperglycemia in diabetes mellitus type II patients can induce oxidative stress, which can result in cell damage [17]. To help avoiding the cell damage due this oxidative stress, antioxidants are indeed needed [18].

Since *Artabotrys sumatranus* leaf extract has been found to show α-glucosidase inhibition and antioxidant activities, it is of interest to find the active compounds in the extract. One of the new approaches for finding active compounds from natural products, including plants, is metabolomics, especially untargeted metabolomics. Metabolomics is a comprehensive study that analyses and evaluates the data of the small molecules (usually called metabolites) in cells, tissues, and body fluids [19]. It is called untargeted metabolomics if the active compounds are not yet known. Untargeted metabolomics can be used to predict the active compounds from natural products. Metabolomics analysis can help to find the profile pattern of compounds which corresponds to the activity of the extract, which can be the result of one active compound or the synergy of several compounds [20]. In metabolomics, the prediction of the active compound is made by finding the pattern in the data of the existing compounds in the extracts (usually obtained from LC/MS (liquid chromatography mass spectrometry) or other spectrometry analysis such as GC/MS (gas chromatography mass spectrometry) and NMR (nuclear magnetic resonance)), coupled with the activity data of the extracts obtained from bioassay analysis [21]. In metabolomics, the data can be analyzed using multivariate statistical analysis [22–27] such as PCA (principal component analysis), PLS (partial least squares), OPLS (orthogonal projection to latent structures), PLS-DA (partial least squares–discriminant analysis), and OPLS-DA (orthogonal projection to latent structures–discriminant analysis). Besides the multivariate statistical analysis, machine learning such as random forest can also be used in metabolomics analysis [28–30]. The multivariate statistical analysis has been used in natural product–drug discovery, such as to predict the α-glucosidase inhibitors from *Clinacanthus nutans* leaf, *Psychotria malayana*, and *Capsicum spp* [31–33], and also to discover the synergy between berberine and piperine as the antimicrobial against *Staphylococcus aureus* [34]. Meanwhile, machine learning techniques have also been used in different stages at drug discovery [30]. For example, the bioactivity of abaucin, an antibiotic to cure infection caused by *Acinetobacter baumannii*, was identified using machine learning [35]. Moreover, halicin, an antimicrobial agent that has a wide phylogenetic spectrum of pathogens, was also found using the deep learning and artificial neural network, which are some examples of machine learning methods [36].

Beside doing the isolation to get the predicted active compounds, verification of the metabolomics analysis can also be done by doing in-silico analysis, namely by using molecular docking technique [37, 38]. In this technique, the binding (or docking) of the predicted active compound to the protein receptor is simulated in computers. The strength of the docking of the compound to the binding site will be computed as the free binding energy. The more negative the free binding energy, the stronger the binding is. By analyzing the free binding energy, the activity of the compound can be deduced. This technique has been used in drug discovery [37, 39], for example to find α-glucosidase inhibitors from marine brown alga *Dictyopteris hoytii* [40], Chinese bayberry (*Morella rubra* Sieb. et Zucc.) fruit [41], *Artabotrys hexapetalus* stembark and leaf [42], and *Artabotrys suaveolens* stembark and leaf extracts [43]; as well as potential main protease inhibitors of COVID-19 or SARS-Cov-2 virus [44, 45].

This research’s goal was to find the active compound(s) in the leaf of *Artabotrys sumatranus* that showed high potential in α-glucosidase inhibition activity, and ideally also in antioxidant activity. The activity screening of this plant’s leaf ethanol extract showed promising potential of these two activities [16]. One of the uniqueness of this research, besides being the first research to find α-glucosidase inhibitor(s) and antioxidant(s) in *Artabotrys sumatranus* extract, was the use of two metabolomics approaches: multivariate statistical analysis and machine learning. The goal was not to compare the results of these two approaches, but to combine the results to get predictions of active compounds with higher confidence level. The predictions of metabolomics analysis were verified using molecular docking and isolation using bioassay guided fractionation.

## Materials and methods

### Overview of the methods

This research analyzed the LC-MS results of the *Artabotrys sumatranus* leaf ethanol extracts (with different proportion of ethanol dan water) with metabolomics techniques. Two kinds of metabolomic techniques were used: multivariate statistical methods and machine learning methods. In this research, after trying different methods, the chosen machine learning method was random forest [46]. The metabolomic analysis with machine learning was done in 2 stages: the first stage was the development of predictive model for the activity of the extracts with different proportions ethanol and water, and the second stage was the prediction of the active compounds as the most important features (variables) for the predictive extract activity model. For the machine learning model, the feature importance was determined using random permutations [47–49] and SHAP (SHapley Additive exPlanations) method [50, 51]. In multivariate statistical methods, the first and second stages were combined directly, and therefore the identification of the predicted active compounds did not need other methods.

The identifications of the predicted active compounds were done by analyzing the LC-MS/MS spectrometry results of the extract. The further fragmentations of m/z values of the predicted active compounds were compared to the database of the LC-MS/MS machine. The fragmentation pattern results were also analyzed to ascertain the feasibility of the fragmentations for the identified active compounds.

In the end, the prediction of the metabolomic analysis was verified by molecular docking analysis and isolation of the active compound using bioassay guided fractionation [52, 53]. Bioassay guided fractionation, instead of metabolomics guided fractionation [54, 55], was used in this research due to limitation of the quantity of extracts available. Metabolomics guided fractionation can actually be used to isolate the not-yet-identified active compounds, but it requires more quantity of extracts due to repeated LC-MS analyses for every fraction.

### Materials

The materials used in this research were as follows: α-glucosidase from *Saccharomyces cerevisiae* (EC 232-604-7), p-nitrophenyl α-d-glucopyranoside (PNPG), acarbose (Sigma Aldrich, USA), dimethyl sulfoxide (DMSO), dipotassium hydrogen phosphate, potassium dihydrogen phosphate monohydrate, sodium carbonate, bovine serum albumin, TLC aluminum silica gel 60F_254_, Sephadex LH-20, chloroform (Merck, Germany), ethanol, n-hexane, ethyl acetate, 1,1-diphenyl-2-picrylhydrazyl (DPPH) (Smart Lab, Indonesia), and ascorbic acid (Loba Chemie, India).

The equipment used were freeze dryer (Alpha 1–2 LDplus Martin Christ, Germany), rotary evaporator (Heidolph Hei-Vap Core, Schwabach, Germany), vortex (Reax Top Heidolph, Schwabach, Germany), incubator (Memmert IN55, Schwabach, Germany), UV spectrophotometer (Thermo Scientific Orion Aquamate 8000), microplate reader (HiPo MPP-96 Biosan), LC-MS (Agilent 6520 Q-TOF LC/MS System with Agilent 1200 Series HPLC), LC-MS/MS (Waters Vion IMS QTOF with acuity UPLC, USA), Ascend FTNMR (Bruker 700MHz), computer (Lenovo Legion 7, China) with processor AMD Reizen 7 5800H and RAM 32 GB. The software used were AutoDock Tools 1.5.7, AutoDock FR (ADFR) suite 1.0 (especially “prepare ligand” module to prepare the ligands in batches), ChimeraX 1.7, Open Babel 3.1.1, Autodock Vina version 1.2.3, Biovia Discovery Studio 21, SIMCA-P+ 14, and MZMine 2.53.

### Plant material preparation

*Artabotrys sumatranus* leaf (collection number VI.D.148, access number C2009090117) was harvested from Cibodas Botanical Garden in West Java, Indonesia, which was originally collected by Iyung and Wiguna Rahman from the National Park Mount Leuser (latitude 03° 50′ 02.9″ N and longitude 97° 31′ 17.2″ E) in Aceh, Sumatra, Indonesia. 700 g *Artabotrys sumatranus* leaf were washed and dried using a freeze dryer, then continued by grinding, resulting in 350 g *Artabotrys sumatranus* leaf powder. Each 4 g of leaf powder was macerated with 20 ml of ethanol for 24 hours and then filtered. The maceration process was repeated twice. The filtrate was collected and dried by putting it in an oven whose temperature was set to be 40°C for one week. The extraction process was done for 5 different mixtures of ethanol and water as solvents: 100% ethanol (0% water), 75% ethanol, 50% ethanol, 25% ethanol, and 100% water (0% ethanol). Extract with each solvent mixture was repeated 6 times, resulting in total of 30 samples. Each sample was tested using α-glucosidase inhibition and DPPH radical scavenging (for antioxidant) activity assays. The values of IC_50_ of both α-glucosidase inhibition and DPPH radical scavenging activities for all the extract samples were shown in S1 Table.

### α-glucosidase inhibition assay

This assay was done according to Elya et al. [56]. First, the 36 μl phosphate buffer solution with pH 7.0 was added by 17 μl p-nitrophenyl-α-d-glucopyranoside (PNPG) 5mM. Second, 30 μl samples with varying concentrations in DMSO (dimethyl sulfoxide) were added, homogenized and incubated at 37°C for 5 minutes. Third, the mixture was added with 17 μl α-glucosidase (0.062 unit) and the solution was incubated at 37°C for 15 minutes. Lastly, 0.1 ml sodium carbonate (Na₂CO₃) 0.2 M was added to stop the reaction, and the absorbance was measured at 405 nm using microplate reader. The control solution was a PNPG, α-glucosidase, and phosphate buffer mixture, while the positive control was acarbose. The percentage of α-glucosidase inhibition activity was calculated using the following formula:

$$\%Inhibition=\frac{Abs\;control–Abs\;sample}{Abs\;control}\times 100\%$$ (1)

This assay was conducted with several concentrations in triplicate to get the IC_50_ value.

### DPPH inhibition assay

DPPH (2,2-diphenyl-1-picrylhydrazyl) assay was used to measure the antioxidant activity. This method was done based on the methods used by González-Palma et al. [57] and Rosa et al. [16]. The mixture of 1 ml of DPPH 0.175 mM and 0.8 ml sample in the ethanol was incubated in dark condition for 30 minutes and the absorbance was measured at 517 nm using UV spectrophotometer (Thermo Scientific Orion Aquamate 8000). The control was DPPH and ethanol while ascorbic acid was used as positive control. The blank used was ethanol. This assay was done triplicate for each sample. Antioxidant activity was expressed in percentage of DPPH reduction by the following calculation:

$$\%reduction\;DPPH=\frac{Abs\;control–Abs\;sample}{Abs\;control}\times 100\%$$ (2)

This assay was conducted with several concentrations in triplicate to get the IC_50_ value.

### LC-MS-QTOF instrument and preparation

The LC/MS-QTOF system used in this study comprised of an Agilent 1200 liquid chromatography system, equipped with a binary pump, a vacuum degasser unit, an auto sampler and 6520 quadrupole time of flight mass spectrometers with an electrospray ionization (ESI) source. Column used was Agilent ZORBAX Eclipse Plus C_18_ Rapid Resolution HT (2.1 x 100 mm) 1.8 μm.

The preparation of the sample for the LC MS–QTOF analysis was done according to Murugesu et al. [31]. 250 μl of methanol was added to 1 mg sample of plant extract, then it was homogenized for 15 minutes. Afterward, 250 μl water was added and centrifuged for 15 minutes and the supernatant was put into a glass vial through syringe filtration to make it ready for injection to the machine. The chromatographic separation was done at 40°C using Agilent ZORBAX Eclipse Plus C_18_ Rapid Resolution HT (2.1 x 100 mm) 1.8 μm with 0.1% formic acid in dH_2_0 as mobile phase A and 0.1% formic acid in acetonitrile as mobile phase B. The separation was done in positive mode. The gradient elution was set 5–95% mobile phase B at 0.00–18.00 minutes then continued with 95% mobile phase B until minutes 23 and at 23.01 minutes the mobile phase B was set to be 5%. The total run time is 30 minutes. The LC condition was re-equilibrated for 2 minutes before starting the new injection. The sample injection volume was set at 2 μl and the flow rate of the mobile phase was set at 0.25 ml/min. Meanwhile, the mass spectrometer was operated in positive electrospray ionization (ESI) mode with optimum gas temperature at 325°C, gas flow at 11 L/min, and nebulizer at 35 psi, respectively.

### LC-MS/MS QTOF instrument and preparation

LC-MS/MS QTOF was used to get further fragmentation patterns for the m/z values of the predicted active compounds. These further fragmentation patterns were used to identify the active compounds, which was done by comparing the fragmentation pattern with the available database in the LC-MS/MS QTOF machine.

For LC-MS/MS analysis in this study, UHPLC was used. The sample for the LC-MS/MS analysis was prepared by mixing 55 mg extract sample with 2 ml ethanol. Here only ethanol was used since all the predicted active compounds were from extracts that use 100% ethanol as solvent. The mixture was filtered, and then 2 μl of the filtration result was injected into the UHPLC machine. UHPLC was performed on ACQUITY UPLC I-Class system from Waters, consisting of a binary pump, a vacuum degasser, an auto- sampler and a column oven. The compounds were chromatographically separated using a column ACQUITY UPLC HSS T3 (100 mm x 2.1 mm x 1.8 μm) also from Waters, maintained at 40°C. A linear binary gradient of water (0.1% formic acid) and acetonitrile (mobile phase B) was used as mobile phase A and B respectively. The mobile phase composition was changed during the run as follows: 0 min, 1% B; 0.5 min, 1% B; 16.00 min, 35% B; 18.00 min, 100% B; 20.00 min, 1% B. The flow rate was set to 0.6 mL/min and the injection volume was 2 μL.

The UHPLC system was coupled to a Vion IMS QTOF hybrid mass spectrometer from Waters, equipped with a Lock Spray ion source. The ion source was operated in positive electrospray ionization (ESI) mode under the following specific conditions: capillary voltage, 1.50 kV; reference capillary voltage, 3.00 kV; source temperature, 120°C; desolvation gas temperature, 550°C; desolvation gas flow, 800 L/h, and cone gas flow, 50 L/h. Nitrogen (>99.5%) was employed as desolvation and cone gas. Data were acquired in high-definition MS^E^ (HDMS^E^) mode in the range m/z 50–1500 at 0.1 s/scan. Thus, two independent scans with different collision energies (CE) were alternatively acquired during the run: a low-energy (LE) scan at a fixed CE of 4 eV, and a high- energy (HE) scan where the CE was ramped from 10 to 40 eV. Argon (99.999%) was used as collision-induced-dissociation (CID) gas.

### LC-MS and LC-MS/MS data processing

The raw data from LC-MS was converted to MZML format using MS Convert (http://proteowizard.sourceforge.net/tools.shtml). After that, the data was processed using MZmine 2.5.3 [58] such as the baseline correction (m/z bin width is 1.000, smoothing is 100.000, and asymmetry is 0.5), mass detection (noise detection is 0), ADAP chromatogram (group intensity threshold and minimum highest intensity is 2.5E3, m/z tolerance is 0–10 ppm, chromatogram deconvolution (minimum peak height is 2.5E3 and peak duration range 0–10 minutes), isotope grouping (m/z tolerance 10 ppm, retention time tolerance is 0.1), join aligner (weight for m/z is 75 and weight for retention time is 25), and gap filling (intensity tolerance is 10%).

The fragmentation patterns to validate the identification of the predicted active compounds were obtained from LC-MS/MS machine directly. For each m/z of the predicted active compound, a further fragmentation pattern was acquired. No data preprocessing was needed since the fragmentation patterns were already in good form.

### Metabolomics analysis

The processed LC-MS data of the 30 extract samples were used as the input of the metabolomics analysis. The features (variables) were the detected m/z values in all extract samples. There were 80 features in total, and the values of these features were the amounts of the compounds corresponding to the m/z values in the extract samples (the values could be zero if the compounds were not detected in the extract). The list of the features can be seen in S2 Table. This input data was added with the activity data, which were the IC_50_ of α-glucosidase inhibition (AGI) and IC_50_ of DPPH assay (for antioxidant activity). To make the values of the activity data to be increasing when the activity is stronger, the values of 1/IC_50_ of α-glucosidase inhibition (AGI) and 1/IC_50_ of DPPH assay were used. The activities were used as the outputs (targets) in the data analysis. To remove the dominance of large-valued features, the input data was normalized to have zero mean and unity variance (UV) for each feature.

The input data was then analyzed using untargeted metabolomic methods. The analysis was done by two kinds of methods. The first one was multivariate statistical analysis: PCA (principal component analysis), PLS (partial least squares), OPLS (orthogonal projection to latent structures), PLS-DA (partial least squares–discriminant analysis), and OPLS-DA (orthogonal projection to latent structures–discriminant analysis). All these methods tried to find new projection axis (coordinate systems) which can separate the data best, according to their own criteria [22–27]. For simplicity, these projection axes were called principal components in this research. The principal components were weighted linear combinations of the original features (m/z values of the detected compounds in the extract samples). These weights were called loadings of the principal components. By analyzing the loadings, the most important features that contributes to the activities of the extracts were found. The analysis of the loadings was done by inspecting the values of the loadings, and also checking the VIP (variable importance for projection) [24]. The VIP value must be more than 1 to make the feature (input variable) to be important.

For the PLS-DA and OPLS-DA, classes were needed, and it was set up as active class (all 100% ethanol extracts) and non-active class (the rest of the data). This division of the class was based on the observation that all 100% ethanol extracts had higher 1/IC_50_ of α-glucosidase inhibition (AGI) values than the rest.

Combinations of the methods and the outputs created 25 models which were investigated. The models (M1 –M25) can be seen in Table 1.

**Table 1:** Statistical multivariate models for leaf extract of *Artabotrys sumatranus*, with their analytical methods, input variables (X) and output variabel (Y).

Model	Methods	Input variable X	Output variable Y
M1	PCA	m/z	None
M2	PLS	m/z	1/IC_50_ AGI
M3	PLS	m/z	IC_50_ AGI
M4	PLS–DA	m/z	1/IC_50_ AGI
M5	OPLS -DA	m/z	1/IC_50_ AGI
M6	OPLS	m/z	1/IC_50_ AGI
M7	PLS	m/z	1/IC_50_ AGI and 1/IC_50_ DPPH
M8	OPLS	m/z	1/IC_50_ AGI and 1/IC_50_ DPPH
M9	PLS–DA	m/z	IC_50_ AGI
M10	OPLS–DA	m/z	IC_50_ AGI
M11	OPLS	m/z	IC_50_ AGI
M12	PLS	m/z	IC_50_ AGI and IC_50_ DPPH
M13	OPLS	m/z	IC_50_ AGI and IC_50_ DPPH
M14	PLS–DA	m/z	1/IC_50_ AGI and 1/IC_50_ DPPH
M15	OPLS–DA	m/z	1/IC_50_ AGI and 1/IC_50_ DPPH
M16	PLS–DA	m/z	IC_50_ AGI and IC_50_ DPPH
M17	OPLS–DA	m/z	IC_50_ AGI and IC_50_ DPPH
M18	PLS	m/z	1/IC_50_ DPPH
M19	PLS–DA	m/z	1/IC_50_ DPPH
M20	OPLS	m/z	1/IC_50_ DPPH
M21	OPLS–DA	m/z	1/IC_50_ DPPH
M22	PLS	m/z	IC_50_ DPPH
M23	PLS–DA	m/z	IC_50_ DPPH
M24	OPLS	m/z	IC_50_ DPPH
M25	OPLS–DA	m/z	IC_50_ DPPH

The validity of these 25 models were first tested based on the following criteria [59–62]:

- Differences between goodness-of-fit coefficients (R^2^) for the regression of the output variables of the original data (R^2^_Y_) and output prediction of the cross-validated data (Q^2^) should not be more than 0.3. R^2^_Y_ measures how good the model fits to the training dataset, while Q^2^ to the test dataset [27]. If the differences between these two coefficients are large, then there is an indication of overfitting (the model fits too much to the training dataset and loses some capabilities to predict general data).
- In the permutation results, where the values of output Y were permuted randomly with different correlation coefficients to the original Y, while keeping the values of the input X, the decrease in the values of R^2^_Y_ and Q^2^ of the permuted data must be sufficiently lower than the original (unpermuted) ones. If the values from the permuted simulations are significantly low, then it indicates that the unpermuted model fits are good and valid. This is measured from the regression plot of the values permuted R^2^_Y_ and Q^2^ with respect to the correlation coefficients of the permutation to the original Y: the intercept of regression line for the permuted R^2^_Y_ ≤ 0.4 and the intercept of regression line for the permuted Q^2^ ≤ 0.05. The number of permutations for this research was taken to be 20.
- The p-value of the cross-validation ANOVA (CV-ANOVA) ≤ 0.05. Cross validation ANOVA [63] tests whether the cross-validated output prediction is significantly different to the just the mean of the outputs, and it can only be used for methods based on PLS and OPLS.

After applying the validity conditions, 11 models were found to be valid. The valid multivariate statistical models can be seen in Table 2, where the values of the validity conditions above are also shown. For each of these 11 models, the most important features were selected by analyzing the respective loadings of the principal components. In total 25 most important features were selected from each model in Table 2. Then comparisons were made to determine the features that most frequently appeared in these 11 models. These most frequently appeared features were taken as the prediction of the active compounds using multivariate statistical methods. All these results were computed using software SIMCA-P+ 14.

**Table 2:** Valid multivariate statistical models for *Artabotrys sumatranus* leaf extracts, with the values of the validity conditions, divided for different outputs if the models have several outputs.

No.	Model	Intercept of permuted R^2^	Intercept of permuted Q^2^	R^2^_Y_ –Q^2^	p-value of CV-ANOVA	Output variable
1	M2	0.239	-0.224	0.160	0.00000799	
2	M3	0.370	-0.267	0.188	0.00149557	
3	M6	0.310	-0.550	0.132	0.00000895	
4	M7	0.313	-0.218	0.127	0.00005730	1/IC_50_ AGI
					0.00028243	1/IC_50_ DPPH
5	M8	0.261	-0.478	0.178	0.00142708	1/IC_50_ AGI
					0.00210300	1/IC_50_ DPPH
6	M11	0.375	-0.538	0.220	0.00312608	
7	M12	0.343	-0.283	0.126	0.00480171	IC_50_ AGI
					0.00000992	IC_50_ DPPH
8	M13	0.234	-0.218	0.180	0.00785429	IC_50_ AGI
					0.00002841	IC_50_ DPPH
9	M18	0.374	-0.345	0.100	0.00004086	
10	M20	0.387	-0.577	0.142	0.00010182	
11	M22	0.238	-0.185	0.147	0.00000079	

The second kind of methods which was used to do metabolomics analysis in this research was machine learning. Several machine learning methods were tried: decision tree [64], random forest [46], gradient boosting [65], artificial neural network [66], and ADA boost [67]. The calculations for the machine learning methods were done using Orange Data Mining software version 3.34 (https://orangedatamining.com/). The parameters used for the methods followed the defaults of the Orange Data Mining software, with some changes. For the decision tree, the changes were that only binary trees were used, the minimum number of samples on the leaf node was set to 2, subsets with samples less than 5 were not divided anymore, and target purity of the node was set to 95%. The division of the data for the decision tree method would be stopped if one of these conditions were reached. For random forest method, the changed parameters were that 100 decision trees were used in the method, and subsets with samples less than 2 were not divided anymore in the decision trees of the random forest. Gradient boosting implementation used 100 decision trees, with the maximum depth level of the decision trees was set to 3, subsets with samples less than 2 would not be divided anymore in the decision trees of gradient boosting, and learning rate was set to 0.05. The artificial neural network was implemented with 4 hidden layers with 100 nodes each, activation function in each node was ReLu, the weight optimization was done using Adam solver with 0.006 as regularization coefficient, and maximum iteration was set to 500. In the ADA boost method, the base estimator was set to be 100 decision tree stumps, using SAMME.R algorithm with linear loss function and learning rate for the weight adaptation = 0.01.

First the machine learning methods were used to make a prediction model for the activity of the extract samples. The target of prediction was 1/IC_50_ AGI (α-glucosidase inhibition). The input data was the same as the multivariate statistical methods. This input data was then divided using random sampling into training set (50%) and test set (50%). The training set was used to build the models, while the testing was done on the test set. The performance of the models was measured mainly by the difference of the predicted and measured (true) values of 1/IC_50_ AGI in the form of RMSE (root mean square error). The smaller the value of RMSE, the better the performance was.

Comparisons of the performances of the machine learning methods were done to determine the best machine learning method which would be investigated further. Here beside RMSE, the performance was also measured by the value of R^2^ (goodness-of-fit) of the regression line between the predicted value of the output (1/IC_50_ AGI) with the its true value from measurement. The bigger the value of R^2^ (the closer it was to 1), the better the performance was. The performance was evaluated for both the training and test sets.

The prediction of the activity of extract samples was not the end goal of the machine learning analysis. The goal was to find the active compounds in the extract samples, which translated into finding the most important features (m/z values of the compounds in the extracts) which had the most influence on the activity prediction of the extract samples. This untargeted metabolomics analysis (untargeted since the active compounds were not known) was done only for the machine learning method with the best performance in predicting the activity of the extract samples. Several methods were used to find the active compounds. First, before the predictive models were computed, the correlations were calculated between the values of m/z responses (indicating how much the corresponding compounds were found in the extract sample) and the target 1/IC_50_ AGI (indicating the strength of α-glucosidase inhibition activity). The features (m/z values) that had the strong positive correlation with the target were candidates for active compounds. Correlation analysis was also done between the features and 1/IC_50_ DPPH (indicating the strength of antioxidant activity), because the side goal of this research was to find an α-glucosidase inhibitor which was also an antioxidant.

Second, after the predictive models were developed, the most important features were computed using 2 methods: random permutations [47–49] and SHAP (SHapley Additive exPlanations) method [50, 51]. In random permutation, values of the investigated feature were randomly permuted. In this research the number of permutations was set to 100. If the investigated feature was important (had strong influence on the extract activity prediction), then the performance of the extract activity prediction would decrease strongly when random permutation was done to the values of this feature. The most important features could be found by identifying the features with the highest increase of RMSE (error) after random permutation. SHAP method computed a score (called the Shapley score) which corresponded to the contribution of the investigated feature to the prediction output (in this research the value of 1/IC_50_ AGI). The Shapley score of a certain feature for a certain model was computed for every extract sample by comparing the prediction output of the model using sets of features that contain the investigated feature, with prediction output of the same model but now using sets of features that do not contain the investigated feature. The Shapley score could be positive (the investigated feature would increase the value of the prediction output), zero (no contribution of the investigated feature to the prediction output), and negative (the investigated feature would decrease the value of the prediction output). Both positive and negative Shapley scores were taken to be important, and therefore the reported SHAP score for a certain feature was computed as the mean of the absolute values of the Shapley scores for each extract samples for that particular feature.

In this research, 25 features that showed the most dominant influence (the most important features) were taken from each analysis: random permutation, SHAP, and correlation analysis. The list of each analysis would be compared, the features that showed most frequently in all the lists would be taken as the predicted active compounds from machine learning approach.

The predicted active compounds from the multivariate statistical analysis would be compared to the results of machine learning approach. The features that appeared in both approaches would be taken as the predicted active compounds of the metabolomics analysis.

### Molecular docking

Molecular docking is a computational method that investigates how good a relatively small compound (called ligand) can bind (or dock) in a protein or receptor. In general, the method will try to move the ligand as a whole, or rotate a part of it (depending on how flexible the ligand is), to fit the ligand into the binding (or docking) site of the receptor. The fitting process is done by minimizing a cost function, that usually resembles the free binding energy [68].

In this research, the ligand was the predicted active compound, while the receptor was the α-glucosidase enzyme. The structure coded as 3A4A from Protein Data Bank or PDB (https://www.rcsb.org/) was usually taken as the receptor. This structure is actually the homology of α-glucosidase enzyme from *Saccharomyces cerevisiae*, which is usually used in the bioassay of α-glucosidase inhibition [69]. To simulate the binding in human, this research used the structure 3TOP from PDB, which resembles a part of the α-glucosidase enzyme in human gut [70]. The structures of the ligand, obtained from online databases such as ZINC (https://zinc.docking.org/) or PubChem (https://pubchem.ncbi.nlm.nih.gov/), was transformed into PDB or PDBQT format using OpenBabel tool (http://openbabel.org/index.html). Both receptor dan ligand files must be first prepared for simulation, for example to remove water molecules, addition of charges, choosing the grid box for the molecular docking simulation, etc., which were done by using AutoDock Tools 1.5.7 (https://autodocksuite.scripps.edu/adt/) for the receptor files, and “prepare ligand” module in AutoDockFR (https://ccsb.scripps.edu/adfr/) for batch processing of the ligand files. Sometimes the protein or receptor file must be first repaired, e.g. for missing atoms, which was done using Swiss Model tool (https://swissmodel.expasy.org/) and ChimeraX software [71] for 3A4A and 3TOP structures respectively. The molecular docking simulation itself was done using AutoDock Vina 1.2.3 [72]. The parameters of AutoDock Vina must be tuned to get the best docking result. The simulation was done on a computer with the following specifications: processor AMD Ryzen 7 5800H and RAM 32 GB. To present the result of molecular docking visually, including showing the types of binding that occurred, Biovia Development Studio software (https://www.3ds.com/products/biovia) was used.

The validation of the molecular docking results was done by comparing the position and orientation of molecular docking result and the actual binding of the native ligand, which was obtained from X-ray diffraction analysis. The error of this comparison was represented in the form of RMSD (root mean square deviation) [73]. To make the choice of the docking parameters valid, the RMSD of the native ligand was required to be ≤ 2 Å (see for example [16, 42]).

**Fig 1:**
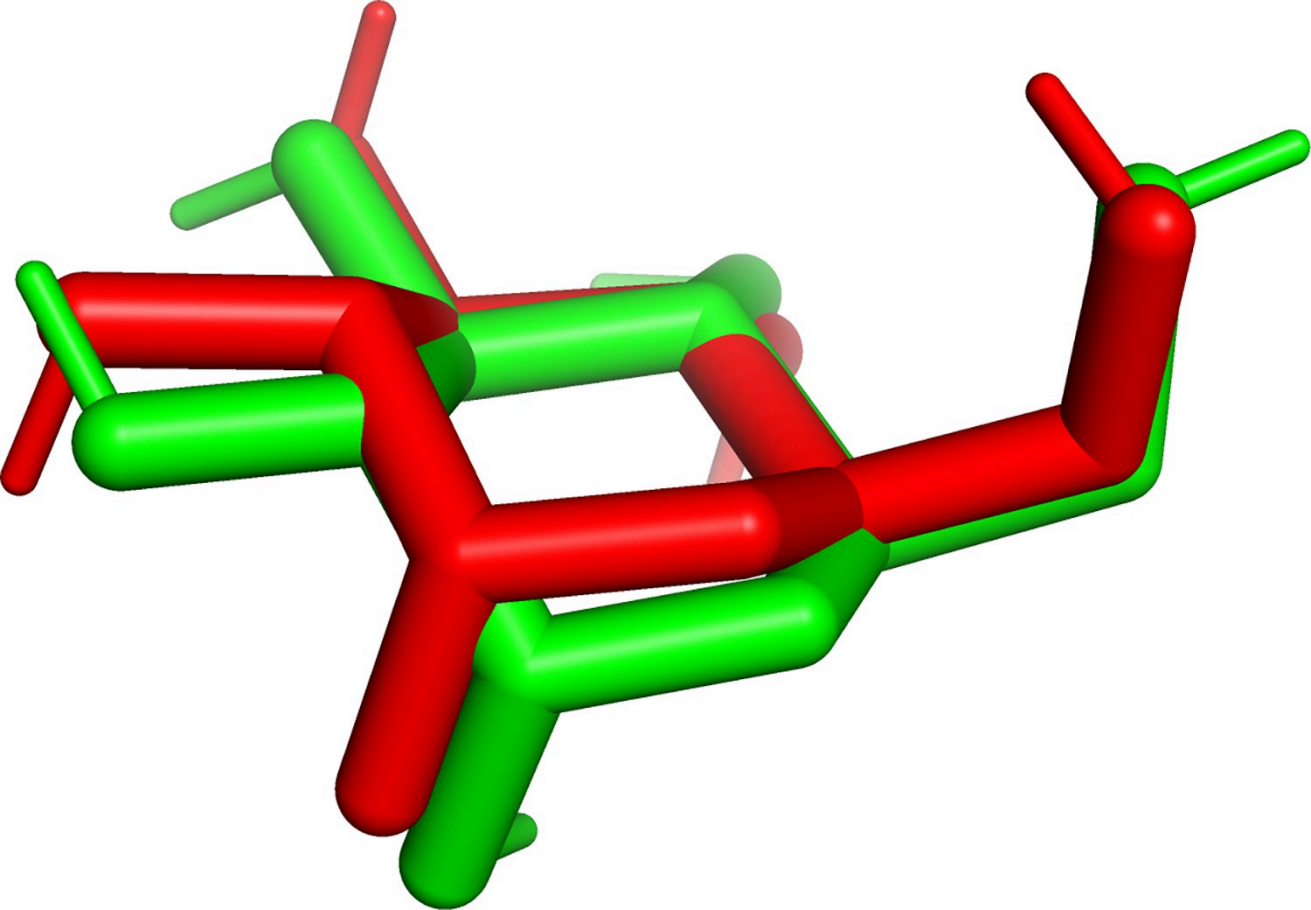
Comparison between the actual’s (from X-ray diffraction, in red color) and redocking’s (in green color) orientation and position of the native ligand, glucose, at 3A4A receptor.

For molecular docking simulation to receptor 3A4A, the grid box used as the place where the docking or binding was simulated, was set to cover the binding site of the native ligand of this receptor, which was glucose. The grid box center was located at coordinate X = 21.1; Y = -7.4; and Z = 24.2; the grid box size was 17 Å × 17 Å × 17 Å, and the grid size was 0.375 Å. For the molecular docking simulation, Autodock Vina was used with the following parameters: exhaustiveness = 64, number of modes (position and orientation which was simulated) = 50, and energy range = 4. This set of parameters and grid box settings were validated by redocking result of the native ligand, glucose, which had RMSD = 0.561 Å ≤ 2 Å. Fig 1 indeed shows that the difference between the actual’s (from X-ray diffraction) and redocking’s orientation and position of the native ligand, glucose, at 3A4A receptor, was indeed small.

**Fig 2:**
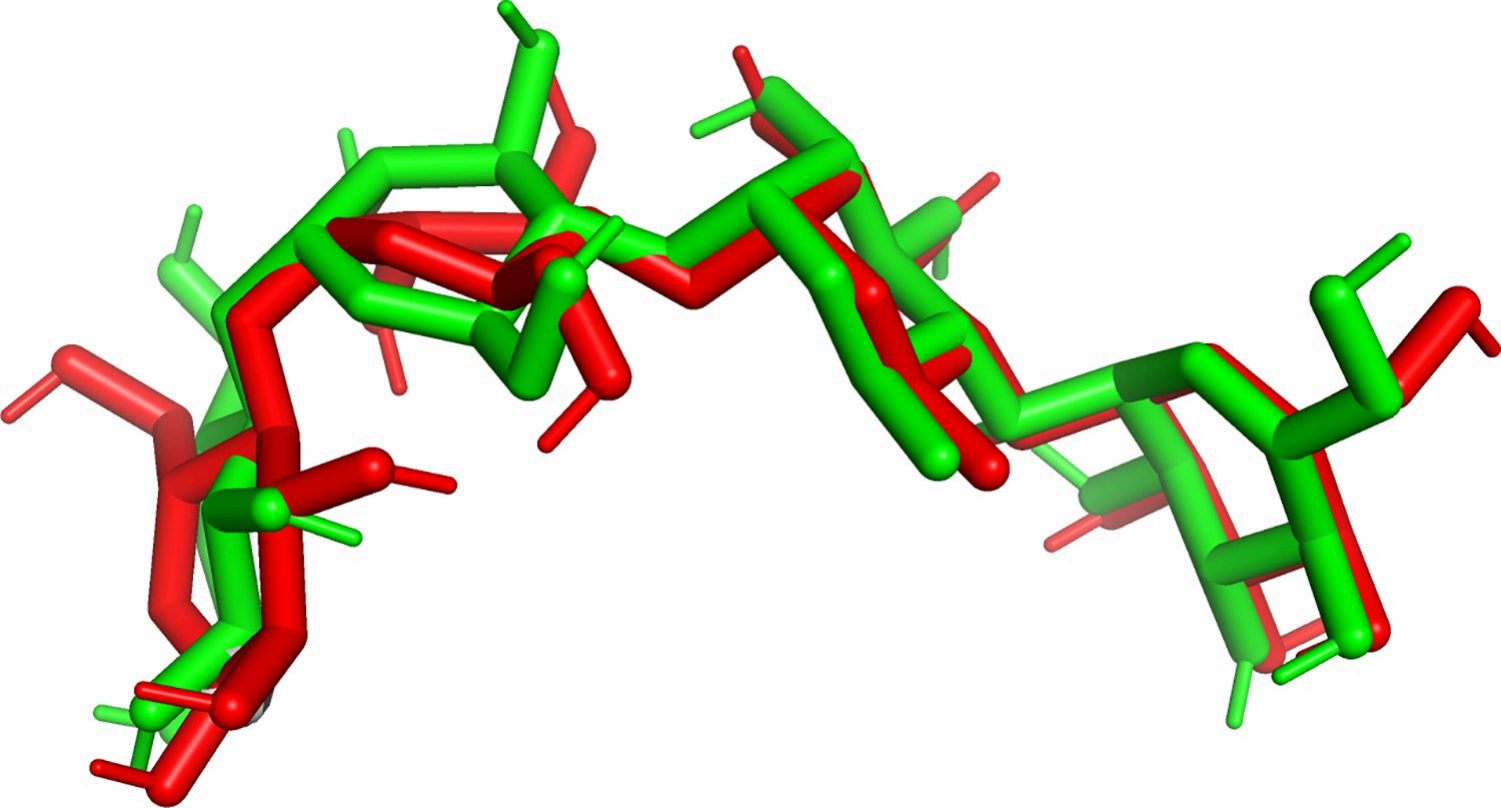
Comparison between the actual’s (from X-ray diffraction, in red color) and redocking’s (in green color) orientation and position of the native ligand, acarbose, at 3TOP receptor.

For the receptor 3TOP, the grid box also covered the binding site of the native ligand, which in this case was acarbose. The grid box had its center located at coordinate X = -31.324; Y = 34.587; and Z = 26.317 and had the size of 40 Å × 40 Å × 40 Å, with grid size = 0.375 Å. The molecular docking simulation was also done with Autodock Vina, with the same parameters as for the receptor 3A4A, except the exhaustiveness was set to 32 for better redocking result (smaller RMSD). The choices of these parameters and grid box were validated by the results of the redocking of acarbose, the native ligand, which had RMSD = 1.096 Å ≤ 2 Å. The comparison between the actual’s and redocking’s orientation and position of acarbose as native ligand to 3TOP receptor in Fig 2 shows that the difference was indeed small.

### Isolation of active compound

To verify the results of metabolomics analysis, isolation of the active compounds was done on the *Artabotyrs sumatranus* leaf extract using bioassay guided fractionation [52, 53]. The 225 g of *Artabotrys sumatranus* dried leaf powder was mixed with 100% ethanol with proportion 1:5, macerated for 24 hours, and then filtered. The maceration process was repeated twice. The filtrate was collected and evaporated using a rotary evaporator resulting in the ethanol crude extract. After that, solid-liquid partitions using n-hexane, ethyl acetate, and ethanol were done to get the n-hexane, ethyl acetate, and ethanol fractions. Each of the fractions was tested for its α-glucosidase inhibition activity. The ethanol fraction which had the highest inhibition percentage of α-glucosidase was put in the silica column with mobile phase a combination of ethyl acetate and methanol using a gradient system (ethyl acetate–methanol proportion was changed from 1:0 to 0:1). The volume of each combination was 500 ml and the flow rate was 2 ml/minutes. The partition of silica column resulted in 15 fractions which were further tested for their activities. Two fractions with the highest α-glucosidase inhibitions were chosen to put in Sephadex LH-20 column using the methanol as the mobile phase. One of the chosen fractions resulted in 20 fractions that contained only minor compounds based on NMR analysis and were not promising to be isolated further. The other fraction resulted in 29 fractions, and based on the similarity in TLC (thin layer chromatography) pattern, some of these fractions were pooled together. The TLC pattern of the pooled fractions was promising to be isolated, and therefore it was evaporated in room temperature, leaving only a small amount of methanol solvent. Then, 2 ml of chloroform was added to the dried pooled fraction, and precipitation was formed, resulting in one isolate, which was then elucidated with 1D and 2D NMR.

For more information, a figure summarizing the isolation process is provided in S1 Fig. The α-glucosidase inhibition bioactivity assay results for the isolation fractions were also shown in this S1 Fig.

### Nuclear magnetic resonance (NMR)

The isolated samples from bioassay guided fractionation were dissolved in deuterated methanol and put in the FTNMR (Fourier transform nuclear magnetic resonance). The isolate would be analyzed by using 1D NMR (^1^H NMR and ^13^C NMR) and 2D NMR (HMBC (heteronuclear multiple bond correlation) and HSQC (heteronuclear single quantum coherence)). These analyses would elucidate the molecular structure of the isolate.

## Results and discussion

**Fig 3:**
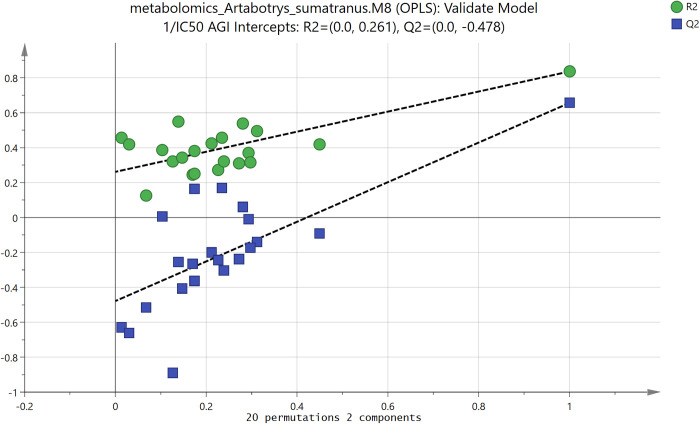
Permutation plot of multivariate statistical model M8, showing the goodness-of-fit coefficients for the permuted training set (R^2^ = R2Y) and permuted test or cross-validated set (Q^2^), with respect to the correlation coefficients of the permuted output Y (1/IC_50_ AGI) to the original data. The values of the output Y were permuted 20 times. The values of R^2^ and Q^2^ of the unpermuted data can be seen at the upper right corner. The model was valid if the values of R^2^ and Q^2^ for the permuted data were significantly lower than for unpermuted one.

For the statistical multivariate approach in metabolomics analysis, all 11 valid models in Table 2 were analyzed. Due to space constraint, here only an example of these analyses is shown, which is for model M8. As can be seen in Table 1, this model used OPLS method and the output variables were 1/IC_50_ AGI (α-glucosidase inhibition) and 1/IC_50_ DPPH (antioxidant activity). Since α-glucosidase inhibition (AGI) activity was the main focus of this research, the analysis shown here is only for the output 1/IC_50_ AGI. The validity of this model could be verified by analyzing the permutation plot of this model (see Fig 3). As can be seen in Fig 3, the values of the goodness-of-fit coefficients for the permuted training set (R^2^ = R^2^_Y_) and permuted test or cross-validated set (Q^2^) were lower than for the unpermuted ones (at the upper right corner). The intercepts of the regression lines for R^2^ and Q^2^ were also under the validity upper thresholds, which were 0.4 and 0.05 respectively. This indicated that the original unpermuted model could fit quite well to the training set, and more importantly to the test set (by way of cross-validation).

**Fig 4:**
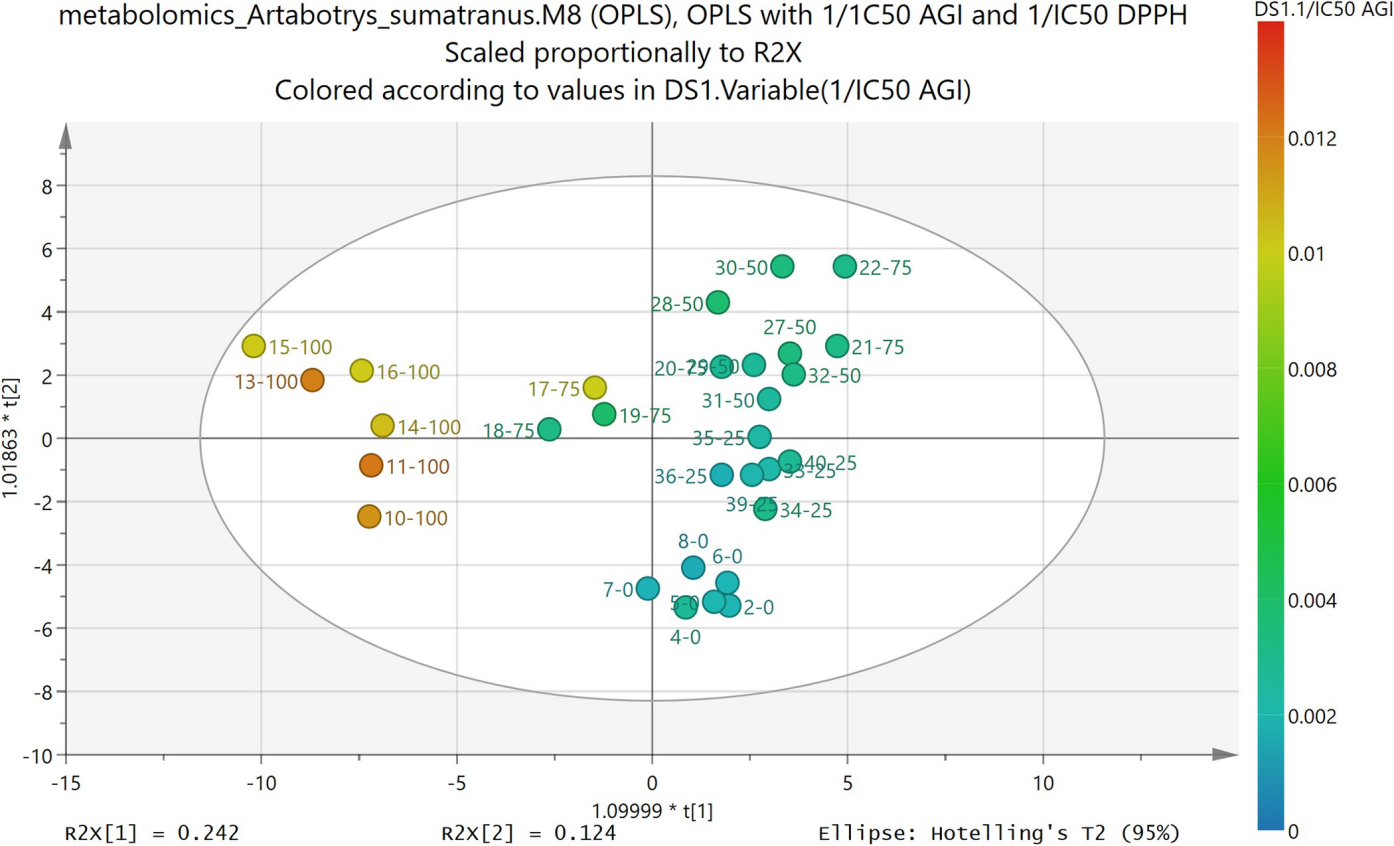
Scoring plot of model M8, which used OPLS method, with input variable X (feature) the values of m/z of the detected compounds in extract samples, and output variable Y (target) the values of 1/IC_50_ AGI and 1/IC_50_ DPPH. Labels showed the fraction names (the number after the dash line showed the percentage of ethanol in the solvent). The color was according to the values of 1/IC_50_ AGI.

Since the validity of model M8 could be assured, the analysis of this model could go further. The scoring plot of this model can be seen in Fig 4, which showed that model M8 separated the active and non-active samples mainly by the first principal component (PC1, or in Fig 4 the horizonal (t[1]) axis). The smaller the values of PC1, the more active the extract samples. The vertical (t[2]) axis showed the second principal component (PC2), which seemed to separate the samples more on their polarity (small or negative values of PC2 corresponded to samples in solvents with 100% water or 0% ethanol, while large values of PC2 corresponded to samples in solvents with more percentage of ethanol). Therefore, for the α-glucosidase activity, small PC1 was preferred.

**Fig 5:**
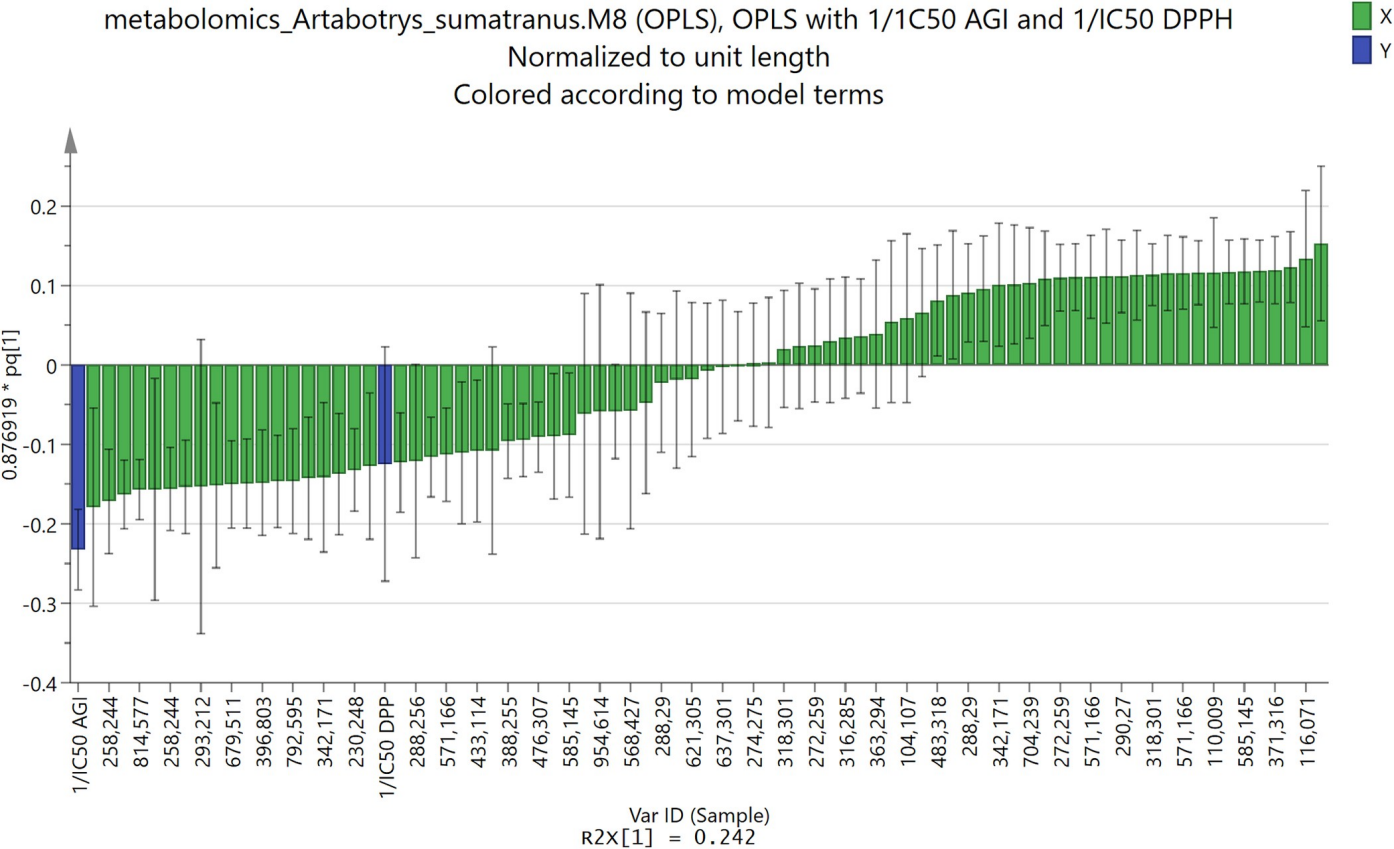
Loadings plot of the first principal component (PC1) of model M8, which used OPLS method, with horizontal axis the input variables X (feature) i.e., the values of m/z of the detected compounds in extract samples, and the vertical axis the loadings value. The output variables Y (target) of the model were the values of 1/IC_50_ AGI and 1/IC_50_ DPPH.

The conclusion of the analysis of the scoring plot in Fig 4 guided the determination of predicted active compounds from loadings plot. The loadings plot of the first principal component (PC1) was shown in Fig 5. Due to the conclusion of the analysis of the scoring plot, the active compounds should be found from the features (input variables, m/z values) which made the PC1 small, in other words, the features that had small or even negative loadings values of PC1. By cross-checking between the most negative features and the VIP values of the corresponding features (VIP values must be larger than 1), 25 most important features were selected. The same procedure was done for the other valid models in Table 2.

Collecting all the features which most often appeared (more than 50% appearances) in the lists of most important features from all valid multivariable statistical methods, a list of predicted active compounds (in the form of m/z values) was constructed. This list could be seen in Table 3, and it served as the prediction of active compounds from statistical multivariate analysis. Most of the active compounds appeared quite often in the statistical multivariate models. There were some compounds, such as compounds represented by Var43 and Var47 –Var 50, which always appeared in every model. This list would be later compared with the predicted active compound list from machine learning approach, to create the predicted active compounds of the metabolomic analysis.

**Table 3:** List of predicted active compounds from multivariate statistical analysis, constructed from the features that most often appeared in the lists of predicted compounds of the 11 valid multivariate statistical analyses. The compounds were ordered by the variable ID.

No.	Variable ID	m/z values	Percentage of appearances in the multivariate statistical models
1	Var54	595.165	90.91
2	Var50	293.212	100.00
3	Var49	328.155	100.00
4	Var48	203.18	100.00
5	Var47	433.114	100.00
6	Var46	279.232	90.91
7	Var45	423.093	90.91
8	Var44	328.155	90.91
9	Var43	342.171	100.00
10	Var42	258.244	72.73
11	Var31	585.145	100.00
12	Var19	258.244	90.91
13	Var09	195.087	81.82

The first analysis for machine learning approach was to find the correlations between the features and activities, both 1/IC_50_ AGI and 1/IC_50_ DPPH. A list of 25 features which had the highest correlation coefficients to 1/IC_50_ AGI, and another for 1/IC_50_ DPPH case, were created. The list can be seen in S3 Table. The correlation list to 1/IC_50_ AGI was used to determine the active compound prediction for machine learning approach, while the one to 1/IC_50_ DPPH was used to get some insights whether the active compounds for AGI were also antioxidants. In general, many features (i.e. compounds) which had high positive correlation to 1/IC_50_ AGI, also had high correlation to 1/IC_50_ DPPH. This indicated that α-glucosidase inhibitors in the extract were likely to have antioxidant activities too, supporting the results obtained from the screening of this plant [16].

The performance results of the machine learning methods to predict the activity of the extract samples as AGI can be seen in Table 4. Here linear regression was added as a comparison.

**Table 4:** Performance results of the prediction of the 1/IC_50_ AGI values of extract samples by machine learning methods for training sets and test sets, using RMSE (root mean square error) and R^2^ as indicators. Small value of RMSE and value of R^2^ near 1 indicated good performance.

No	Method	Test on training set		Test on test set	
		RMSE	R^2^	RMSE	R^2^
1	Linear regression	0.000	1.000	0.003	0.428
2	Decision tree	0.000	0.988	0.002	0.535
3	Random forest	0.001	0.927	0.002	0.603
4	Gradient boosting	0.000	1.000	0.002	0.543
6	ADA boost	0.000	0.999	0.002	0.561

As can be seen in Table 4, the performances for training set were understandably better than for test set, since the models were trained on the training set. What was more important was the performances for test set. Here random forest was the best. The differences between the performances for the training set and test set were also in general smallest for random forest. This was considered good, since large difference between performances for training and test sets indicated the possibility of overfitting, where the model fitted too much on the training set, and therefore lost some of its capability to capture the pattern of the general data.

Since the performances of the machine learning models on test set were rather poor, bootstrapping (using stratified random sampling with replacements) was tried to increase the number of extract samples which was indeed small (only 30). Increasing the number of data can increase the performance of the machine learning methods. Bootstrapping increased the number of samples to 50. The performances of the machine learning models were measured again with the results shown in Table 5.

**Table 5:** Performance results of the prediction of the 1/IC_50_ AGI values of extract samples by machine learning methods, after the implementation of bootstrapping, for training sets and test sets, using RMSE (root mean square error) and R^2^ as indicators. Small value of RMSE and value of R^2^ near 1 indicated good performance.

No	Method	Test on training set		Test on test set	
		RMSE	R^2^	RMSE	R^2^
1	Linear regression	0.000	1.000	0.003	0.173
2	Decision tree	0.000	0.998	0.004	-0.809
3	Random forest	0.000	0.989	0.001	0.791
4	Gradient boosting	0.000	1.000	0.002	0.632
6	ADA boost	0.000	1.000	0.002	0.748

Bootstrapping indeed increased the performance of the machine learning methods on training set (see Table 5). But on the test set, only random forest, gradient boosting, and ADA boost improved the performance. Linear regression and decision tree showed worse performance than without bootstrapping, which indicated the occurrence of overfitting, since the performances on test set were better. Random forest still showed the best performance on test set, and therefore in this research random forest was used for the machine learning approach.

The random forest models, both using bootstrapping and not, were then analyzed for their most important features to obtain the predicted active compounds. The methods which were used to obtain the most important features were random permutation of the features and SHAP. The applications of these 2 methods on the random forest models (with and without bootstrapping) resulted in 4 lists, each containing 25 most potential features, ordered by the mean of increase of RMSE (root mean error) for random permutation method and by the SHAP score for SHAP method. The SHAP scores were computed as the mean of absolute values of the Shapley scores for each extract sample. These lists can be seen in S4 Table.

Comparison was made between the results of feature importance analysis using random permutation and SHAP methods for the random forest models (with and without bootstrapping, total 4 results) and correlation analysis of the features to 1/IC_50_ AGI (α-glucosidase inhibition). The results of correlation (only the highest positive correlations) and feature importance analyses can be seen in S3 and S4 Tables respectively. Some features that appeared in feature importance results, i.e. Var5, Var8, and Var11, were removed during this comparison since these features had negative correlations to 1/IC_50_ AGI (not shown in S3 Table which only showed the features with highest positive correlations). Cross-checking with results of multivariate statistical models, these 3 features or variables indeed had large negative influences to predicted activity output (had large weights in the loadings of the relevant principal components, but the weights contributed to the decrease instead of increase of the activity output). Feature importance analysis of the machine learning models turned out to be able to capture the features which were important in the negative sense also. Features that appeared in the feature importance analyses and also showed strong positive correlation to 1/IC_50_ AGI constructed the list of predicted active compounds from machine learning models (see Table 6). Like for multivariate statistical results, the predicted active compounds were taken from features that had percentage of appearances ≥ 50%.

**Table 6:** List of predicted active compounds from machine learning models, constructed from the features that most often appeared in the lists of predicted compounds of feature importance analyses and in the list of features that had the strongest positive correlation to 1/IC_50_ AGI (α-glucosidase inhibition). The compounds were ordered by the variable ID.

No.	Variable ID	m/z value	*Percentage of appearances in machine learning models and correlation analysis*
1	Var55	593.276	60
2	Var51	621.305	60
3	Var50	293.212	100
4	Var49	328.155	100
5	Var48	203.18	100
6	Var47	433.114	60
7	Var46	279.232	100
8	Var45	423.093	60
9	Var44	328.155	60
10	Var43	342.171	80
11	Var42	258.244	100
12	Var31	585.145	80
13	Var30	701.493	60
14	Var28	814.577	60
15	Var27	905.679	60
17	Var21	288.256	60
18	Var02	290.27	80

The results of multivariate statistical (Table 3) and machine learning models (Table 6) were compared. The differences between multivariate statistical and machine learning results might be because multivariate statistical models were linear models, while machine learning (here random forest) models could be non-linear. The features that appeared in both results were taken as the predicted compounds of the metabolomic analysis. Combining the results of these methods together, and making the final predictions through voting (taking the predictions that appeared in all methods) should increase the accuracy of the final prediction. This idea of combining models and doing voting among the models to get the final prediction was inspired by the ensemble methods in machine learning, such as random forest [46, 47]. Random forest, which is a combination of many decision tree models, has been shown to show better accuracy than the individual decision tree [46].

In an effort to identify these predicted compounds, LC-MS/MS were done to get the fragmentation patterns of the corresponding m/z signals. By analyzing the fragmentation patterns and using the available database in the LC-MS/MS, some of the predicted compounds could be identified. The list of the predicted compounds, with their identified compound names (if available), can be seen in Table 7. The fragmentation patterns of the identified active compounds were analyzed manually to verify the database-based identification. This manual analysis confirmed the identification of the active compounds, and it can be seen in S1 File. From the 10 m/z values which were predicted to be active as α-glucosidase inhibitors, 6 compounds could be identified.

**Table 7:** List of predicted compounds from metabolomic analysis with their m/z values and identified compound names (if available), sorted by the variable ID.

Variable ID	m/z value	Compound name
31	585.145	neomangiferin
42	258.244	-
43	342.171	lirioferine
44	328.155	norisocorydine
45	423.093	mangiferin
46	279.232	15,16-dihydrotanshinone I
47	433.114	apigenin-7-O-galactopyranoside
48	203.18	-
49	328.155	norisocorydine
50	293.212	-

To verify the activities of the known predicted compounds, molecular docking simulations were done for 2 receptors: 3A4A, the α-glucosidase enzyme in *Saccharomyces cerevisiae* which was usually used in α-glucosidase inhibition bioassay, and 3TOP, a part of the α-glucosidase enzyme found in human gut. The results of the molecular docking analysis for the 6 predicted compounds that could be identified can be seen in Table 8. Here acarbose was added as a comparison.

**Table 8:** Results of molecular docking simulation of predicted active compounds on receptor 3A4A (α-glucosidase enzyme in *Saccharomyces cerevisiae*) and 3TOP (a part of the α-glucosidase enzyme found in human gut). The binding was stronger when the free binding energy was more negative. The value of IC_50_ was obtained from α-glucosidase inhibition bioassay result of the isolated compound from the extract.

No	Compound name	Free binding energy (kcal/mol) to 3A4A receptor	Free binding energy (kcal/mol) to 3TOP receptor	IC_50_ value (ppm)
	Acarbose (as comparison)	-9.156	-8.0942	
1	Mangiferin	-10.3011	-8.7348	83.72
2	Neomangiferin	-5.6295	-8.9733	
3	Apigenin-7-O-galactopyranoside	-12.8365	-9.0971	
4	15,16-dihydrotanshinone I	-8.2679	-9.4811	
5	Norisocorydine	-7.2797	-7.4533	
6	Lirioferin	-9.4422	-8.1709	

**Fig 6:**
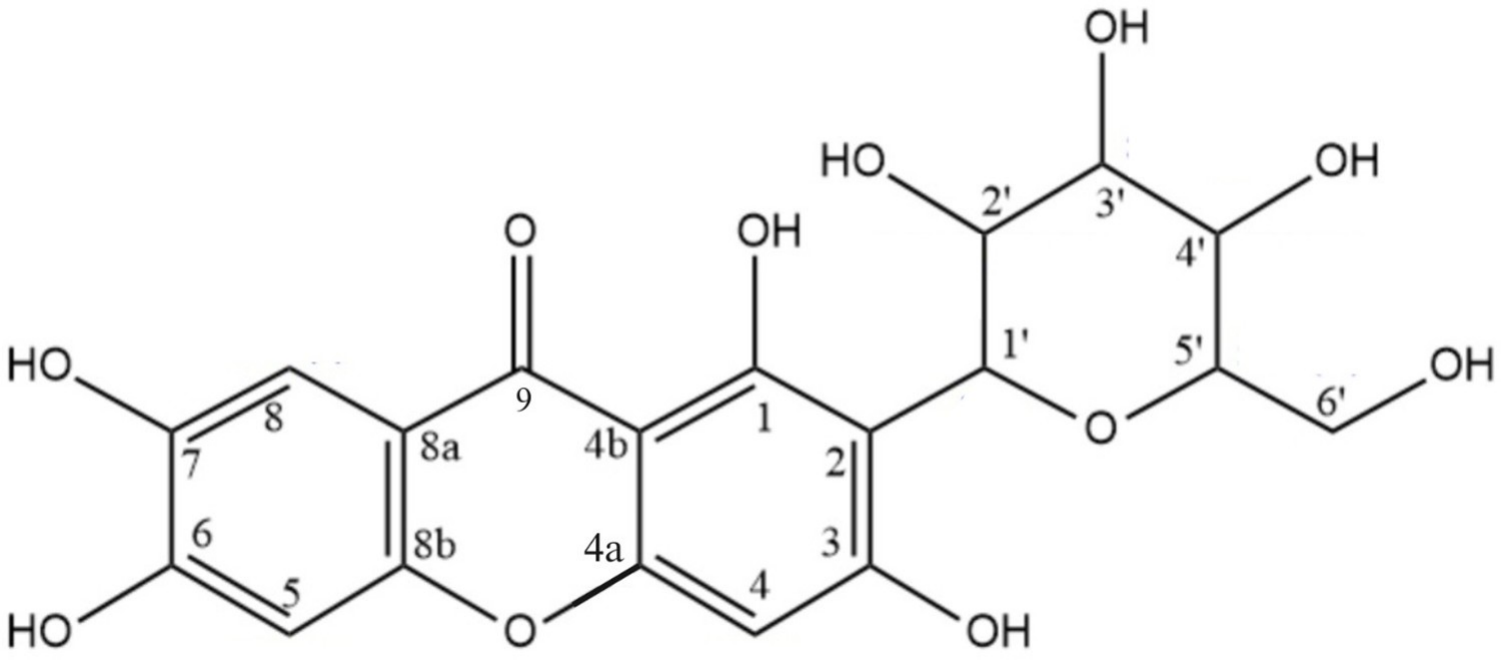
Molecular structure of mangiferin, the isolated compound from *Artabotrys sumatranus* leaf extract.

The value of IC_50_ reported in Table 8 was obtained from α-glucosidase inhibition bioassay test on the compound which was successfully isolated from the extract. The isolation used bioassay guided fractionation and resulted in one isolated compound. The isolated compound structure was elucidated using 1D NMR (^1^H NMR and ^13^C NMR) and 2D NMR (HMBC (heteronuclear multiple bond correlation) and HSQC (heteronuclear single quantum coherence)), and it could be concluded that the isolated compound was mangiferin with the structure as shown in Fig 6. The NMR results and elucidation explanation can be seen in S2 File. The elucidation was also confirmed by elucidation of mangiferin in literatures [74, 75].

The IC_50_ of α-glucosidase inhibition for isolated mangiferin in Table 8 indicated that mangiferin was quite a potent α-glucosidase inhibitor. The free binding energy obtained from molecular docking simulation, also showed that the binding for mangiferin was stronger than acarbose, both to 3A4A and 3TOP receptors. In literature, mangiferin was also reported as potent α-glucosidase inhibitor [76, 77]. Mangiferin was also reported as one of the major compounds in the screening of *Artabotrys sumatranus* leaf extract [16], which might be the reason why this compound was the one that could be isolated.

**Fig 7:**
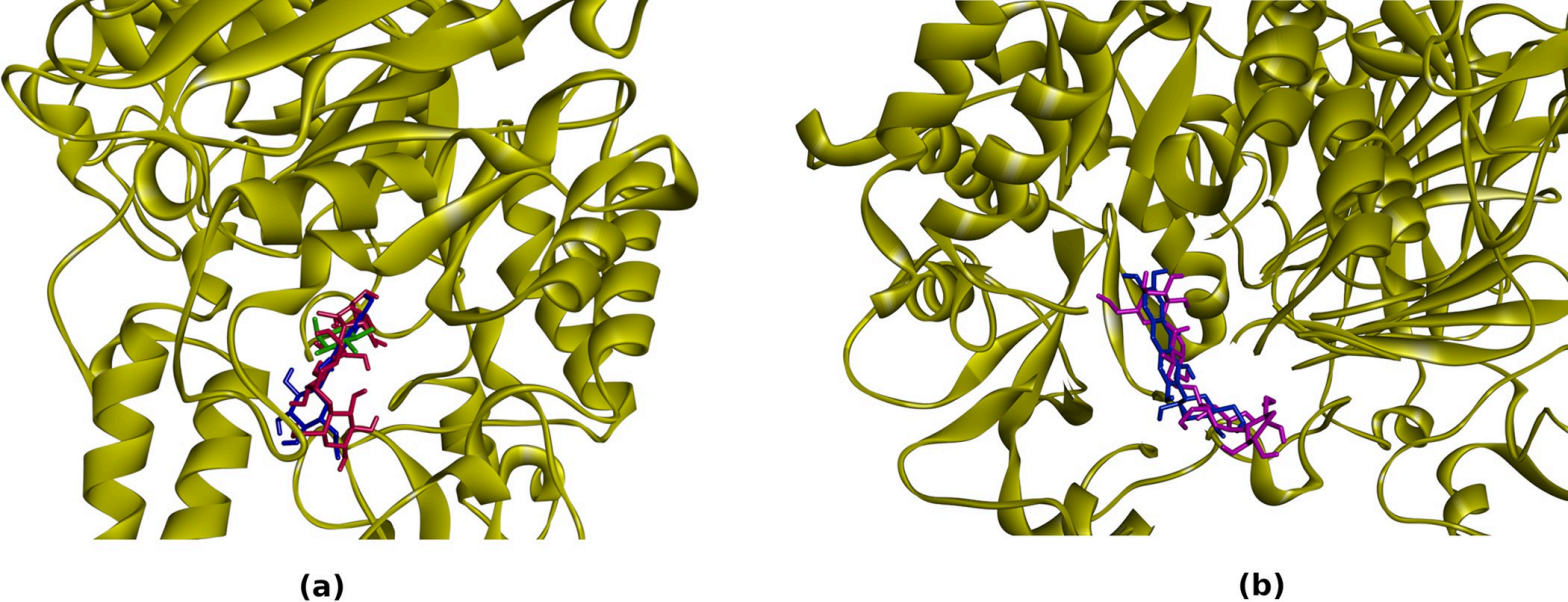
Positions and orientations of molecular docking simulations of mangiferin (blue color) to 3A4A (a) and 3TOP (b) receptors. The position and orientations of the native ligands, which were glucose (green color) in 3A4A receptor and acarbose (purple) in 3TOP receptor were also shown. The position and orientation of acarbose (red) in 3A4A receptor were also shown for comparison.

As shown in Fig 7, in the results of molecular docking simulation, mangiferin occupied the same binding site as the native ligands, both for 3A4A (glucose) and 3TOP (acarbose). Acarbose, which was a known potent α-glucosidase inhibitor and has been used as antidiabetic drug, also occupied the same binding site, not only in 3TOP receptor (where it was the native ligand), but also in 3A4A receptor. This indicated that mangiferin indeed had the potential to become a potent α-glucosidase inhibitor.

**Fig 8:**
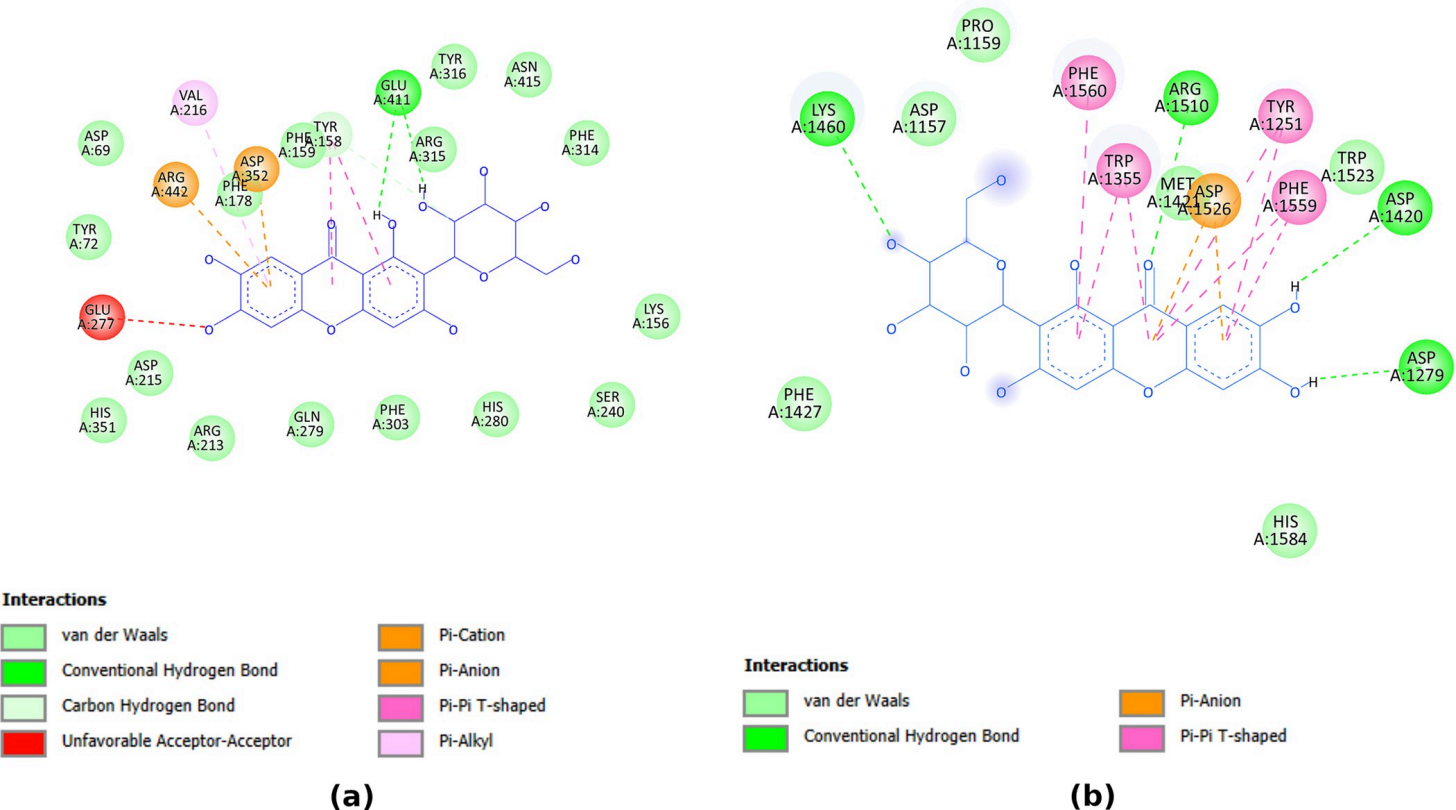
Interactions between mangiferin to the amino acids of the 3A4A (a) and 3TOP (b) receptors.

The interactions of mangiferin to both receptors, 3A4A and 3TOP, showed that this compound had not only weak van der Waals bonds to the amino acids in the receptors, but also some strong hydrogen bonds, beside other kinds of bonds (see Fig 8). This supported the conclusion that mangiferin had strong binding to the receptors.

A more detailed analysis of the molecular interactions can be seen in Table 9 for 3A4A receptor and Table 10 for 3TOP receptor. The information in this Tables 8 and 9 was taken from observations of 2D interactions figures like Fig 8 for all the identified predicted active compounds. In Tables 9 and 10, a summary of the total number of bonds is presented, but actually for each type of bonding, there were bonds which were available for different identified predicted active compounds. The more complete descriptions of the molecular interactions, including the amino acids involved in each type of bonding, can be seen in S5 Table for 3A4A receptor and S6 Table for 3TOP receptor.

**Table 9:** Summary of molecular interactions between identified predicted active compounds with 3A4A receptor.

Type of Bonding	Acarbo-se	Mangi-ferin	Neo-mangi-ferin	15,16-Dihydro-tanshino-ne I	Lirio-ferin	Noriso-corydine	Apigenin-7-O-Galacto-pyrano-side
Total hydrogen bonding	9	1	5	0	3	4	2
Total unfavorable bump	0	0	2	0	0	0	0
Total Van der Waals	7	14	19	8	8	6	17
Total carbon hydrogen bond	3	3	1	0	4	0	1
Total unfavorable acceptor-acceptor	1	1	0	0	0	0	0
Total Unfavorable donor-donor	0	0	2	0	0	0	0
Total pi cation	0	1	0	0	2	2	1
Total pi anion	0	1	0	1	1	1	1
Total pi sigma	0	0	0	0	2	0	0
Total pi-pi stacked	0	0	0	1	0	0	1
Total pi-alkyl	0	1	1	1	1	3	0
Total favorable bonds	19	21	26	11	21	16	23
Total unfavorable bonds	1	1	4	0	0	0	0

**Table 10:** Summary of molecular interactions between identified predicted active compounds with 3TOP receptor.

Type of bonding	Acarbose	Mangi-ferin	Neo-mangi-ferin	15,16-Dihydro-tanshino-ne I	Lirioferin	Noriso-corydine	Apigenin-7-O-Galacto-pyrano-side
Total hydrogen bonding	2	5	1	0	1	0	2
Total Van der Waals	13	8	16	9	9	8	10
Total carbon hydrogen bond	0	0	0	2	1	1	1
Total unfavor-able acceptor-acceptor	0	0	1	0	0	0	1
Total unfavor-able donor-donor	0	0	1	0	0	0	0
Total pi anion	0	1	1	0	0	1	0
Total pi sigma	0	0	0	0	1	1	0
Total pi-pi T-shaped	0	4	2	3	2	2	2
Total pi-alkyl	0	0	0	4	0	1	0
Total salt bridge	1	0	0	0	0	0	0
Total attractive charge	1	0	0	0	0	0	0
Total favorable bonds	17	18	20	18	14	14	15
Total unfavor-able bonds	0	0	2	0	0	0	1

In Table 9, it can be seen that for 3A4A receptor mangiferin, lirioferin, and apigenin-7-O-galactopyranoside had more favorable bonds than acarbose, which corresponded well with the free binding energy of these three compounds which were more negative (stronger binding) than acarbose (see Table 8). Neomangiferin also had more favorable bonds than acarbose, but this compound also had more unfavorable bonds which seemed to make the binding weaker, resulting in more positive free binding energy. 15,16-dihydrotanshinone I and norisocorydine had less favorable bonds than acarbose, which matched well with the more positive free binding energy of these compounds compared to acarbose. Although the distributions of the bonding types were different, all these identified predicted active compound occupied the same binding site in 3A4A receptor like mangiferin and acarbose in Fig 7 (see S3 File). The proportion of hydrogen bonding or other kinds of bonding did not seem to influence the strength of the binding very much. Of course, further investigation is needed to confirm this.

While all the predicted active compounds occupied the same binding site in 3TOP receptor as mangiferin and acarbose (see Fig 7 and S3 File), the distributions of the bonding types were different for the compounds. The bonding summary for 3TOP receptor can be seen in Table 10. Mangiferin and 15,16-dihydrotanshinone I had more favorable bonds than acarbose, which corresponded well to the more negative free binding energies of these compounds compared to acarbose in Table 8. Neomangiferin also had more favorable bonds than acarbose, but it had also more unfavorable bonds. However, in this case it seemed that the unfavorable bonds in neomangiferin did not affect the binding strength very much, since the free binding energy of neomangiferin was still more negative than acarbose. Norisocorydine had fewer favorable bonds than acarbose which matched its more positive free binding energy. Lirioferin had the same number of favorable bonds as norisocorydine, but lirioferine had hydrogen bonding which was not available for norisocorydine. This might be the reason why free binding energy of lirioferin was more negative than norisocorydine. Apigenin-7-O-galactopyranoside had fewer favorable bonds than acarbose, but the free binding energy of this compound was more negative than acarbose. The reason might be in the proportion of bonding types available for apigenin-7-O-galactopyranoside. Further investigation is needed to ascertain the reasons behind the strength of the bindings of these predicted active compounds.

Although the other predicted active compounds were not isolated from the extract in this research, the α-glucosidase inhibition activities of some predicted compounds were reported in literatures, i.e. 15,16-dihydrotanshinone I that had rather strong activity [78] and neomangiferin that had weak activity [77]. The molecular docking results for 15,16-dihydrotanshinone I supported the literature, since the free binding energy for this compound was comparable or even better than acarbose for 3A4A and 3TOP receptors respectively. For neomangiferin, the molecular docking result to 3A4A receptor was in agreement with the literature, but the result to 3TOP receptor indicated that this compound had strong activity as α-glucosidase inhibitor. It might be explained by the fact that in-vitro bioassay test for α-glucosidase inhibition usually uses α-glucosidase enzyme from *Saccharomyces cerevisiae*, the same structure as 3A4A receptor. Then this result indicated that although neomangiferin did not show strong activity in in-vitro test, this compound might actually have strong activity as α-glucosidase inhibitor in human. Although the activity data was from bioassay results (corresponded to 3A4A receptor), metabolomic analysis predicted neomangiferin to be an active compound. This might happen because neomangiferin appeared often together with mangiferin (correlation between neomangiferin (Var31) and mangiferin (Var45) was 0.979, see later in Fig 7). Of course, these indications must be further researched later. The α-glucosidase inhibition activity of the other predicted compounds, i.e. apigenin-7-O-galactopyranoside, norisocorydine, and lirioferin, have not yet been reported. Further investigation and verification of the α-glucosidase inhibition activities of these 3 compounds were needed, especially for apigenin-7-O-galactopyranoside and lirioferin which showed stronger activity than acarbose in molecular docking results.

Beside the identified compounds, the prediction of metabolomics analysis also included some unknown compounds, which were represented by Var50, Var48, and Var42 (see Table 7). These 3 compounds were promising, since they appeared in all machine learning model results (see Table 6). Var50 and Var48 also appeared in the results of all multivariate statistical analysis (see Table 3).

**Fig 9:**
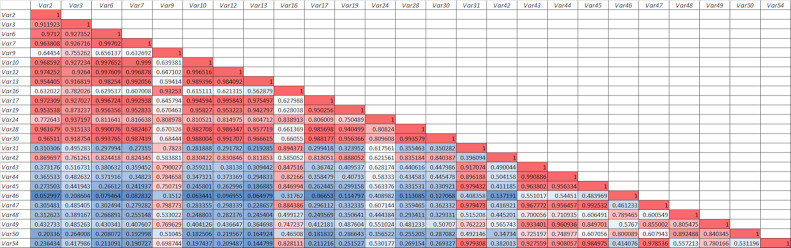
The correlations between 25 features (represents the compounds detected in the *Artabotrys sumatranus* leaf extract) which had the highest positive correlation to 1/IC_50_ AGI (α-glucosidase inhibition). Red colors represent higher (more positive) correlation between the features, while blue color lower correlations. All 25 features had positive correlations to 1/IC_50_ AGI, so that they all had potentials to be active compounds as α-glucosidase inhibitors.

To get more insights about the predicted compounds, the correlations between all the 25 features (representing detected compounds in the extract) that had the highest positive correlations to 1/IC_50_ AGI (α-glucosidase inhibition activity) were investigated (see Fig 9). All the compounds represented by these features had the potentials to become α-glucosidase inhibitors. It turned out that the identified predicted compounds: Var31 (neomangiferin), Var43 (lirioferine), Var44 (norisocorydine), Var45 (mangiferin), Var47 (apigenin-7-O-galactopyranoside) and Var 49 (norisocorydine), were all highly positively correlated. This suggested that these compounds might be related in the biosynthesis pathway. Of course, further research is needed for verification, but this indication is interesting since neomangiferin and mangiferin have indeed similar structures and of the same compound group, xanthone. The unknown predicted compounds represented by Var48 and Var50 were also highly positively correlated, together with Var46 (15,16-dihydrotanshinone I), and also in less strength to Var43 (lirioferine) and Var44 (norisocorydine). This might indicate another biosynthesis pathway group. The last unknown predicted active compound, Var42, had different correlation pattern, suggesting different group of compounds, since it was highly positively correlated to Var2, Var3, Var6, Var7, Var10, Var12, Var13, Var17, Var19, Var28, and Var30. These indications about the biosynthesis pathways must of course be investigated further to get a strong conclusion.

Conclusive results would be obtained if all the predicted compounds could be isolated and tested, especially the unidentified ones. Metabolomics guided fractionation (see for example [79]) might be a good way to isolate the specific compound, since the fractionation will only be continued if the fraction contains the desired chromatogram profiles, for example from NMR or LC-MS, which corresponds to the desired compound. Nevertheless, this procedure will need a lot of NMR or LC-MS analyses (analysis is needed for all fractions), which might not be always available.

To investigate whether the predicted active α-glucosidase inhibitor compounds in *Artabotrys sumatranus* leaf extract also showed antioxidant activity, the correlation of the amounts of the predicted compounds to 1/IC_50_ DPPH (antioxidant activity) was analyzed. The correlation coefficients for the predicted compound to 1/IC_50_ DPPH, beside of course to 1/IC_50_ AGI (α-glucosidase inhibition activity), as a part of S3 Table, can be seen in Table 11.

**Table 11:** Correlation coefficients of the predicted active compounds to 1/IC_50_ AGI (α-glucosidase inhibition) and 1/IC_50_ DPPH (antioxidant).

Variable ID	Compound name (if available)	Correlation coefficient to 1/IC_50_ AGI	Correlation coefficient to 1/IC_50_ DPPH
Var50	-	0.687	0.705
Var49	norisocorydine	0.833	0.686
Var48	-	0.661	0.552
Var47	apigenin-7-O-galactopyranoside	0.566	0.564
Var46	15,16-dihydrotanshinone I	0.494	0.576
Var45	mangiferin	0.581	0.594
Var44	norisocorydine	0.754	0.678
Var43	lirioferine	0.705	0.626
Var42	-	0.649	0.161
Var31	neomangiferin	0.462	0.477

As can be seen in Table 11, all predicted compounds had positive correlations to both 1/IC_50_ AGI. A comparison between Tables 8 and 11 showed that the correlation coefficients to 1/IC_50_ AGI did not correlate perfectly to free binding energy from molecular docking simulation. There were some compounds which had strong free binding energies (very negative), but the correlation coefficients were not very positive. This might be because the correlation only investigated the pattern between the amounts of the compounds to the activity, but not the actual binding strengths. The direct relation to activity was further complicated because there were also correlations between the compounds. Therefore, correlation could be used to give some indications of the activity of the compound, but other analysis was needed to verify it.

In general, the predicted compounds also showed positive correlations to 1/IC_50_ DPPH, indicating that these compounds were also antioxidant. The only exception was the compound represented by Var42, which showed very small positive correlation to 1/IC_50_ DPPH, which supported the deduction of it belonging to different group of compounds from correlation analysis results (see Fig 9). These double activities were also supported by literatures. Mangiferin was reported to show antioxidant activity [77, 80], as well as 15,16-dihydrotanshinone I [81] and norisocorydine [82]. The antioxidant activity of neomangiferin was also reported, albeit weak [77]. For the other predicted compounds, no literatures about their antioxidant activities have been found. The existence of double activities in *Artabotrys sumatranus* leaf extract was further supported by the correlations between α-glucosidase inhibition (AGI) and antioxidant (DPPH) activities in this extract, as can be seen in Table 12. The correlations between these two activities were quite strong, namely 0.73 (when comparing 1/IC_50_) or 0.82 (when comparing IC_50_). This verified the prediction that α-glucosidase inhibitors in *Artabotrys sumatranus* leaf extract had also antioxidant activities in the screening process [16].

**Table 12:** Correlations between α-glucosidase inhibition (AGI) and antioxidant (DPPH) activities in *Artabotrys sumatranus* leaf extract.

	1/IC_50_ AGI	IC_50_ AGI	1/IC_50_ DPPH	IC_50_ DPPH
1/IC_50_ AGI	1			
IC_50_ AGI	-0.873379721	1		
1/IC_50_ DPPH	0.726829502	-0.838292965	1	
IC_50_ DPPH	-0.635525608	0.822423167	-0.959618582	1

## Conclusion

Metabolomics analysis, using combined multivariate statistical and machine learning analyses, predicted 9 active compounds as α-glucosidase inhibitors in the leaf extract of *Artabotrys sumatranus*. Several machine learning methods were investigated, and it turned out the random forest delivered the best model to predict the activity of the extract samples. Feature importance analysis using random feature permutation and SHAP methods were used to identify the most important features that influenced the prediction model of the activity (i.e., the predicted active compounds) of the extract samples. Six of these compounds could be identified using LC-MS/MS fragmentation analysis and database: mangiferin, neomangiferin, norisocorydine, apigenin-7-O-galactopyranoside, lirioferine, and 15,16-dihydrotanshinone I. The α-glucosidase inhibition activities of norisocorydine, apigenin-7-O-galactopyranoside, and lirioferine were not yet reported in literatures. Molecular docking simulations, both to 3A4A receptor (α-glucosidase enzyme in *Saccharomyces cerevisiae*, which was used in bioassay for α-glucosidase inhibition activity) and 3TOP receptor (a part of α-glucosidase enzyme in human gut), verified the metabolomics analysis results by showing strong binding between the identified predicted active compounds to the receptors. Some even showed stronger binding then acarbose, the known potent α-glucosidase inhibitor. The exception was neomangiferin, that had rather weak binding to 3A4A receptor, although it showed strong binding to 3TOP receptor. Isolation using bioassay guided fractionation succeeded in isolating mangiferin, which was elucidated using 1D and 2D NMR. The isolated mangiferin showed strong activity for α-glucosidase inhibition in bioassay test, verifying the metabolomics analysis results.

Although further analysis was needed to verify it, the correlation analysis indicated that there were 3 groups in the predicted active compounds, which might be related to the biosynthesis pathways. Correlation analysis of the predicted compounds also showed that these compounds in general also had potentials to be antioxidant. In general, α-glucosidase inhibition activity in *Artabotrys sumatranus* leaf extract correlated to antioxidant activity, supporting the screening results done in previous research [16].

Further research was needed to verify the activity the predicted active compounds beside mangiferin, perhaps by metabolomics guided isolation. The prediction of the m/z values of the unidentified active compounds from this research should help the further investigation.

## Supporting information

S1 Table List of IC_50_ values for α-glucosidase inhibition (AGI) activity and antioxidant activity (DPPH assay) for the extracts of *Artabotrys sumatranus* leaf using mixture of ethanol and water with different proportions. (PDF)

S2 Table List of variables (features) in the input data for metabolomics analysis. Each feature represented the m/z value of the detected compound in the extract samples of *Artabotrys sumatranus* leaf.
(PDF)

S3 Table List of variables (features) that showed the highest correlations between their amounts (response in LC-MS) to 1/IC_50_ AGI (for the α-glucosidase inhibition–AGI) and 1/IC_50_ DPPH (for the antioxidant). Each feature represented the m/z value of the detected compound in the extract samples of *Artabotrys sumatranus* leaf.
(PDF)

S4 Table List of most influential variables (features) to the prediction 1/IC_50_ AGI (α-glucosidase inhibition) for random forest models: (a) with bootstrapping and (b) without bootstrapping. Each random forest models were analyzed using random permutation and SHAP methods. Variables are ordered based on the mean of RMSE (root mean square error) for random permutation results (mean ± standard deviation should not change the ordering). For SHAP results, the variables are ordered based on the SHAP scores. The smaller the ordering number, the more influential the variables are.
(PDF)

S5 Table Molecular interactions between identified predicted active compounds and 3A4A receptor. The entries in the table show the amino acids of 3A4A receptor which were involved in the bonding. The amino acids are arranged so that same kind of interactions which appeared in other identified predicted active compounds can be recognized.
(PDF)

S6 Table Molecular interactions between identified predicted active compounds and 3TOP receptor. The entries in the table show the amino acids of 3TOP receptor which were involved in the bonding. The amino acids are arranged so that same kind of interactions which appeared in other identified predicted active compounds can be recognized.
(PDF)

S1 Fig The workflow of the isolation of active compound (mangiferin) from *Artabotrys sumatranus* leaf extract using bioassay guided fractionation. The amount and α-glucosidase inhibition bioassay results for the isolation fractions are shown in this figure.
(PDF)

S1 File Analysis of fragmentation patterns of the predicted active compound using LC-MS/MS. In this file the analysis of the fragmentation patterns is presented for m/z values of the compounds which were predicted to be active by the metabolomics analysis. The fragmentation patterns were obtained from LC-MS/MS. The analysis results verify the identification of the active compounds.
(PDF)

S2 File Elucidation of mangiferin as isolated compound from *Artabotrys sumatranus* leaf extract. This file contains the results of 1D NMR (^1^H NMR and ^13^C NMR) and 2D NMR (HMBC (heteronuclear multiple bond correlation) and HSQC (heteronuclear single quantum coherence)) of the isolated compound from *Artabotrys sumatranus* leaf extract. The elucidation analysis is also explained.
(PDF)

S3 File Binding sites of 15,16 dihydrotanshinone I, neomangiferin, apigenin-7-O-galactopyranoside, lirioferin, and norisocorydine to 3A4A and 3TOP receptors. In this file, the binding sites of the identified predicted active compounds to 3A4A receptors and 3TOP receptors are shown. As comparison, the position and orientation of acarbose as a result of molecular docking are shown. Acarbose occupied the same binding site as the native ligands of the 3A4A and 3TOP receptors.
(PDF)
