# Supplementary material for: Investigation of alpha-glucosidase inhibition activity of Artabotrys sumatranus leaf extract using metabolomics, machine learning and molecular docking analysis
Source: PLoS One. 2025 Jan 3;20(1):e0313592. doi: 10.1371/journal.pone.0313592 (PMC11698457; doi:10.1371/journal.pone.0313592)
Supplement: S1 Table — (PDF) [file pone.0313592.s001.pdf]

S1 Table. List of IC_{50} values for α-glucosidase inhibition (AGI) activity and antioxidant activity (DPPH assay) for the extracts of *Artabotrys sumatranus* leaf using mixture of ethanol and water with different proportions

No.	Percentage of ethanol in extract's solvent	IC_{50} α-glucosidase inhibition activity (ppm)	IC_{50} DPPH (ppm)
1	0	535.9446	150.4634
2	0	366.9098	141.9028
3	0	516.3859	154.0357
4	0	570.5355	171.6895
5	0	608.9071	150.5686
6	0	683.8981	150.6798
7	100	86.74679	69.42137
8	100	82.55856	63.12249
9	100	83.43271	61.05087
10	100	95.12978	58.29519
11	100	97.77399	58.47412
12	100	98.07534	47.60652
13	75	99.93273	54.6578
14	75	313.5767	96.36911
15	75	254.708	97.49018
16	75	358.7376	74.29398
17	75	320.2974	70.72764
18	75	327.4263	69.865
19	50	295.5786	70.61383
20	50	263.3356	63.94788
21	50	381.665	67.1256
22	50	293.9382	68.93707
23	50	396.2529	68.2968
24	50	318.1951	70.587
25	25	466.6613	111.6605
26	25	321.2722	114.4792
27	25	433.1374	100.6633
28	25	627.1589	120.5535
29	25	491.0832	130.9444
30	25	364.2273	120.7659
