# Supplementary material for: Investigation of alpha-glucosidase inhibition activity of Artabotrys sumatranus leaf extract using metabolomics, machine learning and molecular docking analysis
Source: PLoS One. 2025 Jan 3;20(1):e0313592. doi: 10.1371/journal.pone.0313592 (PMC11698457; doi:10.1371/journal.pone.0313592)
Supplement: S2 Table — Each feature represented the m/z value of the detected compound in the extract samples of Artabotrys sumatranus leaf. (PDF) [file pone.0313592.s002.pdf]

S2 Table. List of variables (features) in the input data for metabolomics analysis. Each feature represented the m/z value of the detected compound in the extract samples of *Artabotrys sumatranus* leaf

Variable	m/z	Variable	m/z	Variable	m/z	Variable	m/z
Var01	274.275	Var21	288.256	Var41	344.316	Var61	274.275
Var02	290.27	Var22	371.316	Var42	258.244	Var62	230.249
Var03	230.248	Var23	476.307	Var43	342.171	Var63	288.29
Var04	104.107	Var24	338.342	Var44	328.155	Var64	318.301
Var05	116.071	Var25	318.301	Var45	423.093	Var65	272.259
Var06	679.511	Var26	272.259	Var46	279.232	Var66	704.239
Var07	396.803	Var27	905.679	Var47	433.114	Var67	433.114
Var08	138.055	Var28	814.577	Var48	203.18	Var68	342.171
Var09	195.087	Var29	331.285	Var49	328.155	Var69	423.093
Var10	792.595	Var30	701.493	Var50	293.212	Var70	290.27
Var11	110.009	Var31	585.145	Var51	621.305	Var71	585.145
Var12	340.26	Var32	483.318	Var52	568.427	Var72	328.155
Var13	453.344	Var33	288.254	Var53	568.427	Var73	595.165
Var14	288.29	Var34	564.359	Var54	595.165	Var74	571.166
Var15	363.294	Var35	288.29	Var55	593.276	Var75	338.342
Var16	571.166	Var36	107.049	Var56	954.614	Var76	371.316
Var17	566.428	Var37	104.107	Var57	288.29	Var77	595.165
Var18	316.322	Var38	595.166	Var58	704.239	Var78	302.27
Var19	258.244	Var39	316.321	Var59	637.301	Var79	363.294
Var20	388.255	Var40	316.285	Var60	621.307	Var80	571.166
