# Supplementary material for: Investigation of alpha-glucosidase inhibition activity of Artabotrys sumatranus leaf extract using metabolomics, machine learning and molecular docking analysis
Source: PLoS One. 2025 Jan 3;20(1):e0313592. doi: 10.1371/journal.pone.0313592 (PMC11698457; doi:10.1371/journal.pone.0313592)
Supplement: S3 Table — Each feature represented the m/z value of the detected compound in the extract samples of Artabotrys sumatranus leaf. (PDF) [file pone.0313592.s003.pdf]

S3 Table. List of variables (features) that showed the highest correlations between their amounts (response in LC-MS) to 1/IC_{50} AGI (for the α-glucosidase inhibition – AGI) and 1/IC_{50} DPPH (for the antioxidant). Each feature represented the m/z value of the detected compound in the extract samples of *Artabotrys sumatranus* leaf

No	Correlation to 1/IC_{50} AGI		Correlation to 1/IC_{50} DPPH	
	Variable ID	Correlation coefficient	Variable ID	Correlation coefficient
1	Var49	0.833	Var50	0.705
2	Var44	0.754	Var49	0.686
3	Var43	0.705	Var44	0.678
4	Var50	0.687	Var43	0.626
5	Var48	0.661	Var45	0.594
6	Var42	0.649	Var46	0.576
7	Var45	0.581	Var47	0.564
8	Var47	0.566	Var48	0.552
9	Var9	0.546	Var54	0.519
10	Var30	0.531	Var31	0.477
11	Var28	0.508	Var9	0.377
12	Var19	0.507	Var58	0.295
13	Var46	0.494	Var36	0.285
14	Var54	0.491	Var53	0.281
15	Var12	0.489	Var16	0.28
16	Var6	0.471	Var56	0.277
17	Var2	0.464	Var52	0.255
18	Var31	0.462	Var38	0.225
19	Var7	0.461	Var35	0.189
20	Var10	0.452	Var55	0.168
21	Var16	0.452	Var66	0.161
22	Var17	0.452	Var25	0.161
23	Var13	0.425	Var42	0.161
24	Var24	0.42	Var72	0.157
25	Var3	0.415	Var68	0.142
