# Supplementary material for: Investigation of alpha-glucosidase inhibition activity of Artabotrys sumatranus leaf extract using metabolomics, machine learning and molecular docking analysis
Source: PLoS One. 2025 Jan 3;20(1):e0313592. doi: 10.1371/journal.pone.0313592 (PMC11698457; doi:10.1371/journal.pone.0313592)
Supplement: S4 Table — List of most influential variables (features) to the prediction 1/IC50 AGI (α-glucosidase inhibition) for random forest models: (a) with bootstrapping and (b) without bootstrapping. Each random forest models were analyzed using random permutation and SHAP methods. Variables are ordered based on the mean of RMSE (root mean square error) for random permutation results (mean ± standard deviation should not change the ordering). For SHAP results, the variables are ordered based on the SHAP scores. The smaller the ordering number, the more influential the variables are. (PDF) [file pone.0313592.s004.pdf]

S4 Table. List of most influential variables (features) to the prediction 1/IC_{50} AGI (α-glucosidase inhibition) for random forest models: (a) with bootstrapping and (b) without bootstrapping. Each random forest models were analyzed using random permutation and SHAP methods. Variables are ordered based on the mean of RMSE (root mean square error) for random permutation results (mean ± standard deviation should not change the ordering). For SHAP results, the variables are ordered based on the SHAP scores. The smaller the ordering number, the more influential the variables are.

No.	Random permutation			SHAP	
	Variable	Mean	Standard deviation	Variable	Score
1	Var49	0.000515	0.000194	Var76	0.000508
2	Var76	0.000221	0.000159	Var49	0.000485
3	Var8	0.000174	6.69E-05	Var75	0.000394
4	Var50	0.000101	3.76E-05	Var28	0.0003
5	Var48	5.19E-05	2.04E-05	Var8	0.000229
6	Var46	4.11E-05	1.69E-05	Var50	0.000154
7	Var11	2.55E-05	1.33E-05	Var33	0.000134
8	Var42	2.39E-05	2.71E-05	Var46	9.78E-05
9	Var33	1.66E-05	4.26E-05	Var42	9.07E-05
10	Var30	1.51E-05	1.92E-05	Var48	8.87E-05
11	Var27	1.25E-05	6.25E-06	Var30	6.41E-05
12	Var43	1.13E-05	1.36E-05	Var21	6.32E-05
13	Var9	1.04E-05	1.7E-05	Var45	6.2E-05
14	Var44	1.02E-05	1.74E-05	Var11	4.66E-05
15	Var51	7.2E-06	2.73E-06	Var9	3.2E-05
16	Var55	6.75E-06	3.1E-06	Var44	3.14E-05
17	Var28	5.95E-06	7.61E-05	Var43	2.92E-05
18	Var3	5.24E-06	3.76E-06	Var18	1.91E-05
19	Var31	3.25E-06	3.69E-06	Var26	1.11E-05
20	Var2	2.67E-06	1.52E-06	Var51	9.48E-06
21	Var77	1.7E-06	1.09E-06	Var31	8E-06
22	Var6	1.6E-06	7.28E-07	Var55	7.87E-06
23	Var19	1.35E-06	1.95E-06	Var27	7.77E-06
24	Var39	1.33E-06	1.69E-06	Var5	6.27E-06
25	Var47	1.26E-06	5.15E-07	Var68	6.19E-06

(a) With bootstrapping

No.	Random permutation			SHAP	
	Variable	Mean	Standard deviation	Variable	Score
1	Var48	0.000114	0.000107	Var46	0.000466
2	Var53	5.12E-05	7.37E-05	Var48	0.000443
3	Var52	4.39E-05	8.14E-05	Var50	0.000386
4	Var5	3.74E-05	2.65E-05	Var52	0.000324
5	Var46	3.48E-05	9.01E-05	Var53	0.000311
6	Var50	3.3E-05	6.75E-05	Var51	0.000213
7	Var8	3.14E-05	2.32E-05	Var8	0.000102
8	Var49	2.93E-05	2.01E-05	Var5	8.54E-05
9	Var2	2.25E-05	1E-05	Var57	7.67E-05
10	Var42	1.4E-05	7.07E-06	Var49	7.39E-05
11	Var76	1.03E-05	6.16E-06	Var55	6.21E-05
12	Var57	9.46E-06	1.7E-05	Var43	6.21E-05
13	Var31	7.16E-06	2.51E-06	Var2	4.79E-05
14	Var10	4.71E-06	2.8E-06	Var56	4.22E-05
15	Var21	4.53E-06	3.02E-06	Var42	3.68E-05
16	Var7	3.94E-06	2.07E-06	Var18	3.35E-05
17	Var45	3.48E-06	1.03E-06	Var58	3.19E-05
18	Var34	3.43E-06	2.68E-06	Var36	2.77E-05
19	Var47	3.29E-06	1.04E-06	Var11	2.73E-05
20	Var27	2.98E-06	1.84E-06	Var4	2.70E-05
21	Var54	2.97E-06	1.07E-06	Var37	2.55E-05
22	Var15	2.67E-06	1.86E-06	Var76	2.33E-05
23	Var23	2.65E-06	2.21E-06	Var29	2.14E-05
24	Var38	2.61E-06	1.23E-06	Var39	1.97E-05
25	Var65	2.41E-06	1.34E-06	Var21	1.32E-05

(b) Without bootstrapping
