# Supplementary material for: Investigation of alpha-glucosidase inhibition activity of Artabotrys sumatranus leaf extract using metabolomics, machine learning and molecular docking analysis
Source: PLoS One. 2025 Jan 3;20(1):e0313592. doi: 10.1371/journal.pone.0313592 (PMC11698457; doi:10.1371/journal.pone.0313592)
Supplement: S5 Table — The entries in the table show the amino acids of 3A4A receptor which were involved in the bonding. The amino acids are arranged so that same kind of interactions which appeared in other identified predicted active compounds can be recognized. (PDF) [file pone.0313592.s005.pdf]

S5 Table. Molecular interactions between identified predicted active compounds and 3A4A receptor. The entries in the table show the amino acids of 3A4A receptor which were involved in the bonding. The amino acids are arranged so that same kind of interactions which appeared in other identified predicted active compounds can be recognized.

Type of Bonding	Acarbose	Mangi-ferin	Neo-mangi-ferin	15,16-Dihydro-tanshino-ne I	Lirioferin	Noriso-corydine	Apigenin -7-O-Galacto-pyrano-side
Hydrogen bonding	Glu A:411	Glu A:411					
	Gln A:279		Gln A:279				
			Ser A:157				Ser A:157
	Tyr A:158		Tyr A:158		Tyr A:158		
	Asp A:215		Asp A:215			Asp A:215	
	Arg A:442		Arg A:442				
					Gln A:353	Gln A:353	
					Asp A:352	Asp A:352	
						Asp:307	
	Asp A:69						Asp A:69
	Arg A:315						
	His A: 351						
	His A:112						
Total hydrogen bonding	9	1	5	0	3	4	2
Unfavorable bump			Glu A:277				
			Asp A:69				
Total unfavorable bump	0	0	2	0	0	0	0
Van der Waals		Asp A:69			Asp A:69		
		Tyr A:79					

Type of Bonding	Acarbose	Mangi-ferin	Neo-mangi-ferin	15,16-Dihydro-tanshino-ne I	Lirioferin	Noriso-corydine	Apigenin-7-O-Galacto-pyrano-side
		Asp A:215			Asp A:215		Asp A:215
		His A:351	His A:351				
	Arg A:213	Arg A:213	Arg A:213			Arg A:213	
		Gln A:279				Gln A:279	
	Phe A:303	Phe A:303	Phe A:303				Phe A:303
		His A:280	His A:280	His A:280			
		Ser A:240	Ser A:240				
		Lys A: 156	Lys A: 156				Lys A: 156
		Phe A: 314	Phe A: 314	Phe A: 314			Phe A: 314
	Asn A:415	Asn A:415					Asn A:415
	Tyr A: 316	Tyr A: 316	Tyr A: 316				
		Arg A:319					
			Asp A:307	Asp A:307			
			Gly A: 160				Gly A: 160
			Asn A:415				
			Phe A: 159	Phe A: 159	Phe A: 159	Phe A: 159	Phe A: 159
			Glu A:411				
			His A:112				His A:112
			Gln A: 182				Gln A: 182
			Arg A:446				
			Met A:70				

Type of Bonding	Acarbose	Mangi-ferin	Neo-mangi-ferin	15,16-Dihydro-tanshino-ne I	Lirioferin	Noriso-corydine	Apigenin-7-O-Galacto-pyrano-side
				Tyr A:158			
				Gln A:353			Gln A:353
				Asp A: 352			Asp A: 352
				Arg A:442			
					Asn A:350		
					Thr A: 306		
					Glu: 277		Glu A:277
					Val A: 216	Val A: 216	
	Phe A:178				Phe A:178	Phe A:178	
					Arg A:315	Arg A:315	Arg A:315
	Ser A:157						
	Tyr A:72						
Total Van der Waals	7	14	19	8	8	6	17
Carbon hydrogen bond		Tyr A: 158					
		Phe A:159					
		Phe A:178					
					Glu A:411		
					Tyr A:158		
					Gln A:353		
	Asp A:352		Asp A:352		Asp A:352		
	Phe A:314						

Type of Bonding	Acarbose	Mangi-ferin	Neo-mangi-ferin	15,16-Dihydro-tanshino-ne I	Lirioferin	Noriso-corydine	Apigenin -7-O-Galacto-pyrano-side
							Tyr A:158
	Glu A: 277						
Total carbon hydrogen bond	3	3	1	0	4	0	1
Unfavorable acceptor-acceptor		Glu A: 277					
	Asp A:215						
Total unfavorable acceptor-acceptor	1	1	0	0	0	0	0
Unfavorable donor-donor			Arg A:315				
			Tyr:72				
Total Unfavorable donor-donor	0	0	2	0	0	0	0
Pi cation		Asp A: 352			Asp A: 352	Asp A: 352	
					Glu A:411	Glu A:411	Glu A:411
Total pi cation	0	1	0	0	2	2	1
Pi anion		Arg A:442			Arg A:442	Arg A:442	Arg A:442
				Glu A: 411			
Total pi anion	0	1	0	1	1	1	1
Pi sigma					Phe A:303		
					Tyr A:72		
Total pi sigma	0	0	0	0	2	0	0
Pi-pi stacked				Phe A:303			

Type of Bonding	Acarbose	Mangi-ferin	Neo-mangi-ferin	15,16-Dihydro-tanshino-ne I	Lirioferin	Noriso-corydine	Apigenin -7-O-Galacto-pyrano-side
							Tyr A:72
Total pi-pi stacked	0	0	0	1	0	0	1
Pi-alkyl		Val A:216	Val A:216				Val A:216
				Arg A:315			
					His A:351	His A:351	
						Phe A:303	
						Tyr A:72	
Total pi-alkyl	0	1	1	1	1	3	0
Total favorable bonds	19	21	26	11	21	16	23
Total unfavorable bonds	1	1	4	0	0	0	0
