# Supplementary material for: Investigation of alpha-glucosidase inhibition activity of Artabotrys sumatranus leaf extract using metabolomics, machine learning and molecular docking analysis
Source: PLoS One. 2025 Jan 3;20(1):e0313592. doi: 10.1371/journal.pone.0313592 (PMC11698457; doi:10.1371/journal.pone.0313592)
Supplement: S6 Table — The entries in the table show the amino acids of 3TOP receptor which were involved in the bonding. The amino acids are arranged so that same kind of interactions which appeared in other identified predicted active compounds can be recognized. (PDF) [file pone.0313592.s006.pdf]

S6 Table. Molecular interactions between identified predicted active compounds and 3TOP receptor. The entries in the table show the amino acids of 3TOP receptor which were involved in the bonding. The amino acids are arranged so that same kind of interactions which appeared in other identified predicted active compounds can be recognized.

Type of bonding	Acarbose	Mangi-ferin	Neo-mangi-ferin	15,16-Dihydro-tanshino-ne I	Lirioferin	Noriso-corydine	Apigenin -7-O-Galacto-pyrano-side
Hydrogen bonding		Lys A: 509			Lys A: 509		
							Gln A: 335
	Arg A: 559	Arg A: 559					Arg A: 426
		Asp A: 469					
	Asp A: 328	Asp A: 328					
		His A: 633					
			Asp A: 206				
Total hydrogen bonding	2	5	1	0	1	0	2
Van der Waals		Asp A: 206					
		Pro A: 208			Pro A: 208	Pro A: 208	
	Phe A: 476	Phe A: 476	Phe A : 476				
		Trp A: 418					
	Thr A: 635	Thr A: 635	Thr A: 635	Thr A: 635	Thr A: 635	Thr A: 635	
		Ile A: 364	Ile A: 364	Ile A: 364			
	Trp A: 467	Trp A: 467					
	Trp A: 572	Trp A: 572	Trp A: 572				
			Thr A: 638				
			Gln A: 610				

Type of bonding	Acarbose	Mangi-ferin	Neo-mangi-ferin	15,16-Dihydro-tanshino-ne I	Lirioferin	Noriso-corydine	Apigenin-7-O-Galacto-pyrano-side
			Arg A: 559	Arg A: 559	Arg A: 559	Arg A: 559	Arg A: 559
			Asp A: 469	Asp A: 469			
			Trp A: 467				
			His A: 633				
			Ile A: 329	Ile A: 329			
			Tyr A: 300		Tyr A: 300		Tyr A: 300
			Phe A: 608		Phe A: 608	Phe A: 608	Phe A: 608
			Met A: 470	Met A: 470	Met A: 470	Met A: 470	Met A: 470
			Tyr A: 365				
			Ser A: 474				
				Ile A: 636	Ile A: 636	Ile A: 636	
				Trp A: 404			
				Asp A: 328			
					Gln A:207		
					Asp A: 575		
							Asp A: 406
							Asn A: 413
							Gly A: 414
							Gln A: 421
							Tyr A: 425
							Val A: 412

Type of bonding	Acarbose	Mangi-ferin	Neo-mangi-ferin	15,16-Dihydro-tanshino-ne I	Lirioferin	Noriso-corydine	Apigenin-7-O-Galacto-pyrano-side
						Lys A: 509	
						Asp A: 206	
	Phe A: 609						
	Trp: 404						
	Met A: 470						
	Phe A: 608						
	Ile A:329						
	Tyr A: 300						
	Ile A: 364						
	His A: 633						
	Asp A: 469						
Total Van der Waals	13	8	16	9	9	8	10
Carbon hydrogen bond				Asp A:206	Asp A:206		Trp A: 418
				Asp A: 575			
						Asp A: 328	
Total carbon hydrogen bond	0	0	0	2	1	1	1
Unfavor-able acceptor-acceptor			Gly A: 637				Asp A: 575
Total unfavor-able acceptor-acceptor	0	0	1	0	0	0	1

Type of bonding	Acarbose	Mangi-ferin	Neo-mangi-ferin	15,16-Dihydro-tanshino-ne I	Lirioferin	Noriso-corydine	Apigenin-7-O-Galacto-pyrano-side
Unfavor-able donor-donor			Ile A: 636				
Total unfavor-able donor-donor	0	0	1	0	0	0	0
Pi anion		Met A: 373					
			Asp A: 575			Asp A: 575	
Total pi anion	0	1	1	0	0	1	0
Pi sigma					Trp A:418	Trp A:418	
Total pi sigma	0	0	0	0	1	1	0
Pi-pi T-Shaped		Trp A: 404	Trp A: 404		Trp A: 404	Trp A: 404	Trp A: 404
		Phe A: 609	Phe A: 609	Phe A:609	Phe A:609	Phe A:609	Phe A:609
		Tyr A: 300		Tyr A: 300			
		Phe A: 608		Phe A: 608			
Total pi-pi T-shaped	0	4	2	3	2	2	2
Pi-alkyl				Pro A: 208			Ile A: 636
				His A: 633			
				Trp A: 467			
				Trp A: 572			
						Trp A: 418	
Total pi-alkyl	0	0	0	4	0	1	0

Type of bonding	Acarbose	Mangi-ferin	Neo-mangi-ferin	15,16-Dihydro-tanshino-ne I	Lirioferin	Noriso-corydine	Apigenin -7-O-Galacto-pyrano-side
Salt Bridge	Asp A: 206						
Total salt bridge	1	0	0	0	0	0	0
Attractive Charge	Asp A: 575						
Total attractive charge	1	0	0	0	0	0	0
Total favorable bonds	17	18	20	18	14	14	15
Total unfavor-able bonds	0	0	2	0	0	0	1
