# Supplementary material for: Investigation of alpha-glucosidase inhibition activity of Artabotrys sumatranus leaf extract using metabolomics, machine learning and molecular docking analysis
Source: PLoS One. 2025 Jan 3;20(1):e0313592. doi: 10.1371/journal.pone.0313592 (PMC11698457; doi:10.1371/journal.pone.0313592)

S1 Figure. The workflow of the isolation of active compound (mangiferin) from *Artabotrys sumatranus* leaf extract using bioassay guided fractionation. The amount and α-glucosidase inhibition bioassay results for the isolation fractions are shown in this figure.

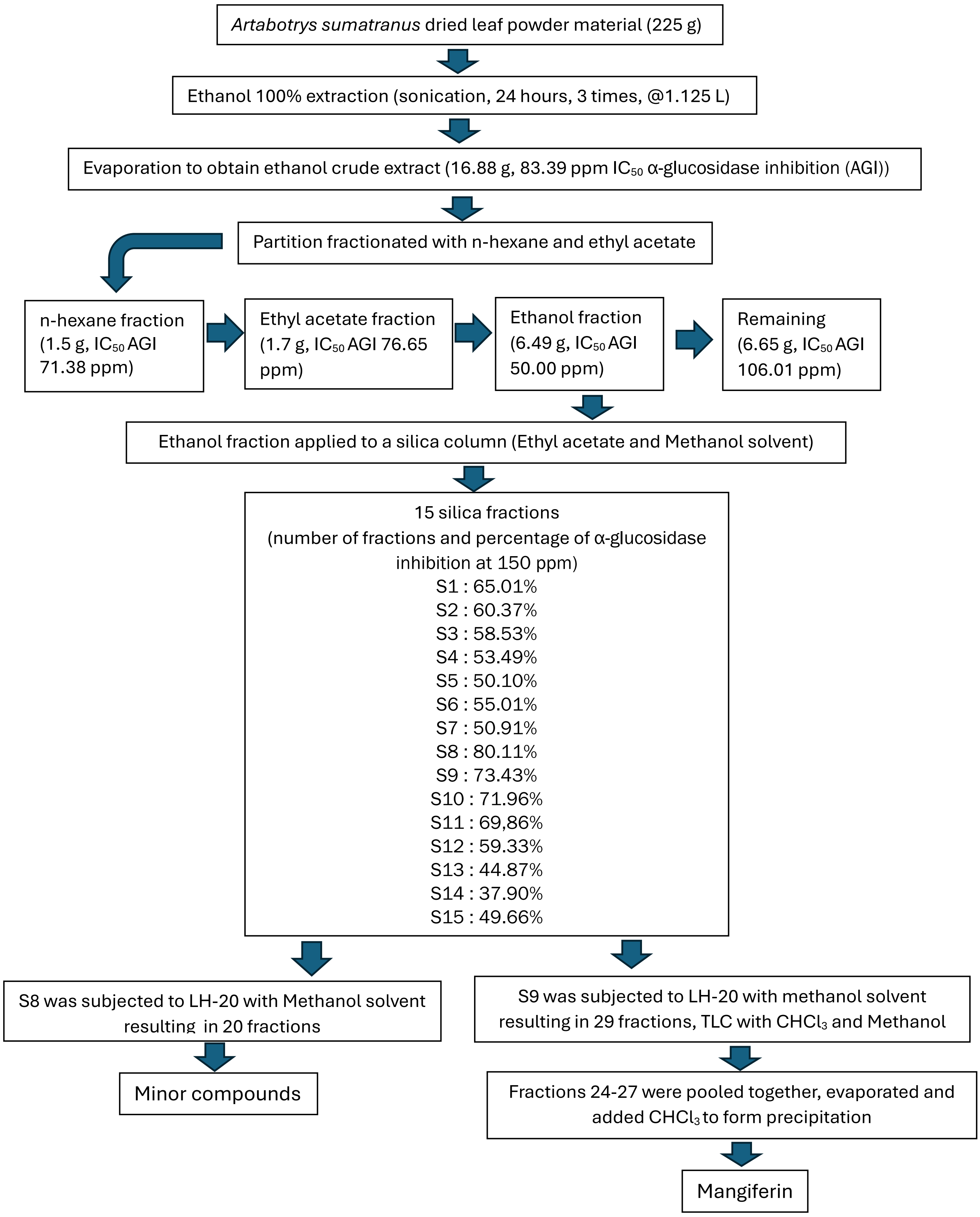

Supplement: S1 Fig — The amount and α-glucosidase inhibition bioassay results for the isolation fractions are shown in this figure. (PDF) [file pone.0313592.s007.pdf]
