# Supplementary material for: Investigation of alpha-glucosidase inhibition activity of Artabotrys sumatranus leaf extract using metabolomics, machine learning and molecular docking analysis
Source: PLoS One. 2025 Jan 3;20(1):e0313592. doi: 10.1371/journal.pone.0313592 (PMC11698457; doi:10.1371/journal.pone.0313592)

S1 File. Analysis of fragmentation patterns of the predicted active compound using LC-MS/MS.

This file contains the analysis which has been done on the fragmentation patterns of the compounds predicted to be active by the metabolomic analysis. The metabolomic analysis predicted the active compounds in the form of m/z values. To identify the predicted active compounds, LC-MS/MS in positive mode analysis was done to get the further fragmentation patterns of the m/z values of these predicted active compounds. First the fragmentation patterns were compared automatically to the available database of the LC-MS/MS machine, but to validate these automatic identifications, a manual analysis of the fragmentation patterns was done. The result of the manual analysis confirmed the automatic identification.

The result of this manual analysis was presented here in this format:

- First, the fragmentation patterns for the predicted active compound were shown. The LC-MS/MS was set to positive mode for this analysis.
- Second, an analysis was made to predict what kind of fragmentations could happen to produce the observed fragmentation patterns. The analysis results were shown as transition paths of possible fragmentations of the predicted active compounds. The fragmentations were labelled with the corresponding m/z value.

In this file, the analysis results were given for the following predicted active compounds:

a. Mangiferin with m/z value of 423.093 (variable 45 in metabolomic analysis)
b. Neomangiferin with m/z value of 585.145 (variable 31 in metabolomic analysis)
c. 15,16-Dihiydrotanshinone with m/z value of 279.232 (variable 46 in metabolomic analysis)
d. Lirioferine with m/z value of 342.171 (variable 43 in metabolomic analysis)
e. Norisocorydine with m/z value of 328.155 (variable 44 in metabolomic analysis)
f. Apigenin-7-o-galactopyrasinoside with m/z value 433.114 (variable 47 in metabolomic analysis)

1. Mangiferin fragmentation analysis, showing m/z from LC-MS/MS analysis and compound fragmentation prediction to get the m/z profile detected

Mangiferin

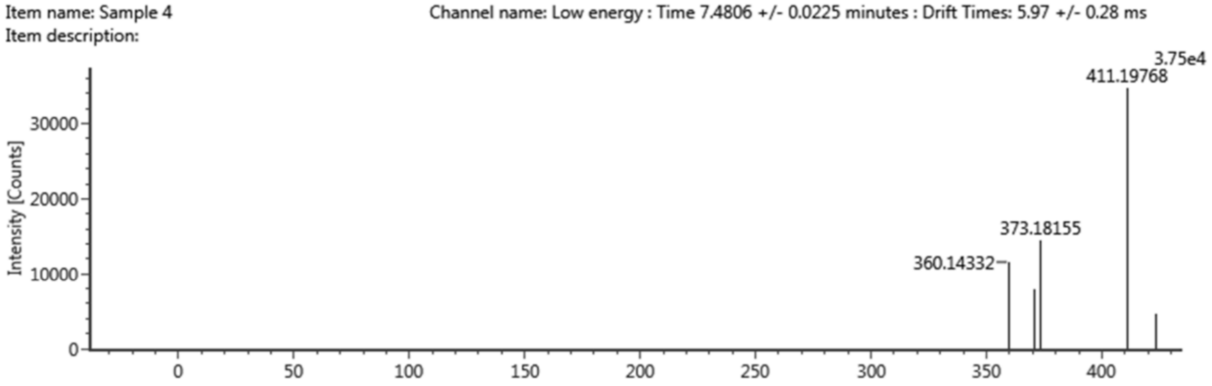

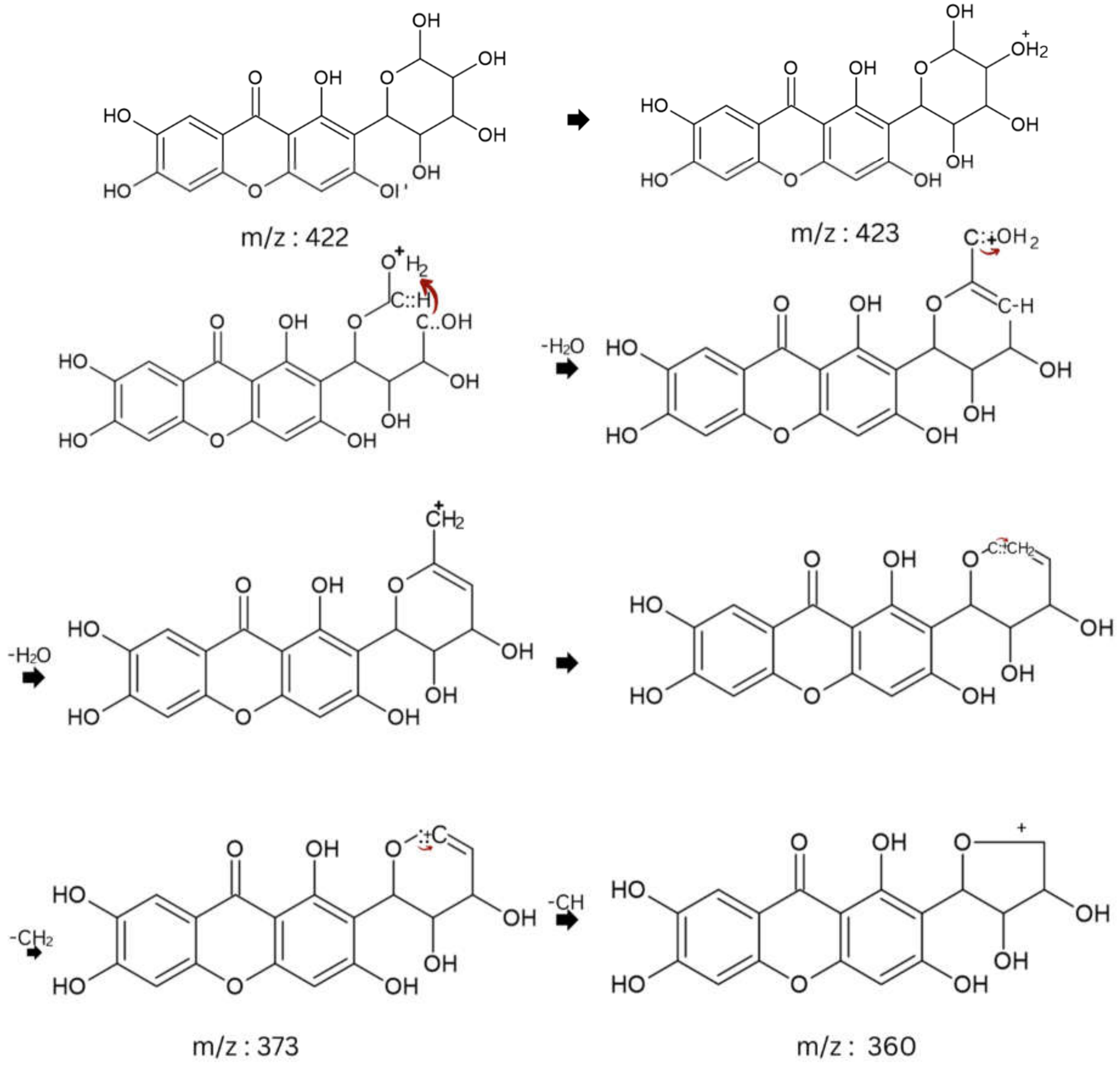

2. Neomangiferin fragmentation analysis, showing m/z from LC-MS/MS analysis and compound fragmentation prediction to get the m/z profile detected

Neomangiferin

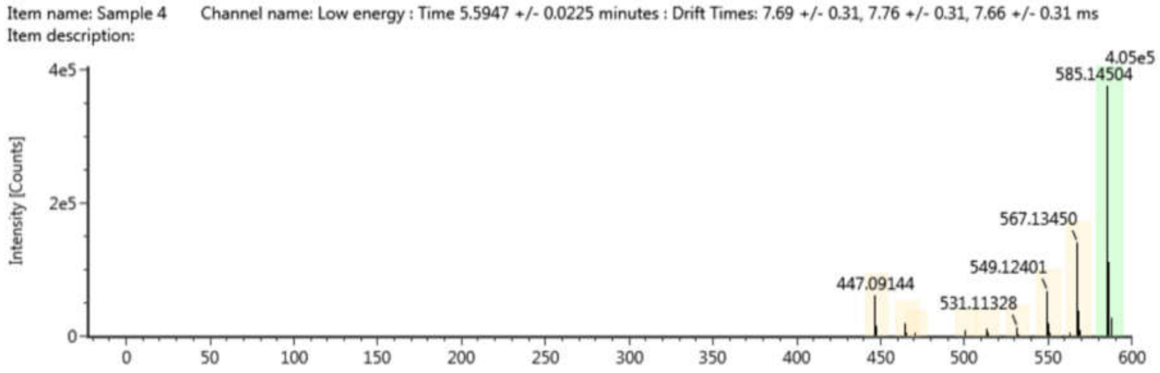

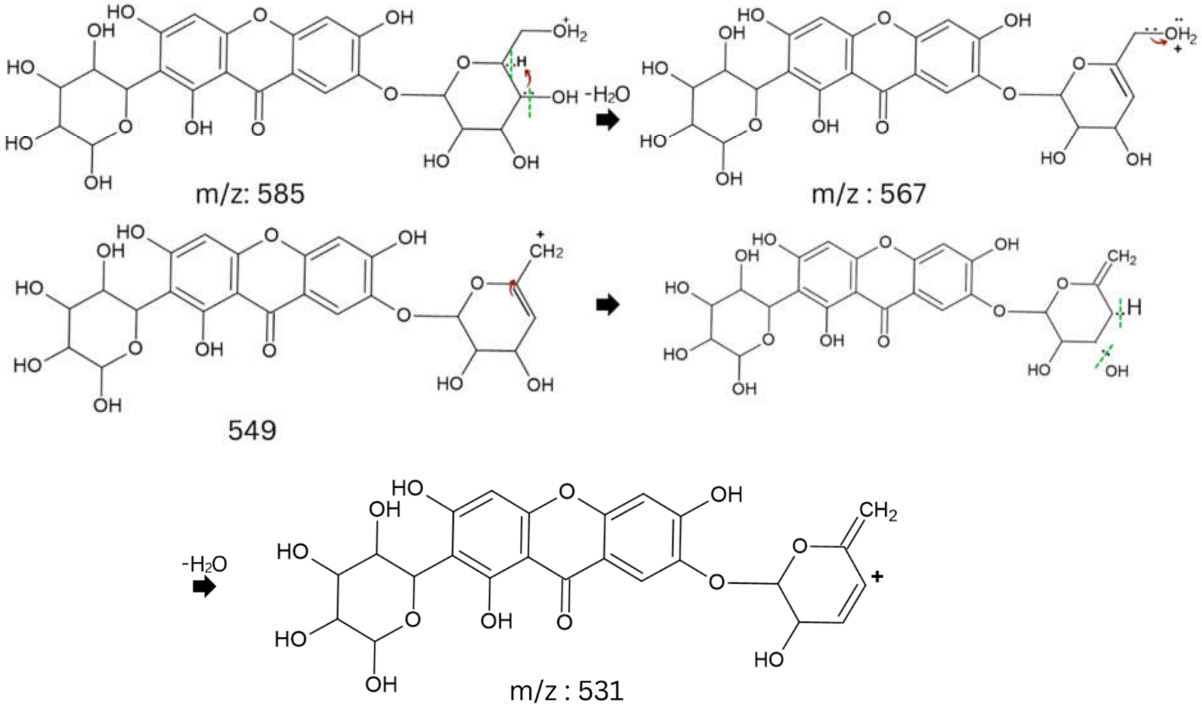

3. 15,16-Dihydrotanshinone fragmentation analysis, showing m/z from LC-MS/MS analysis and compound fragmentation prediction to get the m/z profile detected

15,16 dihydrotanshinone

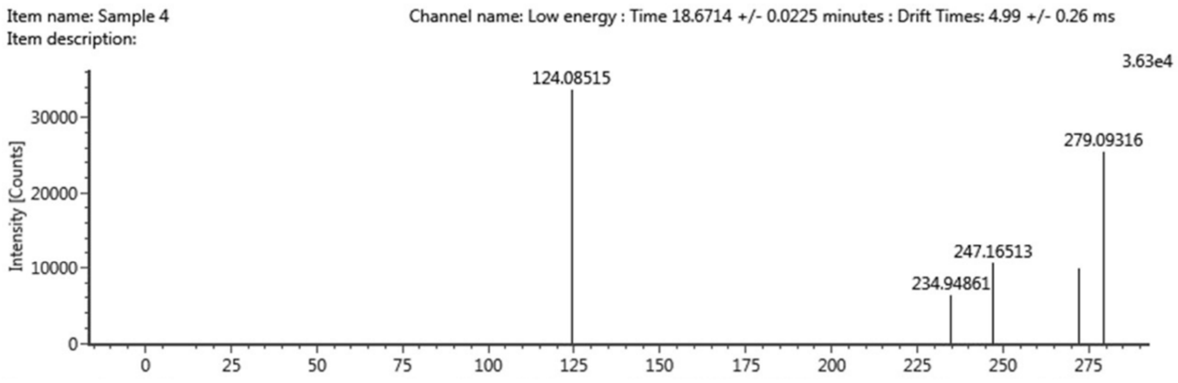

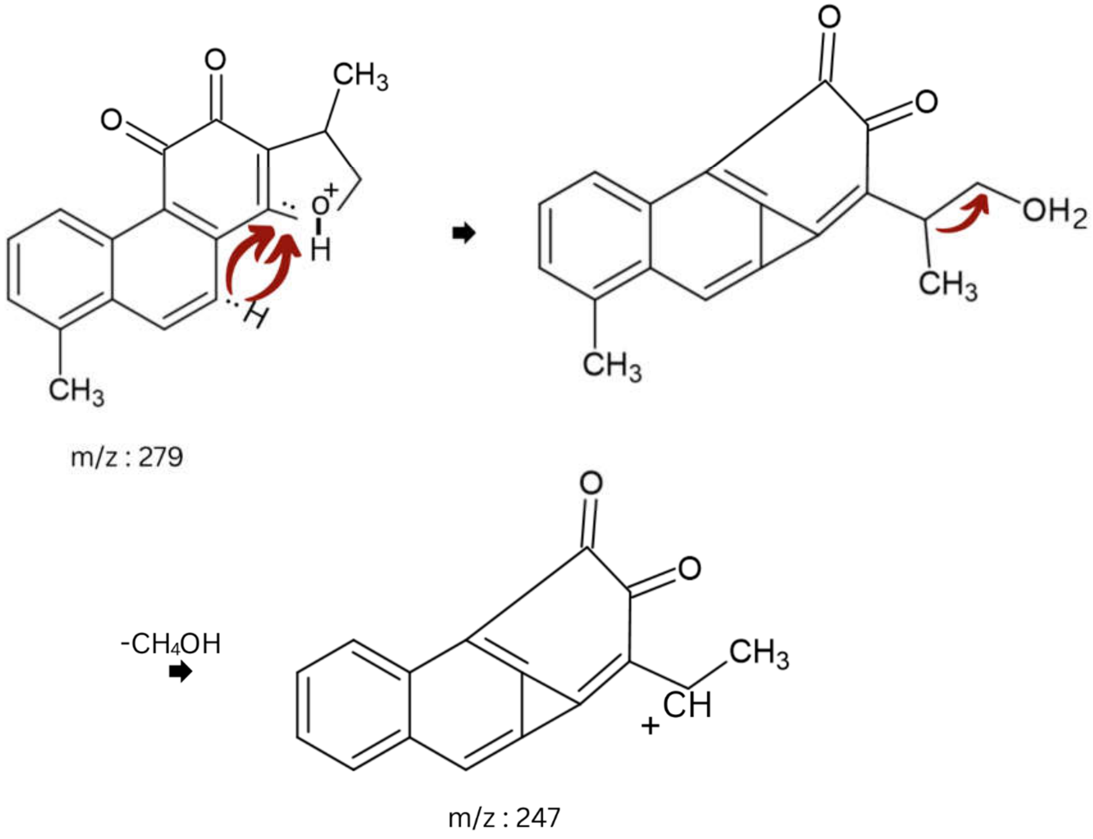

15,16 dihydrotanshinone

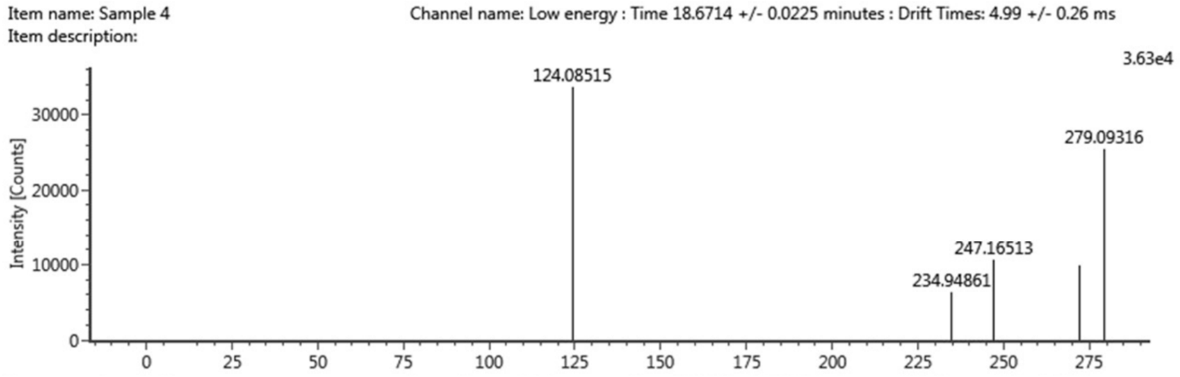

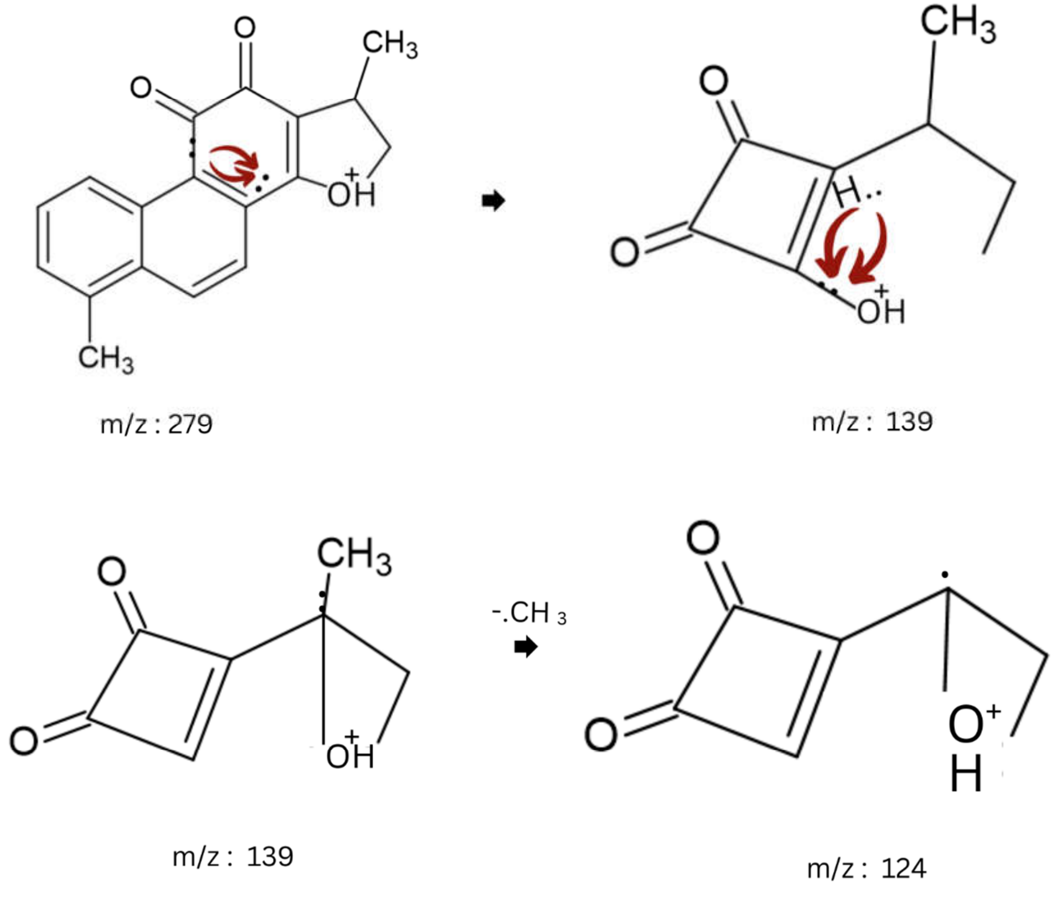

15,16 dihydrotanshinone

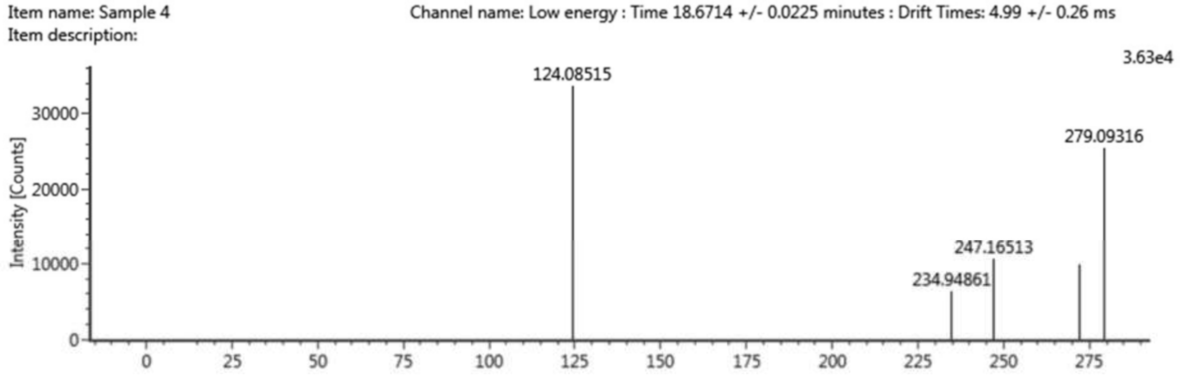

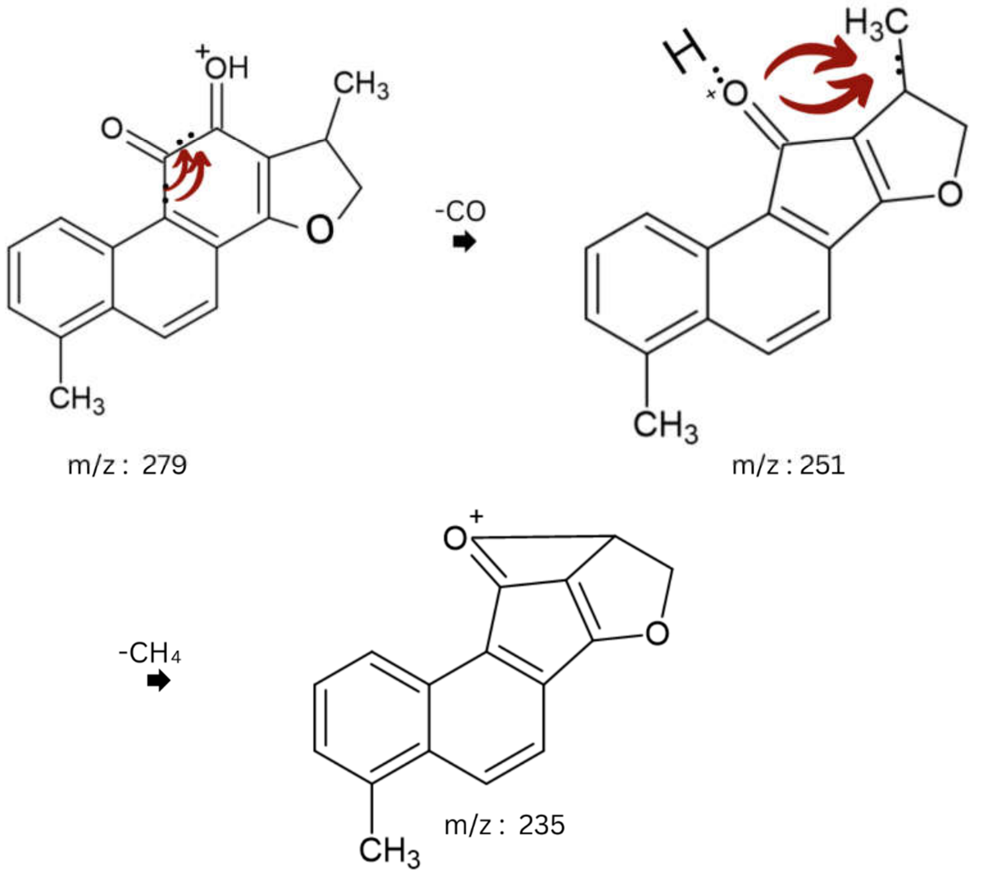

4. Lirioferine fragmentation analysis, showing m/z from LC-MS/MS analysis and compound fragmentation prediction to get the m/z profile detected

Lirioferine

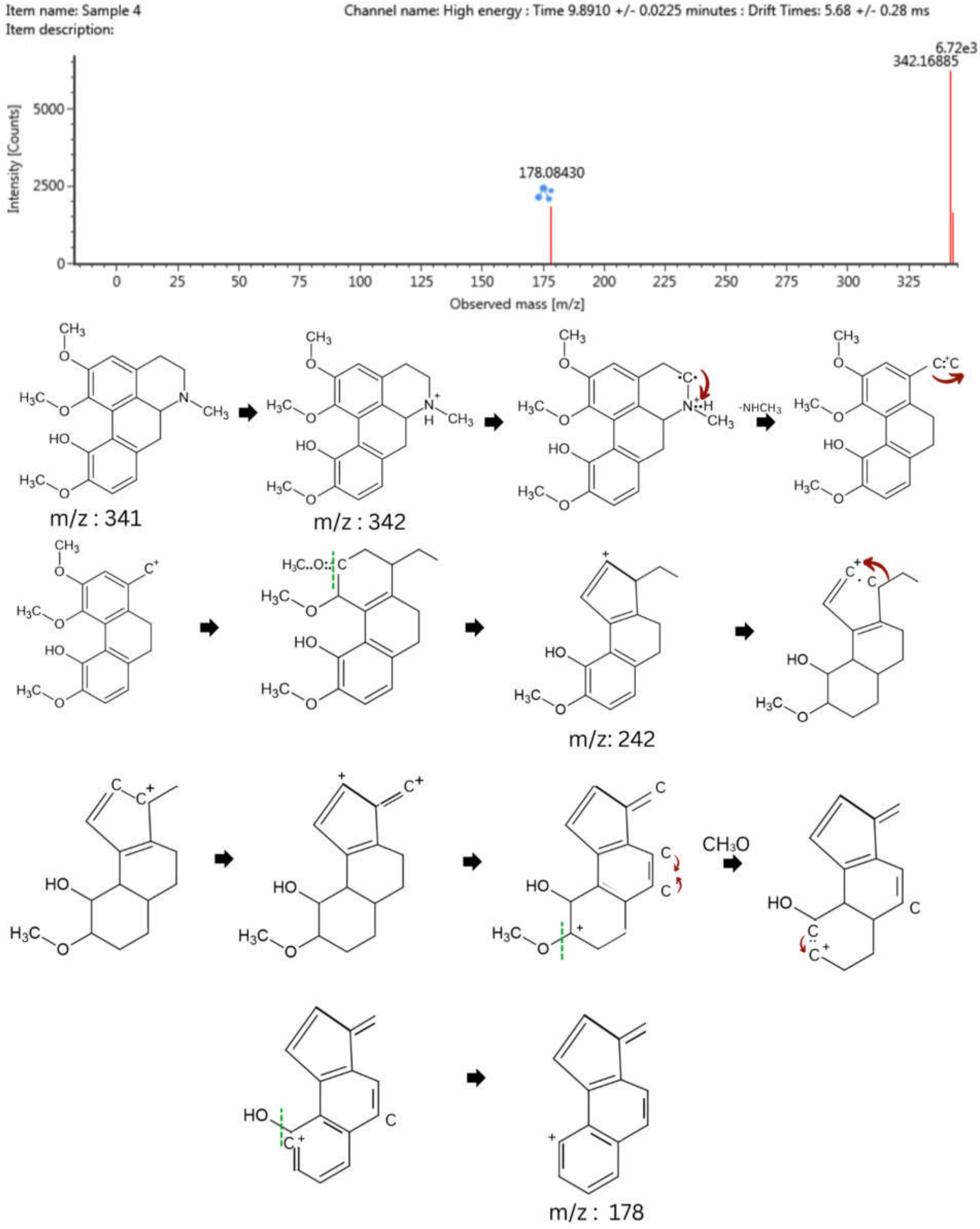

5. Norisocorydine fragmentation analysis, showing m/z from LC-MS/MS analysis and compound fragmentation prediction to get the m/z profile detected

Norisocorydine

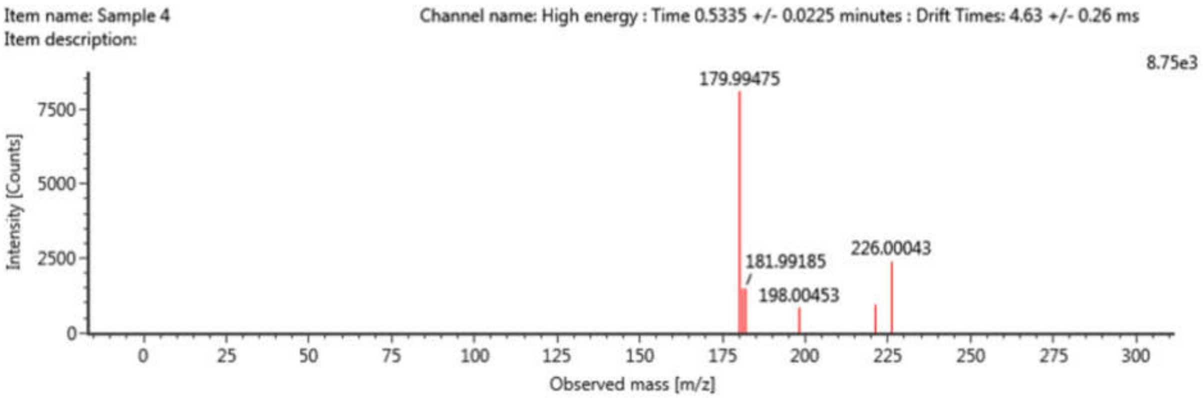

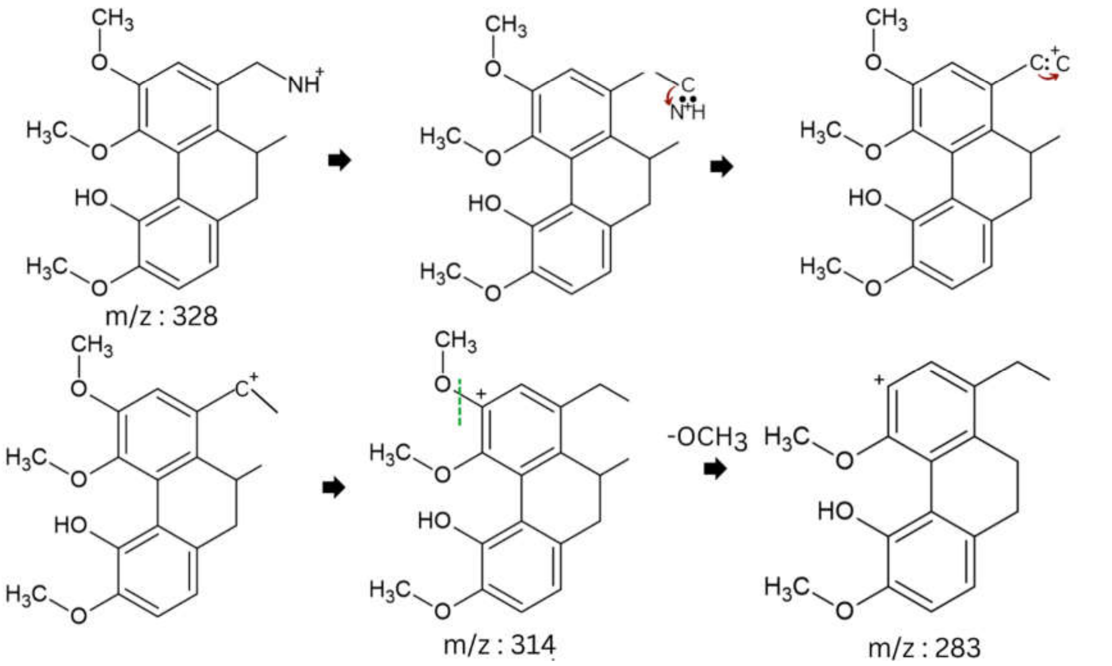

Norisocorydine

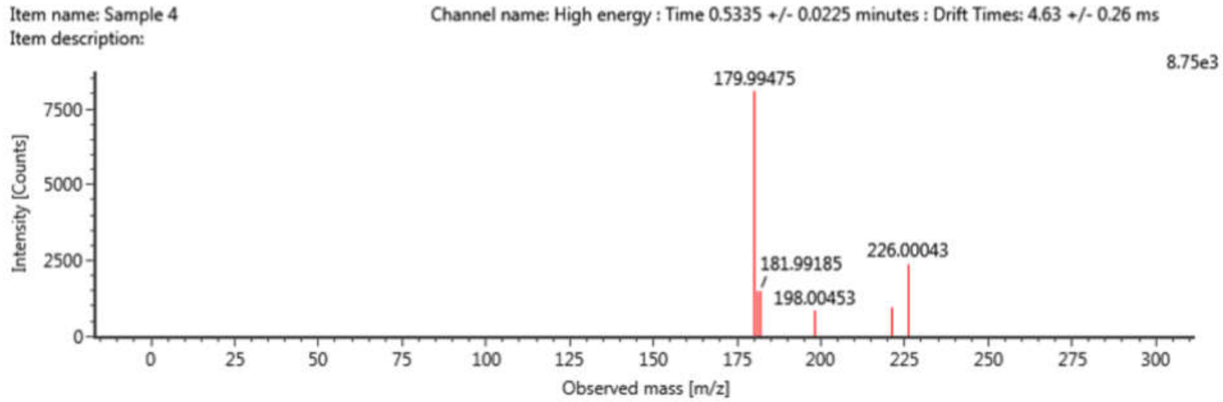

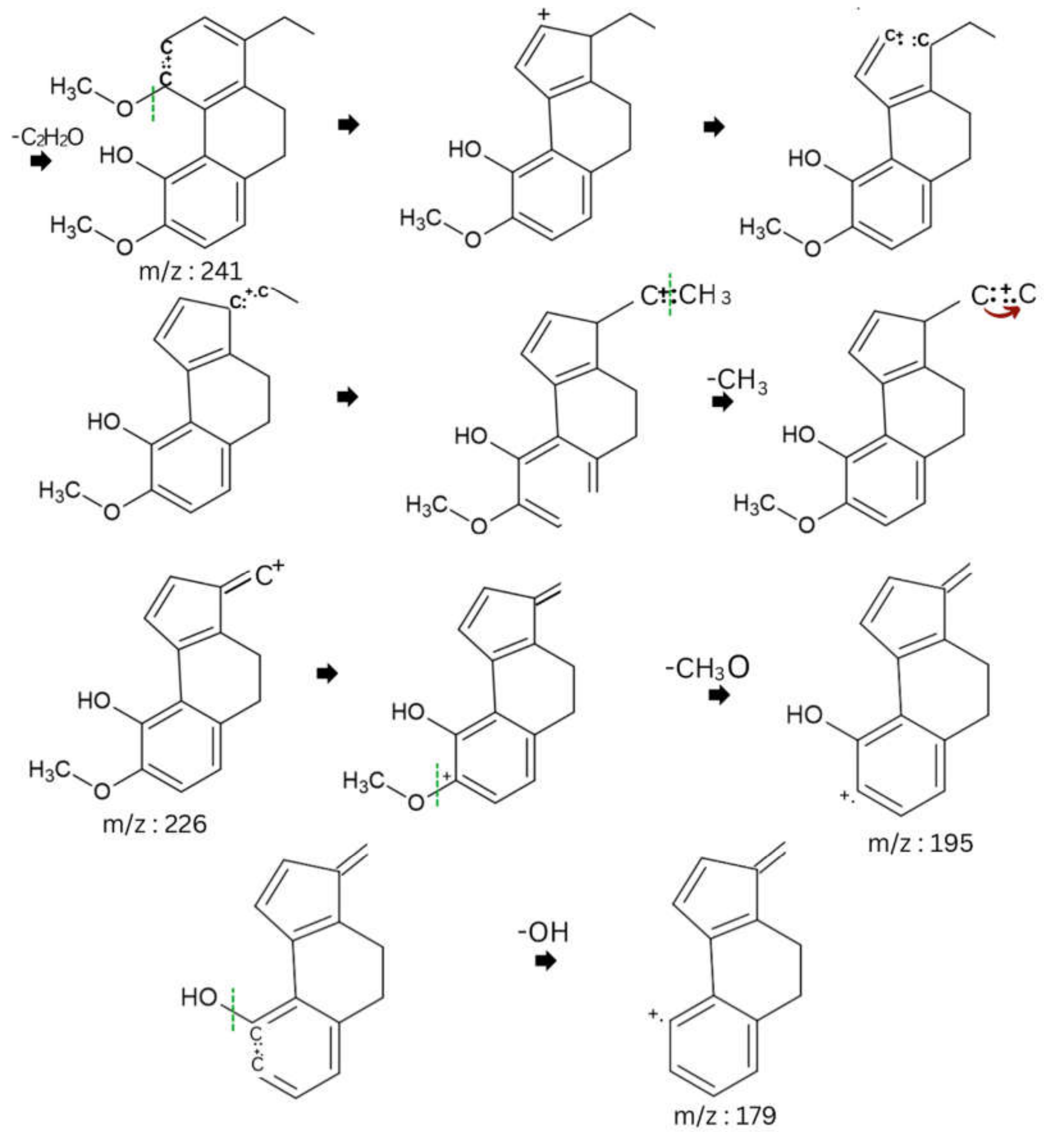

6. Apigenin-7-o-galactopyrasinoside fragmentation analysis, showing m/z from LC-MS/MS analysis and compound fragmentation prediction to get the m/z profile detected

Apigenin -7-O-glucopyrasinoside

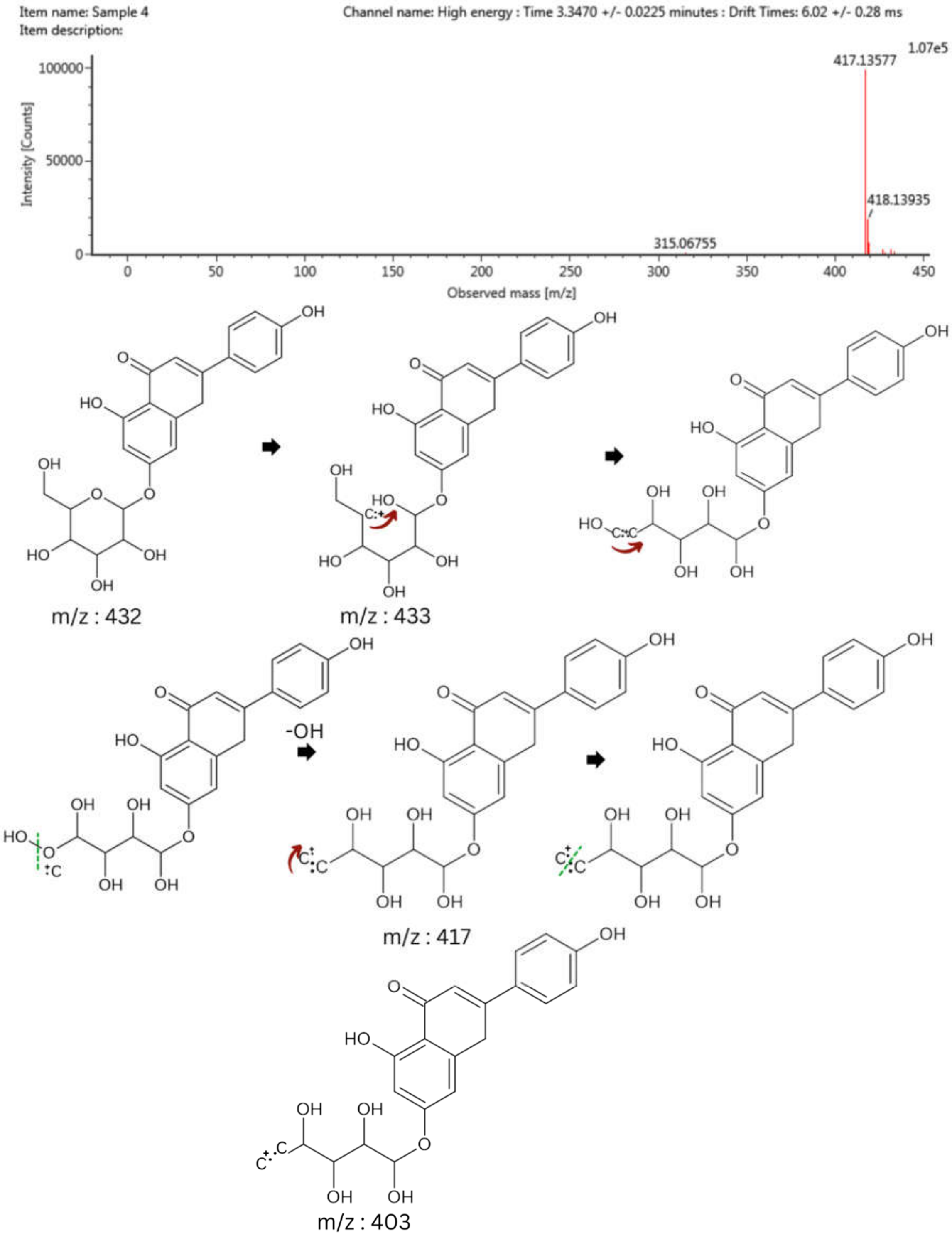

Supplement: S1 File — In this file the analysis of the fragmentation patterns is presented for m/z values of the compounds which were predicted to be active by the metabolomics analysis. The fragmentation patterns were obtained from LC-MS/MS. The analysis results verify the identification of the active compounds. (PDF) [file pone.0313592.s008.pdf]
