# Supplementary material for: Investigation of alpha-glucosidase inhibition activity of Artabotrys sumatranus leaf extract using metabolomics, machine learning and molecular docking analysis
Source: PLoS One. 2025 Jan 3;20(1):e0313592. doi: 10.1371/journal.pone.0313592 (PMC11698457; doi:10.1371/journal.pone.0313592)
Supplement: S2 File — This file contains the results of 1D NMR (1H NMR and 13C NMR) and 2D NMR (HMBC (heteronuclear multiple bond correlation) and HSQC (heteronuclear single quantum coherence)) of the isolated compound from Artabotrys sumatranus leaf extract. The elucidation analysis is also explained. (PDF) [file pone.0313592.s009.pdf]

S2 File. Elucidation of mangiferin as isolated compound from *Artabotrys sumatranus* leaf extract

This file contains the results of 1D NMR (^{1}H NMR and ^{13}C NMR) and 2D NMR (HMBC (heteronuclear multiple bond correlation) and HSQC (heteronuclear single quantum coherence)) of the isolated compound from *Artabotrys sumatranus* leaf extract. The elucidation analysis is also explained.

The structure of the isolated compound was shown in

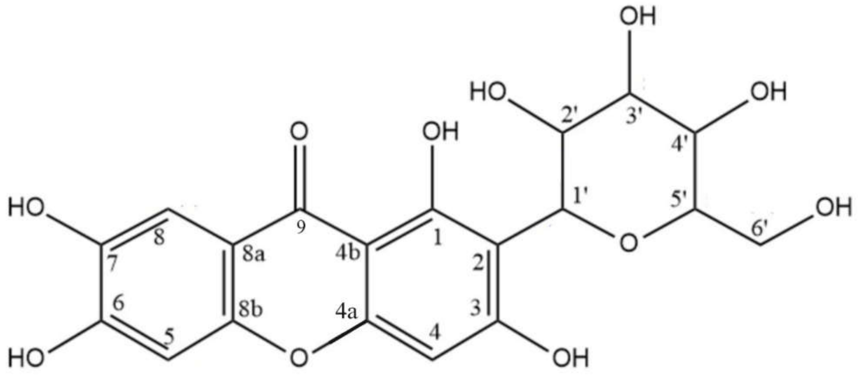

Fig S1-1. Structure of mangiferin, isolated compound from *Artabotrys sumatranus* leaf extract. The numbers identified the carbon and hydrogen atoms, which were used in the elucidation explanation.

This structure in Fig S1-1 was elucidated from NMR results in part A, B, C, and D of this appendix. From these NMR results, especially the 2D NMR, the connection and correlation between the C and H atoms could be deduced. The correlation and connection could be seen in Fig S1-2.

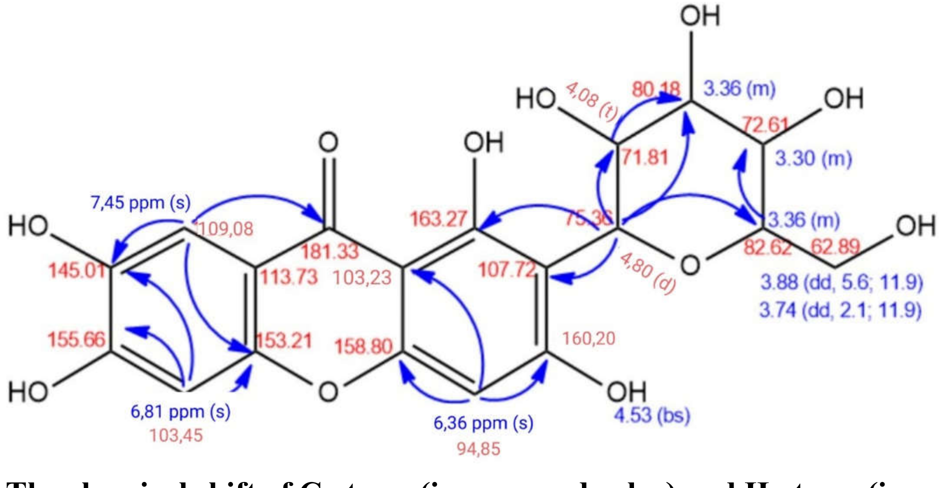

Fig S1-2. The chemical shift of C atoms (in ppm, red color) and H atoms (in ppm, blue color), as well as the correlations between the C and H atoms (blue arrows), of mangiferin, the isolated compound. The types of signals are also shown: s = singlet, bs = broad singlet, d = doublet, t = triplet, dd = double doublet, m = multiplet.

Results of ^{1}H NMR showed that there were signals in two regions: aliphatic and aromatic. Signals in aliphatic regions had chemical shift (δ) between 3.3 ppm until 4.9 ppm. The isolate showed many signals in the aliphatic regions, indicating the presence of sugar moeities in the isolate. HSQC showed connection between hydrogen atom H-1' (δ 4.80 ppm) with carbon atom C-1' (δ 75.36 ppm), indicating a C-C bond between sugar and aglicon moeities.

Signals in aromatic region had chemical shift δ between 6-8 ppm. In ^{1}H NMR result, the isolate showed 3 singlets at δ 6.36 ppm, 6.81 ppm, and 7.45 ppm. Singlet indicated that there was a hydrogen atom without neighboring hydrogen atoms within 3 bonding distance. Therefore, it could be concluded that these 3 singlets corresponded to atoms H-4, H-5, and H-8 in Fig S1-1.

From ^{13}C NMR results, it could be seen that there was a signal at δ 181.33 ppm which indicated the existence of a carbon atom which was bonded to keton moeity and khelated to hydroxyl moeity. This signal seemed to correspond to carbon atom number 9 (C-9).

Direct bonds between hydrogen and carbon atoms could be seen from HSQC results: hydrogen atoms in aromatic regions with 6.36 ppm (H-4), δ 6,81 ppm (H-5), and δ 7.45 ppm (H-8) directly bonded with the carbon atoms with δ 94.85 ppm (C-4), δ 103.45 ppm (C-5), and δ 109.08 ppm (C-8) respectively. From HMBC results, it could be seen that hydrogen atom with δ 6.36 ppm (H-4) resonated with 5 carbon atoms with δ 103.23 ppm (C-4b), 158.80 ppm (C-4a), 165.20 ppm (C-3), 107.72 ppm (C-2), dan 181.33 ppm (C-9). By analyzing the neighborhood of each carbon atom which might resonate with hydrogen atom H-4, it could be concluded that carbon atom with δ 103.23 ppm was atom C-4b, since this carbon atom was the best protected compared to other carbon atoms, and also because this carbon atom resonated with hydrogen atom H-8. Then the atom carbon with δ 107.72 ppm was atom C-2, because the electron distribution was more pulled towards sugar moeity making atom C-2 less protected compared to atom C-4b. Carbon atom with δ 158.80 ppm was deduced to be atom C-4a, since this atom C-4a was less protected than atom C-9a. This was due to direct bond of this atom carbon to oxygen atom, which pulled the electron distribution more towards the more electronegative oxygen atom. Carbon atom with δ 165.20 ppm was atom C-3 which was least protected, because this atom was directly bonded with hydroxyl moeity which had large electronegativity. The three hydrogen atoms (H-4, H-5, and H-8) resonated with carbon atom C-9, which had keton moeity with δ 181.33 ppm. Similar to H-4, hydrogen atom H-8 also resonated with 5 carbon atoms, which had δ 103.23 ppm (C-4b), δ 145.01 ppm, δ 153.21 ppm, δ 155.66 ppm, and δ 181.33 ppm (C-9). The three carbon atoms with δ 145.01 ppm, δ 153.21 ppm, and δ 155.66 ppm did not connect to H-4 but to H-5. This indicated that the three carbon atoms were in the same ring as H-5, but in different ring with H-4. The next identification was carbon atom with δ 153.21 ppm was C-6, and carbon atom with δ 155.66 ppm was C-8b since these carbon atoms were directly bonded to oxygen atoms, which had large electronegativity, pulling the electron distribution.

A. Results of ^{1}H-NMR

The followings are 5 chromatograms as the result of ^{1}H-NMR analysis of the isolated compound obtained from the bioassay-guided fractionation isolation

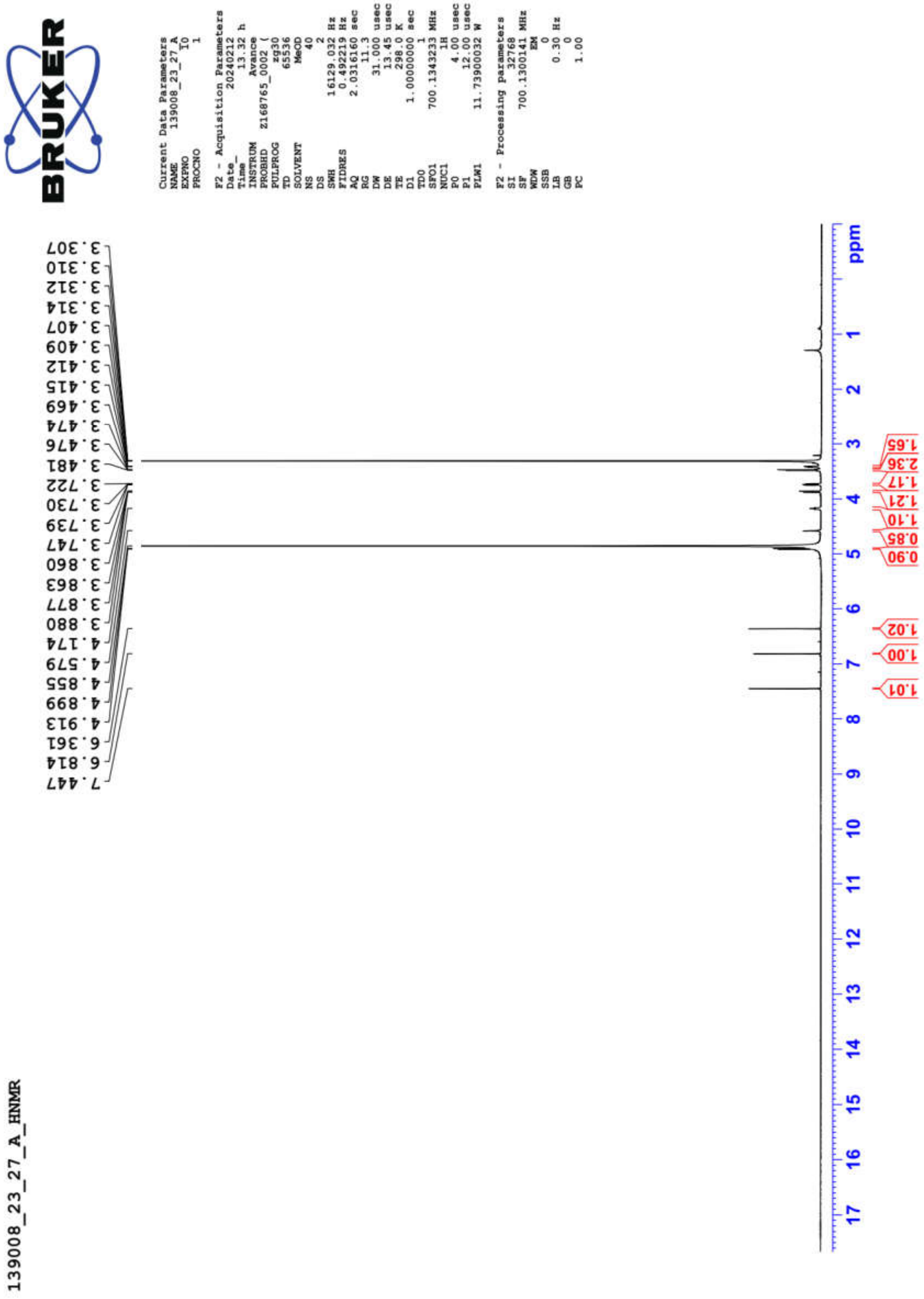

Fig A1. Chromatogram of ^{1}H-NMR of the isolated compound part 1

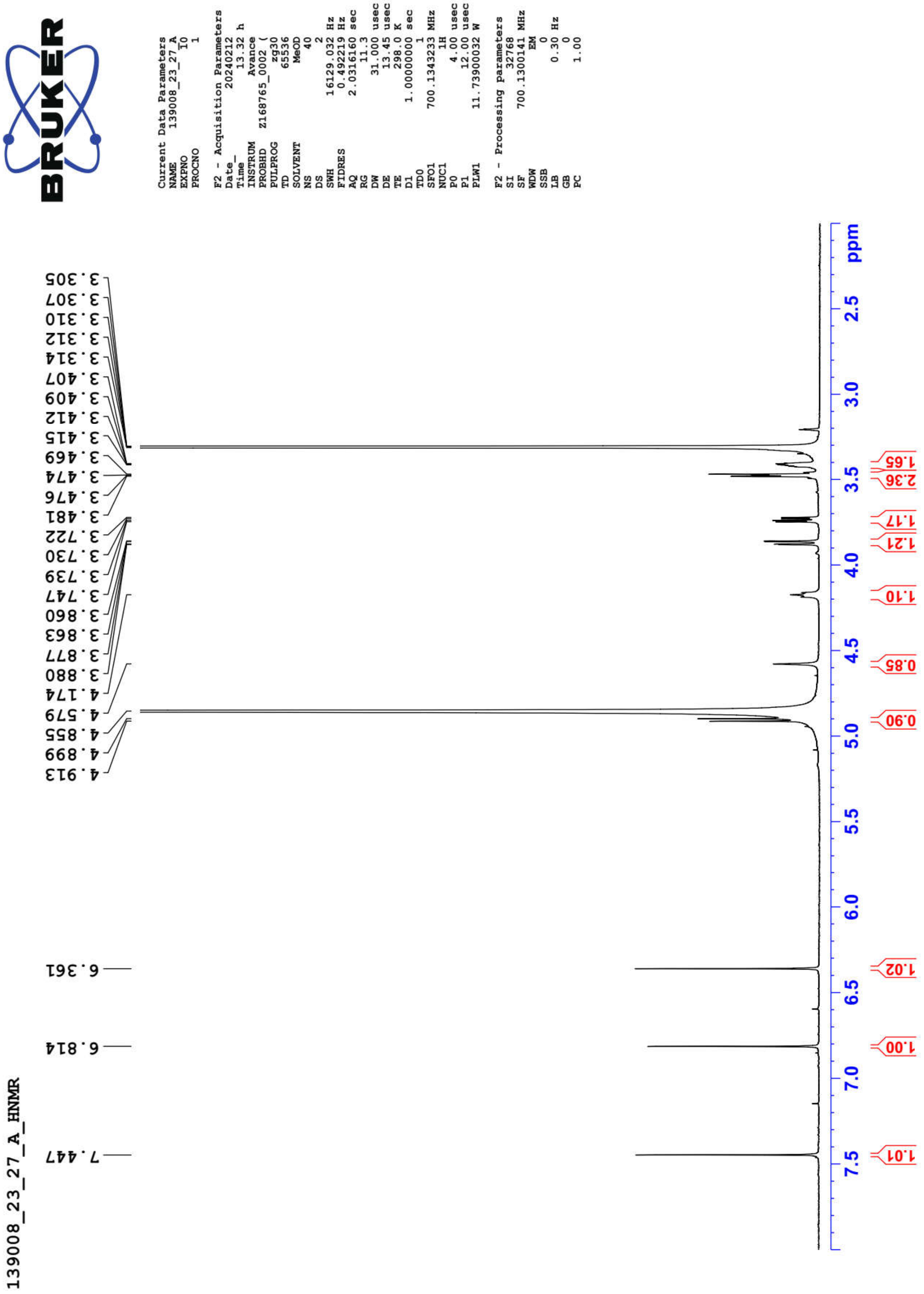

Fig A2. Chromatogram of ^{1}H-NMR of the isolated compound part 2

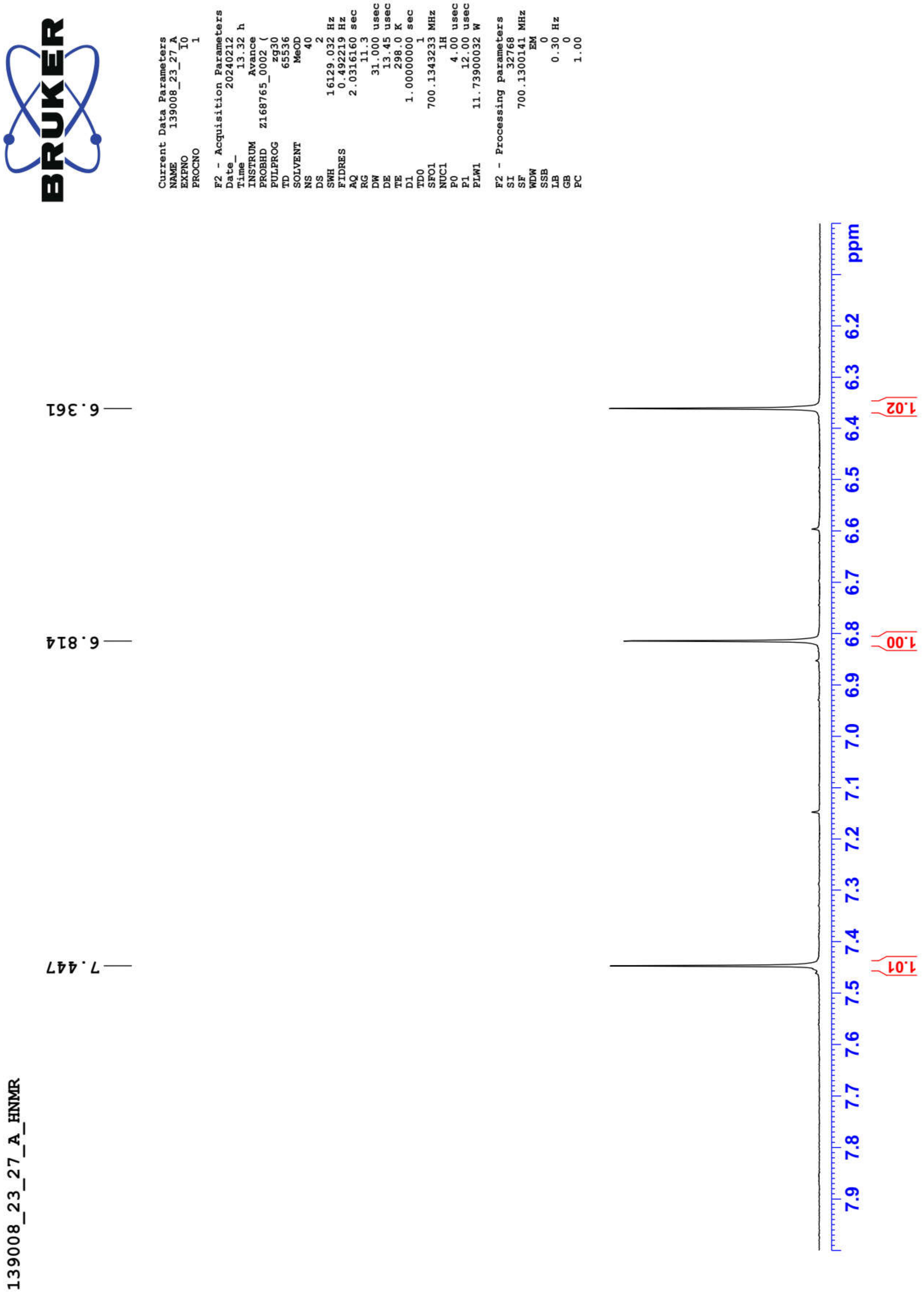

Fig A3. Chromatogram of ^{1}H-NMR of the isolated compound part 3

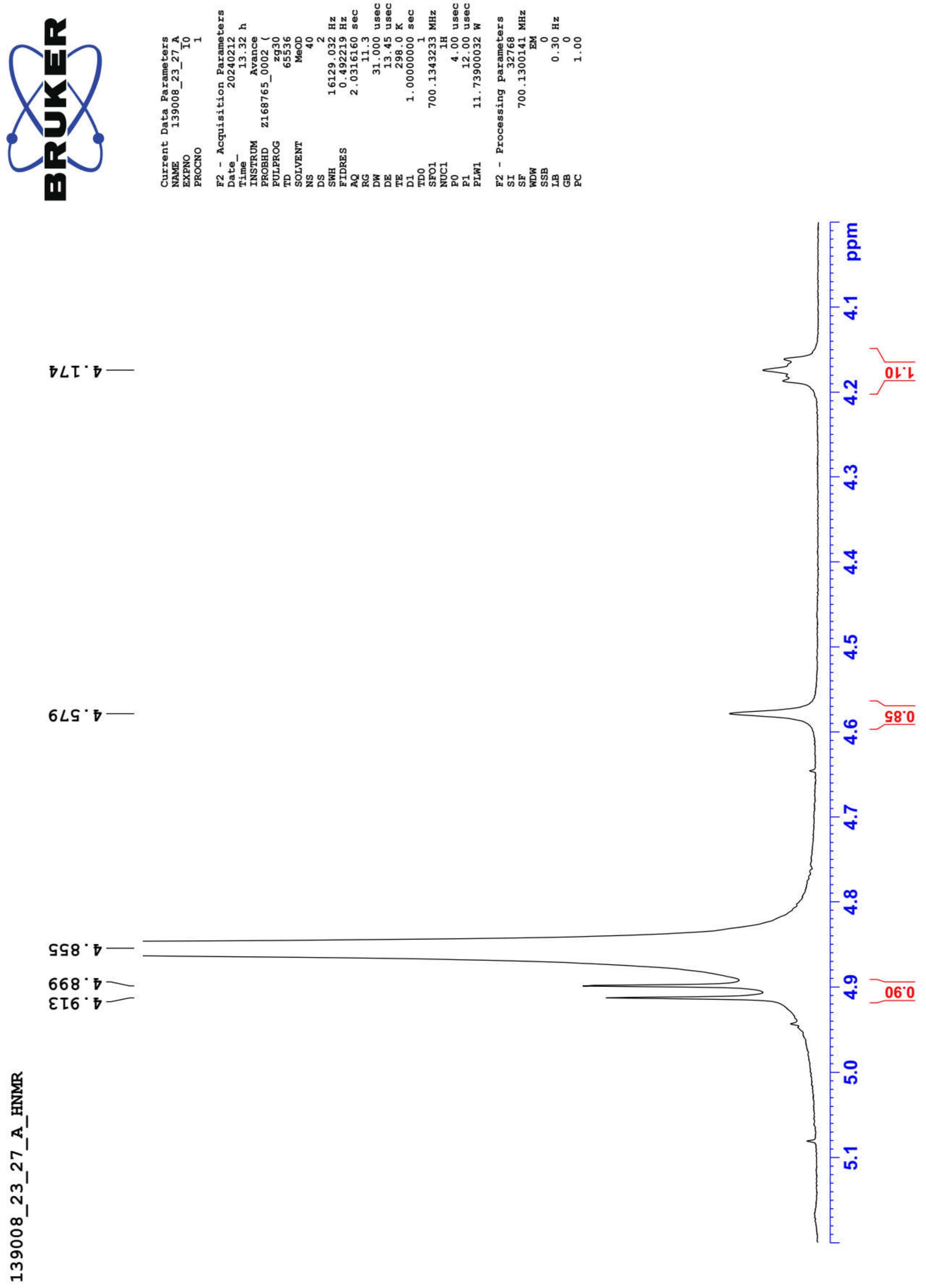

Fig A4. Chromatogram of ^{1}H-NMR of the isolated compound part 4

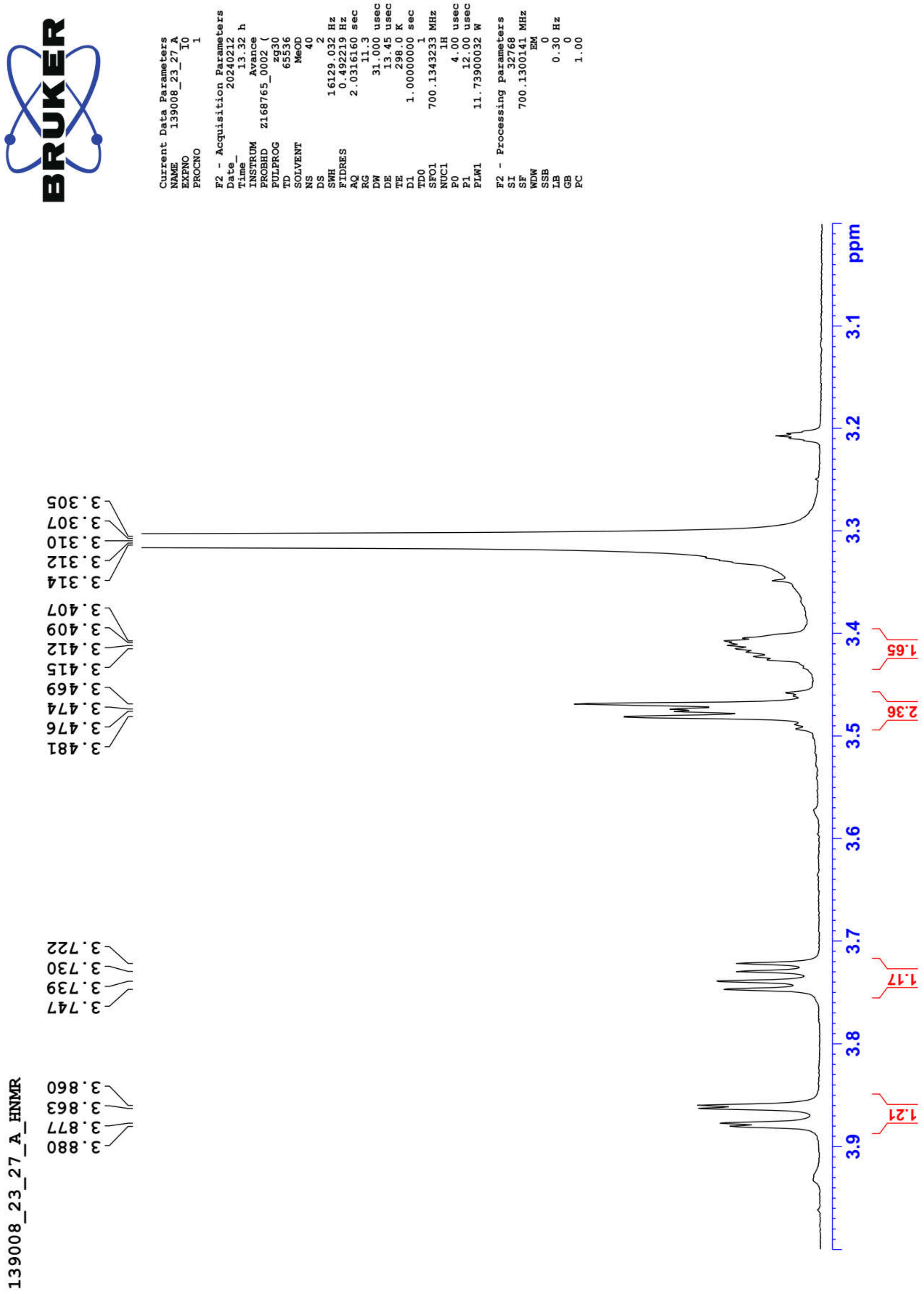

Fig A5. Chromatogram of ^{1}H-NMR of the isolated compound part 5

B. Results of ^{13}C-NMR

The followings are 7 chromatograms as the result of ^{13}C-NMR analysis of the isolated compound obtained from bioassay-guided fractionation isolation

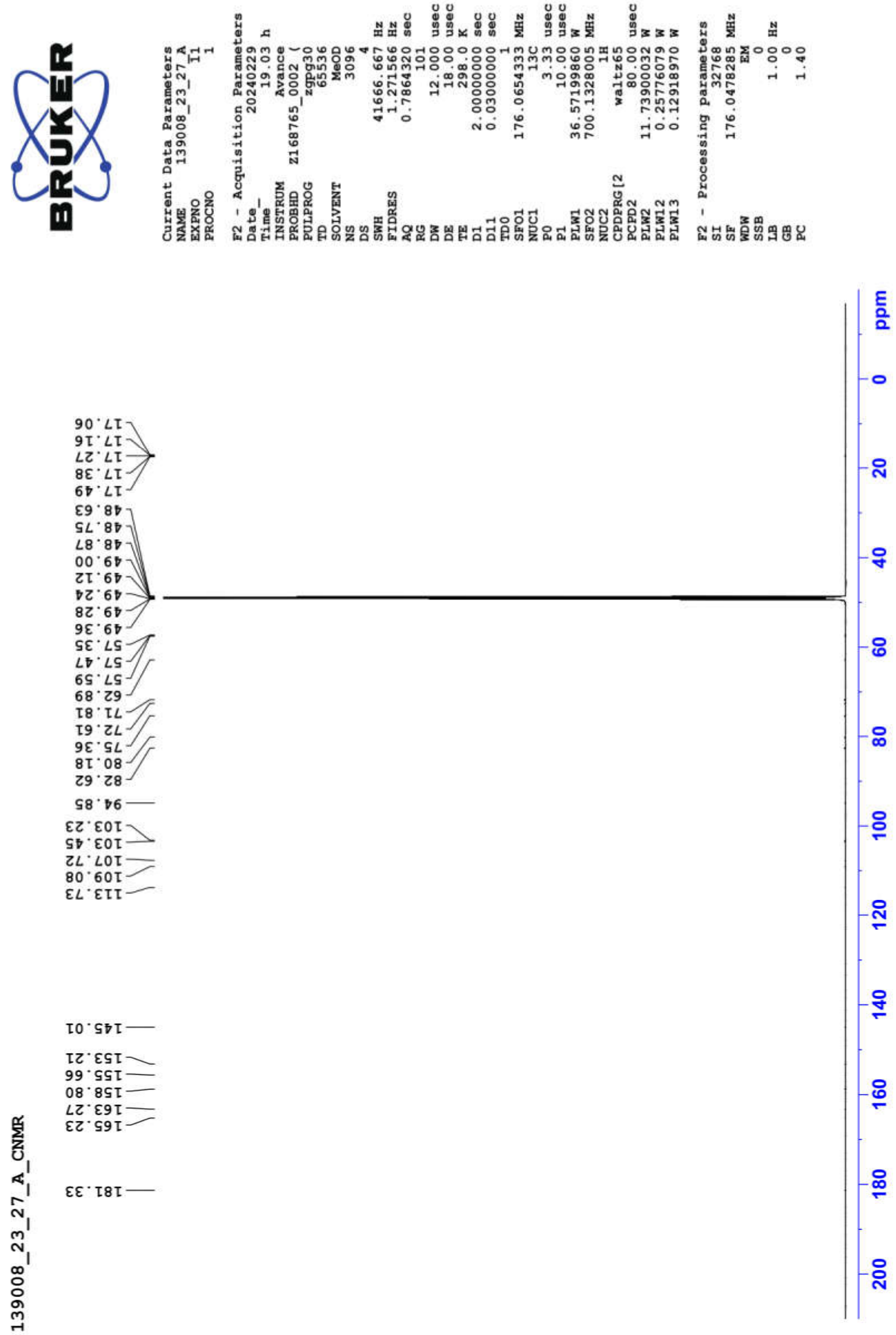

Fig B1. Chromatogram of ^{13}C-NMR of the isolated compound part 1

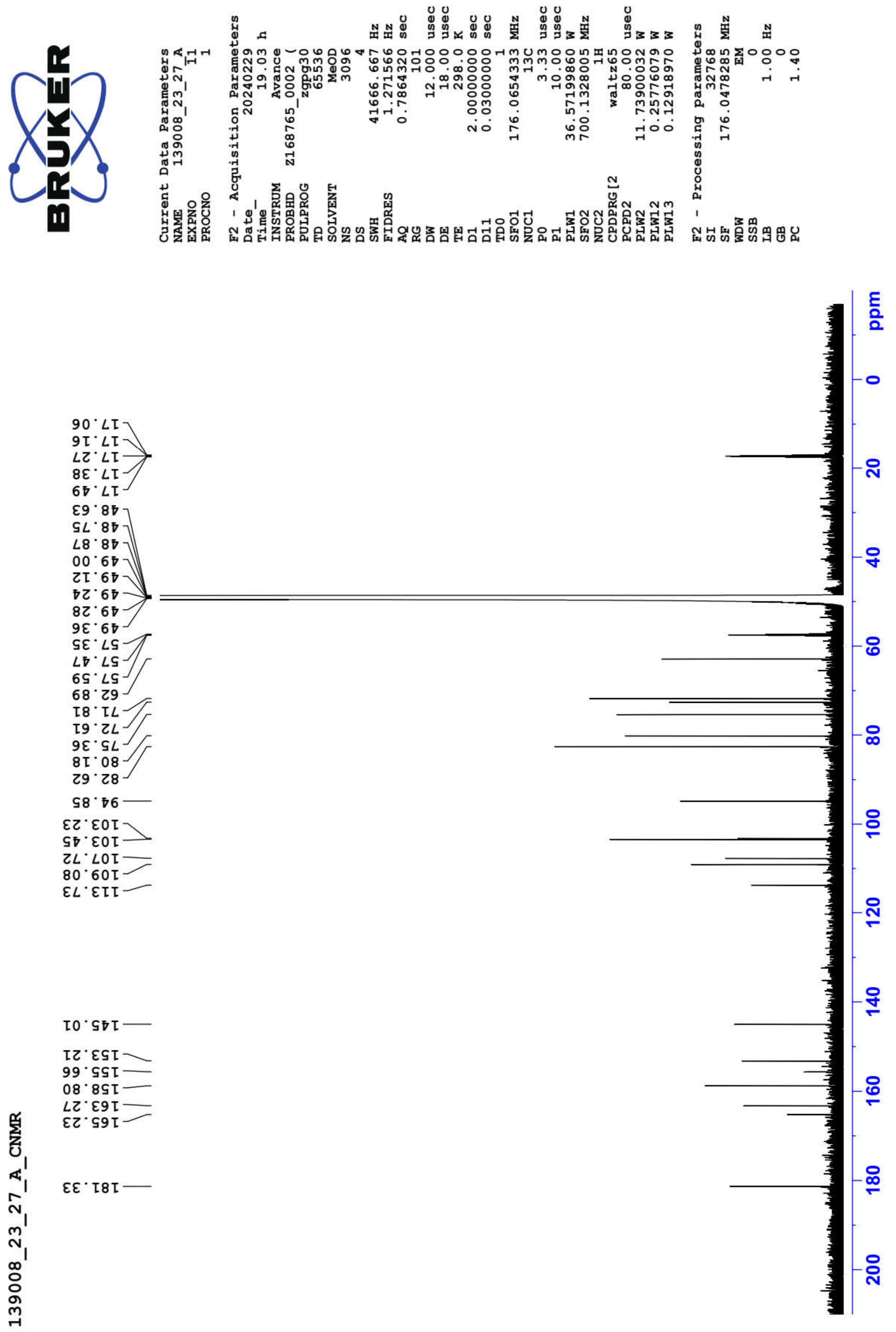

Fig B2. Chromatogram of ^{13}C-NMR of the isolated compound part 2

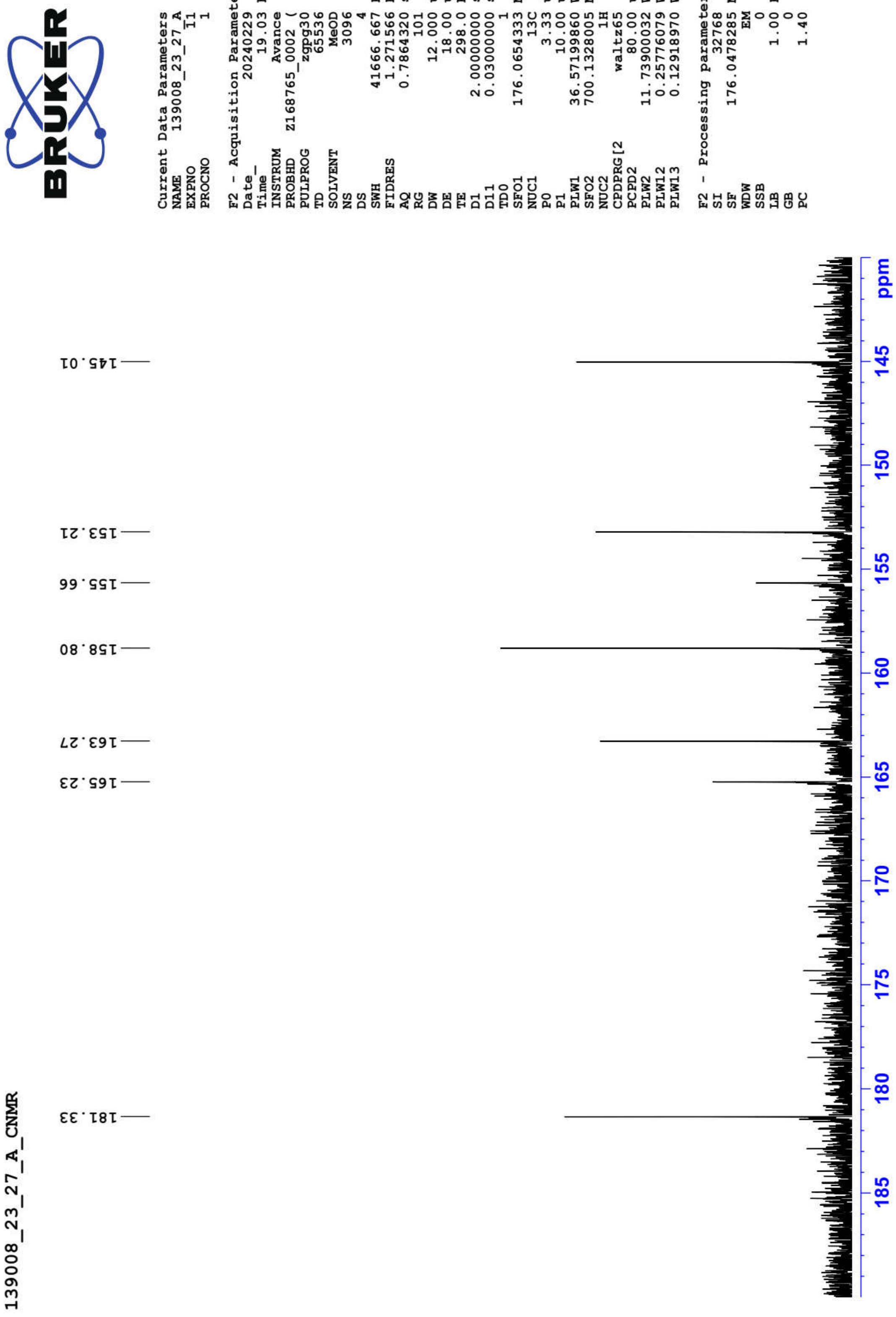

Fig B3. Chromatogram of ^{13}C-NMR of the isolated compound part 3

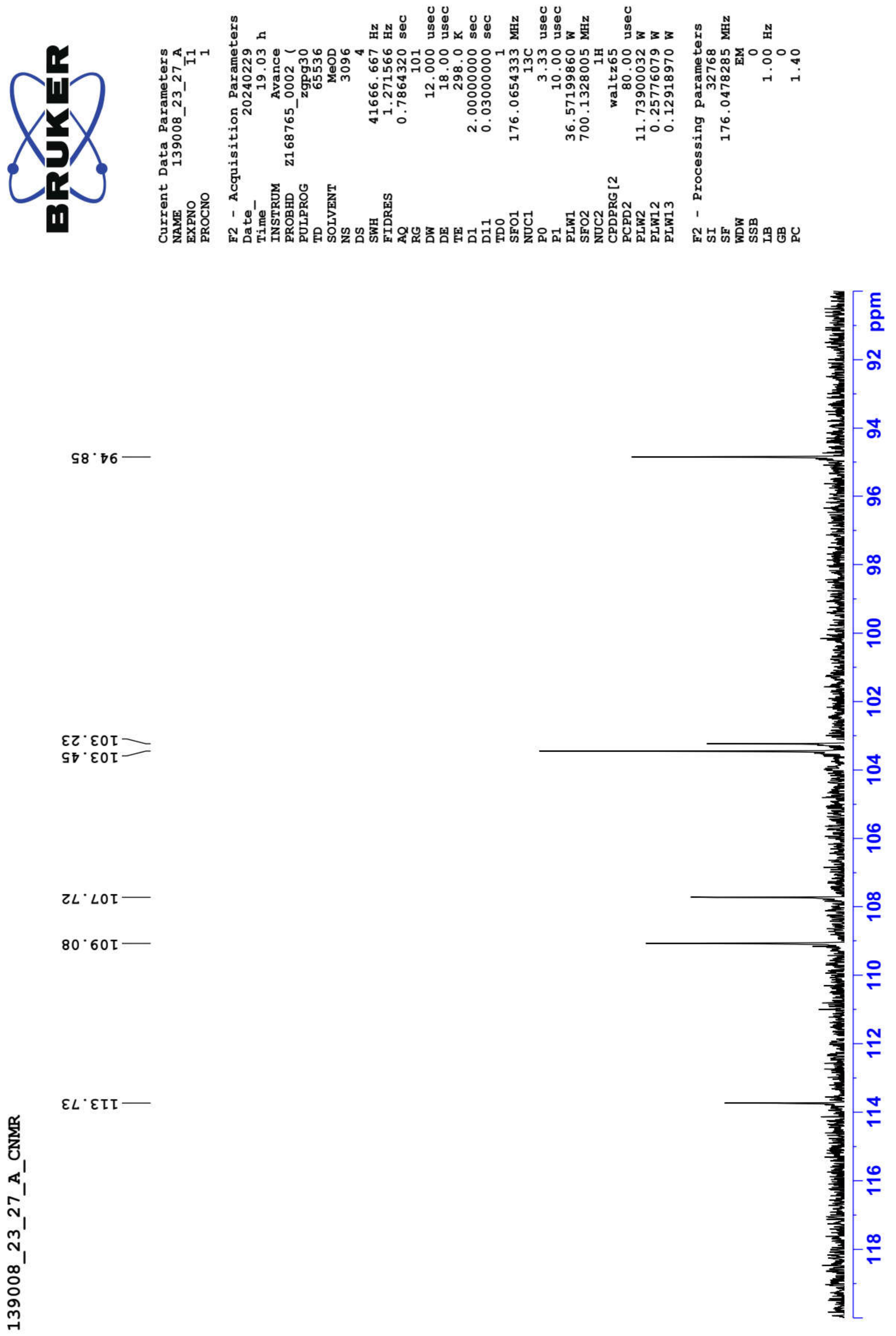

Fig B4. Chromatogram of ^{13}C-NMR of the isolated compound part 4

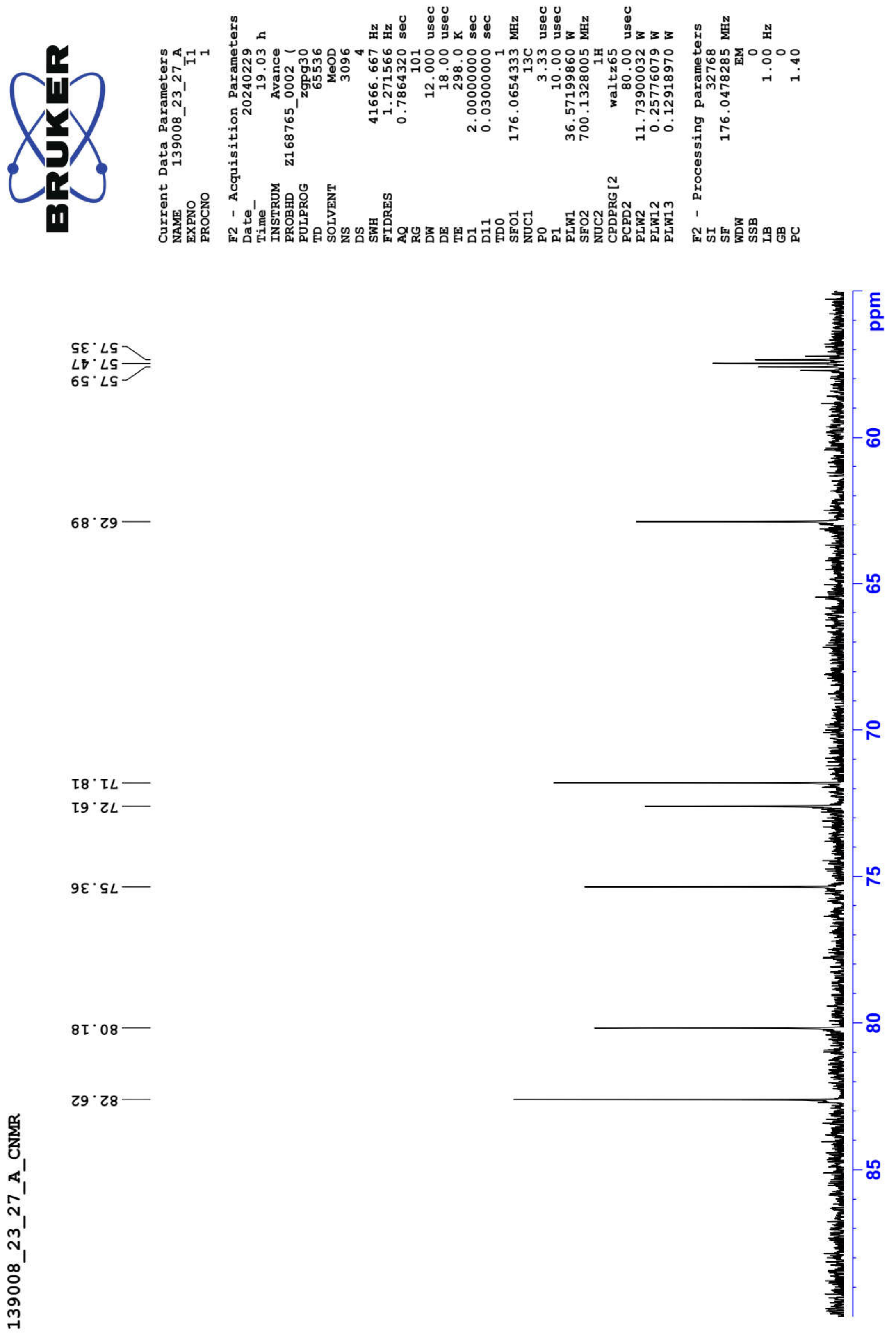

Fig B5. Chromatogram of ^{13}C-NMR of the isolated compound part 5

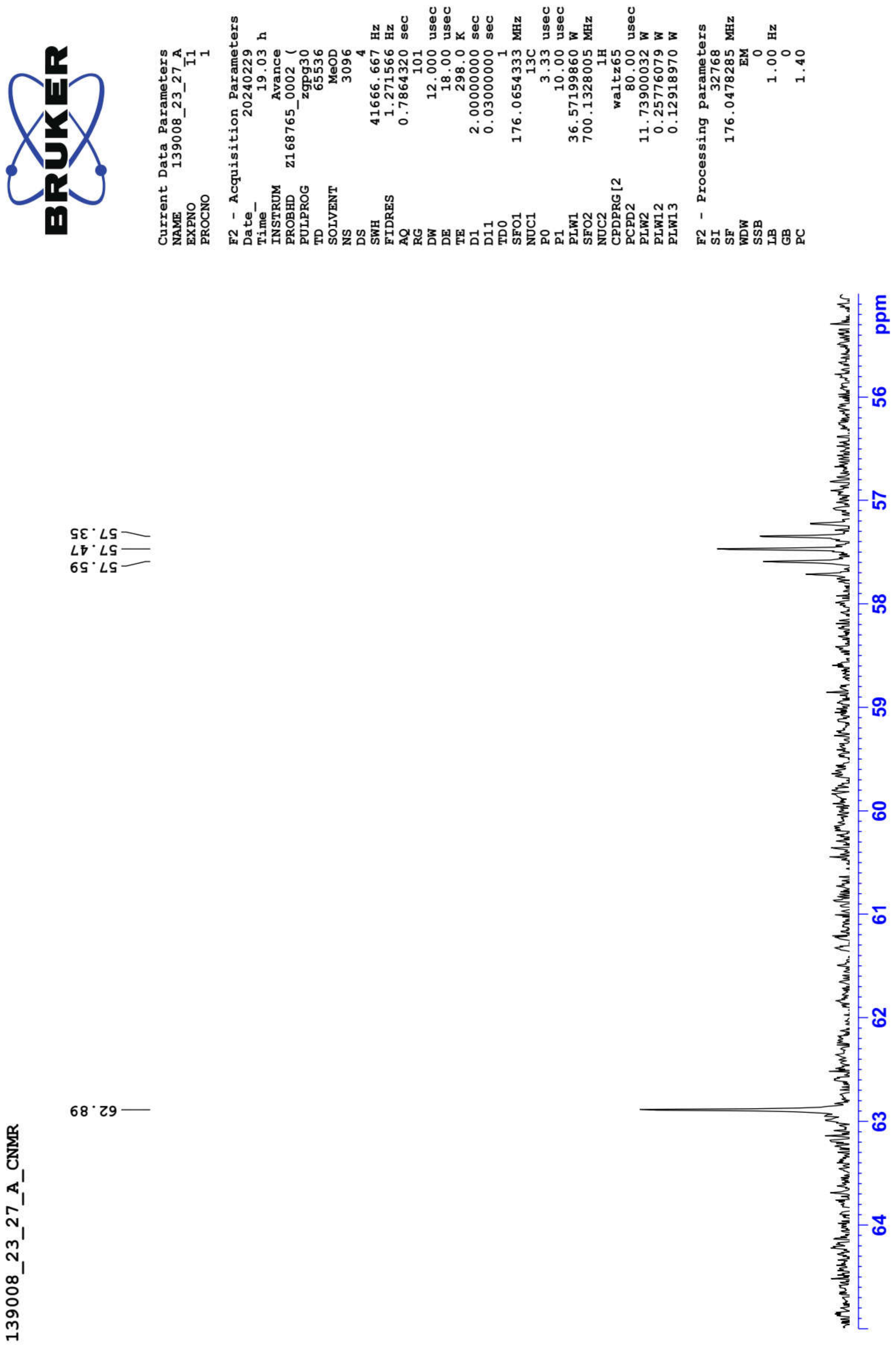

Fig B6. Chromatogram of ^{13}C-NMR of the isolated compound part 6

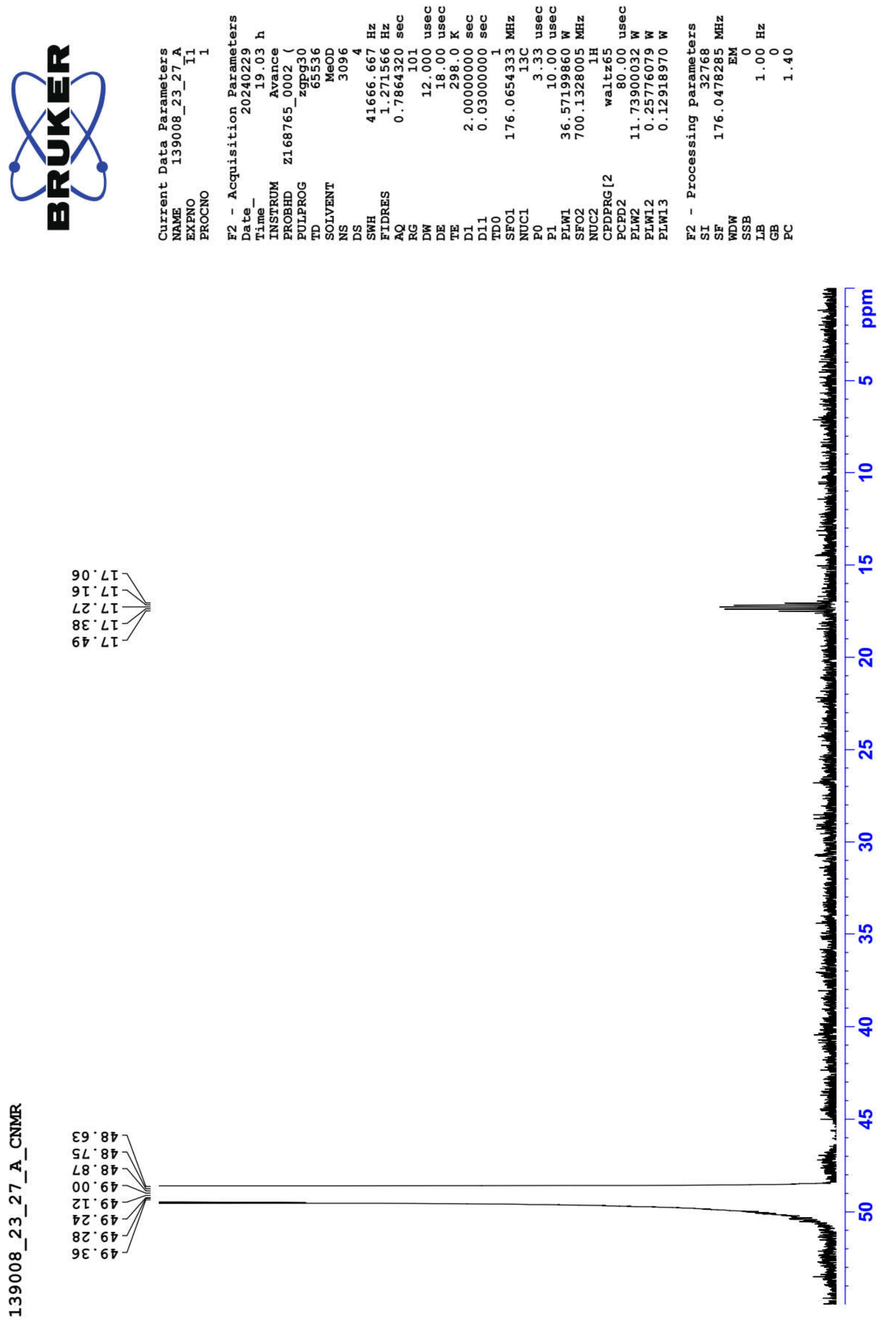

Fig B7. Chromatogram of ^{13}C-NMR of the isolated compound part 7

C. Results of 2D NMR HSQC

The followings are the 4 chromatograms as the result of 2D NMR HSQC (heteronuclear single quantum coherence) of the isolated compound obtained from bioassay-guided fractionation isolation

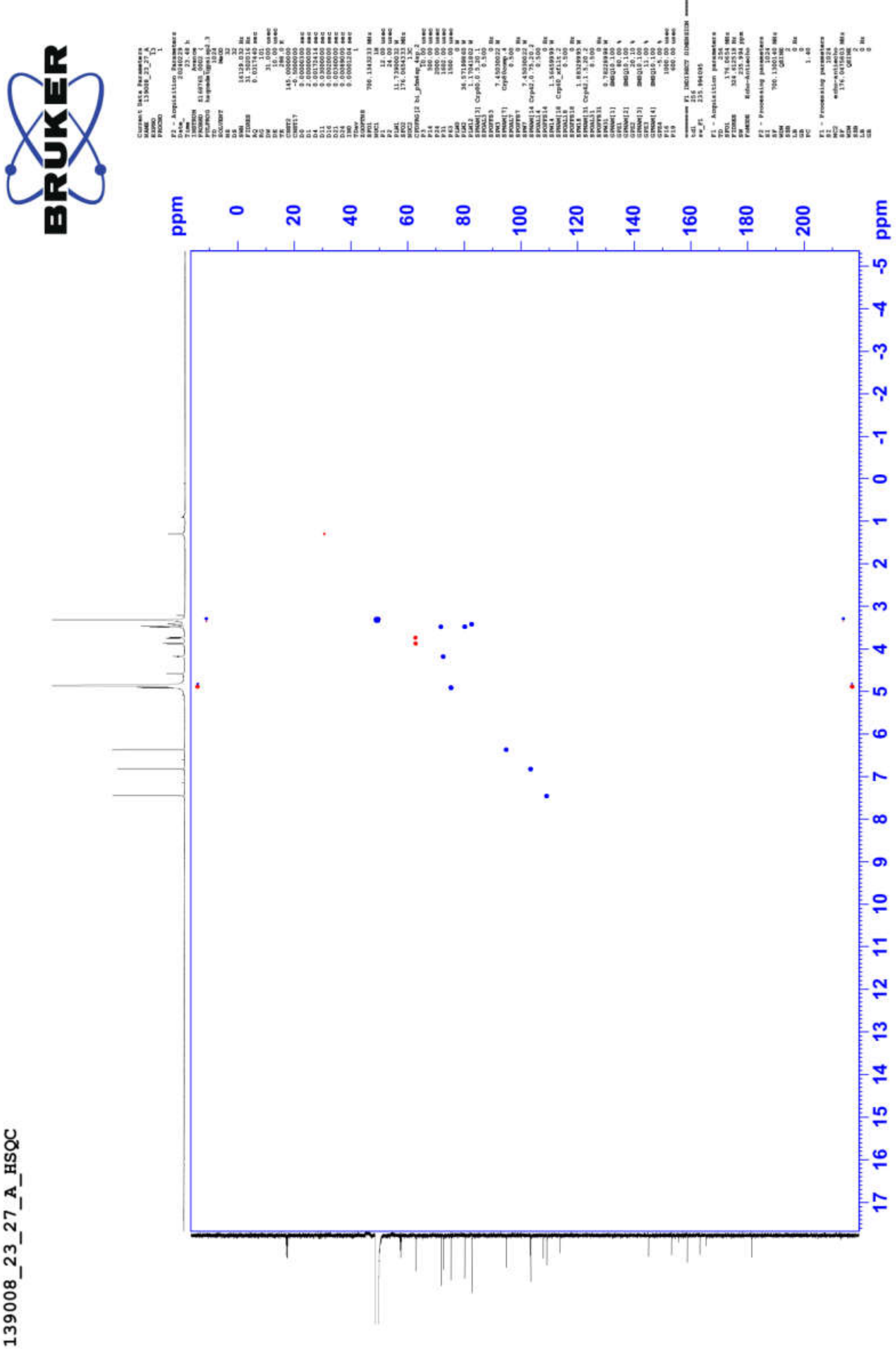

Fig C1. Chromatogram of 2D NMR HSQC of the isolated compound part 1

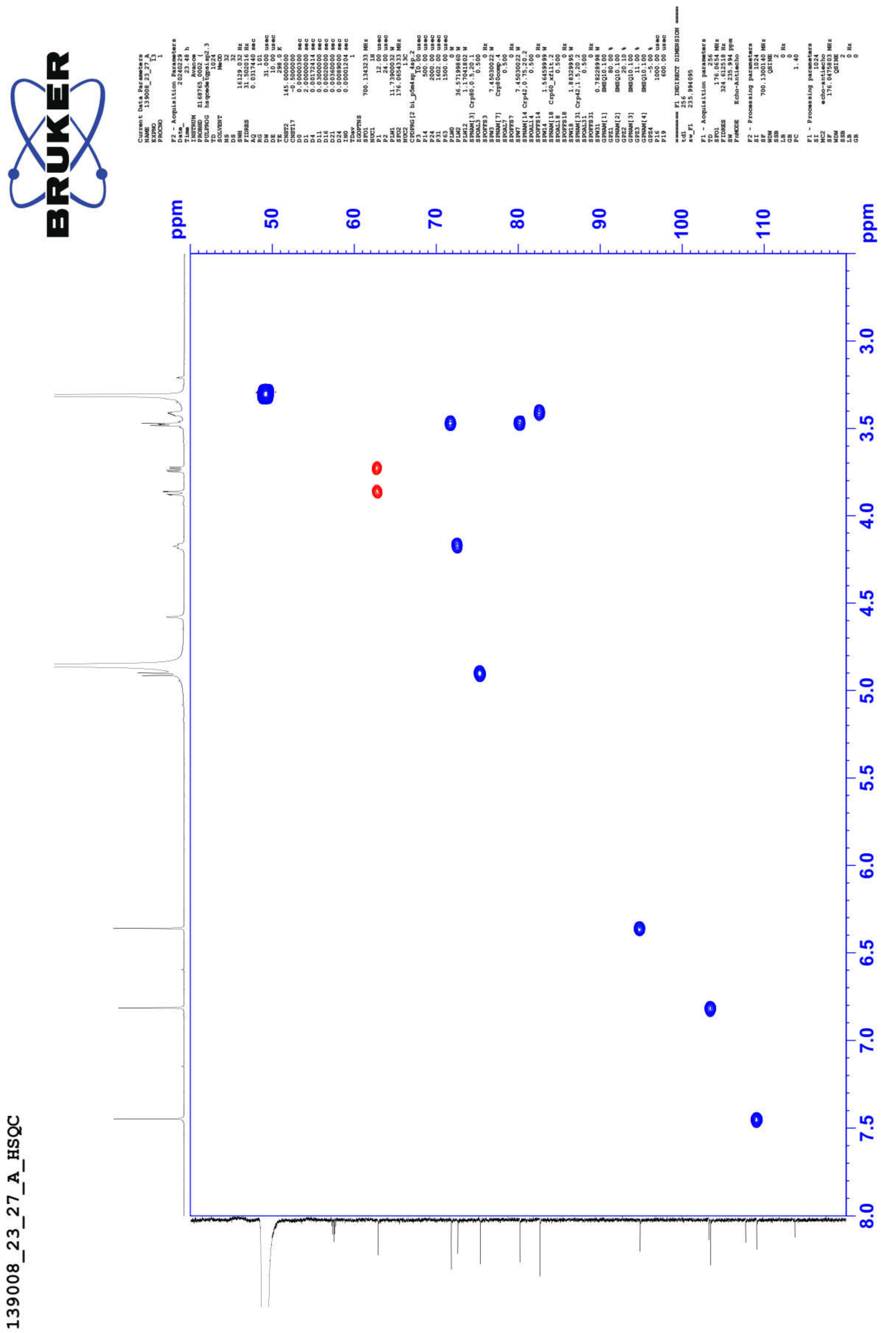

Fig C2. Chromatogram of 2D NMR HSQC of the isolated compound part 2

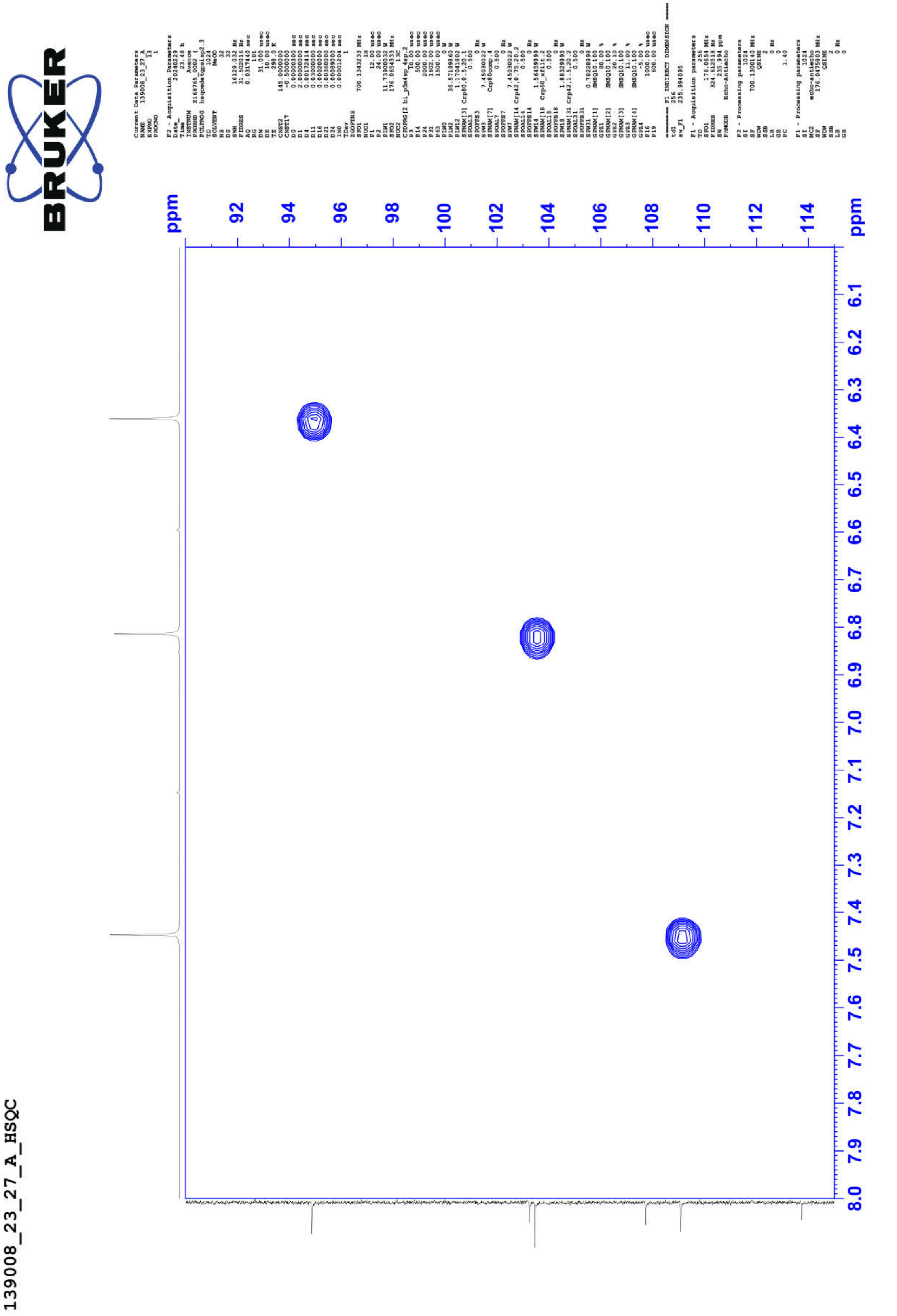

Fig C3. Chromatogram of 2D NMR HSQC of the isolated compound part 3

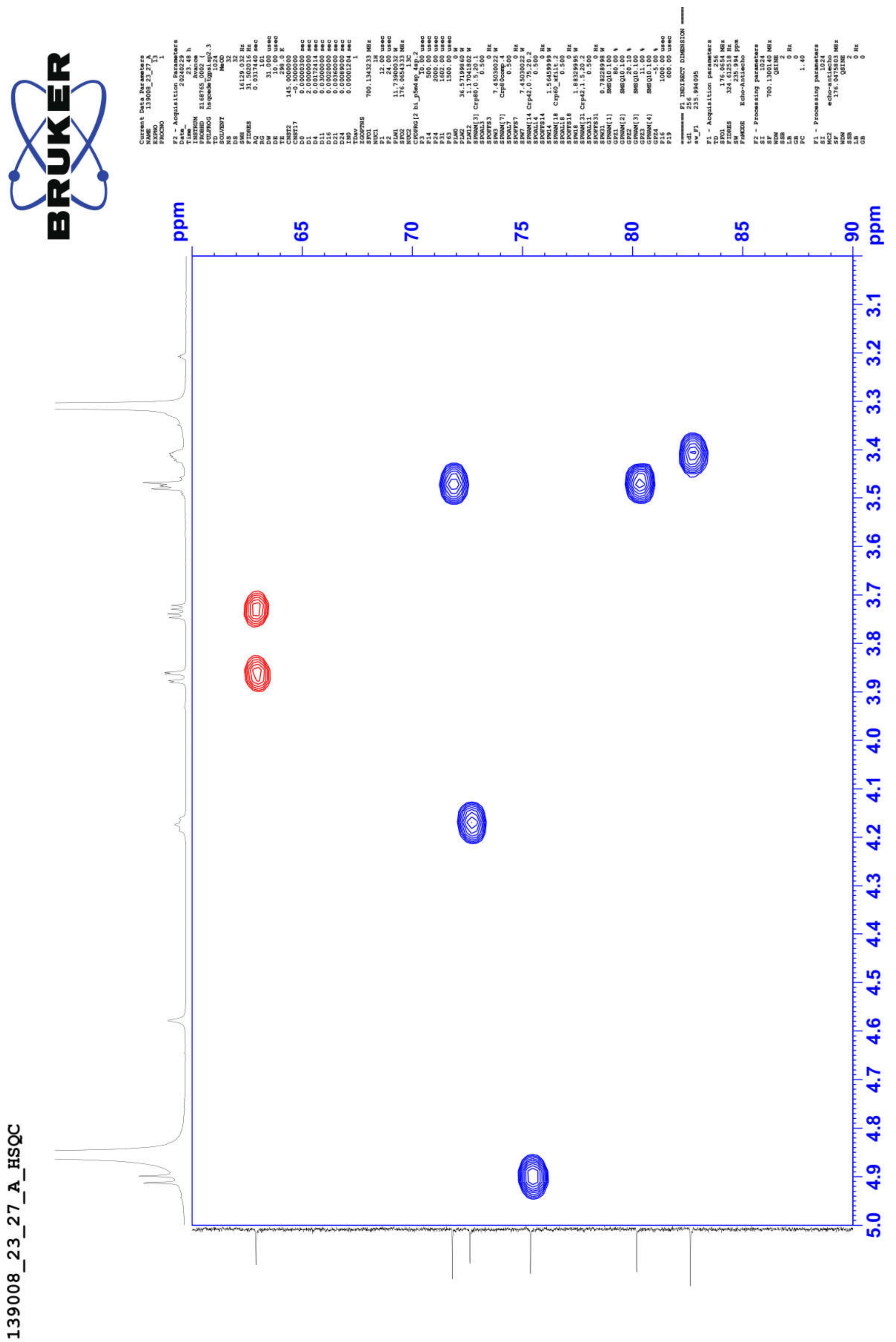

Fig C4. Chromatogram of 2D NMR HSQC of the isolated compound part 4

D. Results of 2D NMR HMBC

The followings are 7 chromatograms as the results of 2D NMR HMBC (heteronuclear multiple bond correlation) of the isolated compound from the bioassay guided fractionation isolation

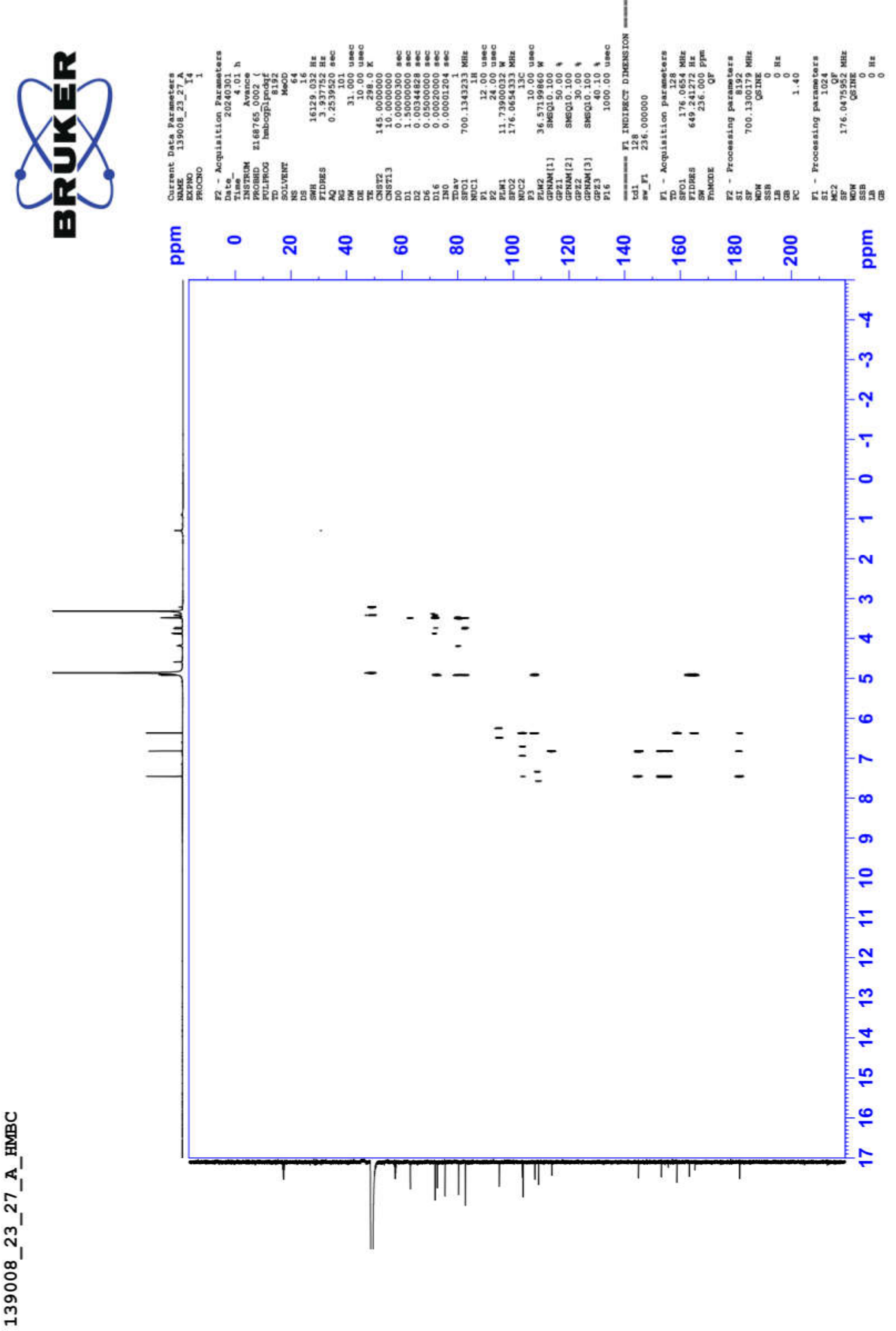

Fig D1. Chromatogram of 2D NMR HMBC of the isolated compound part 1

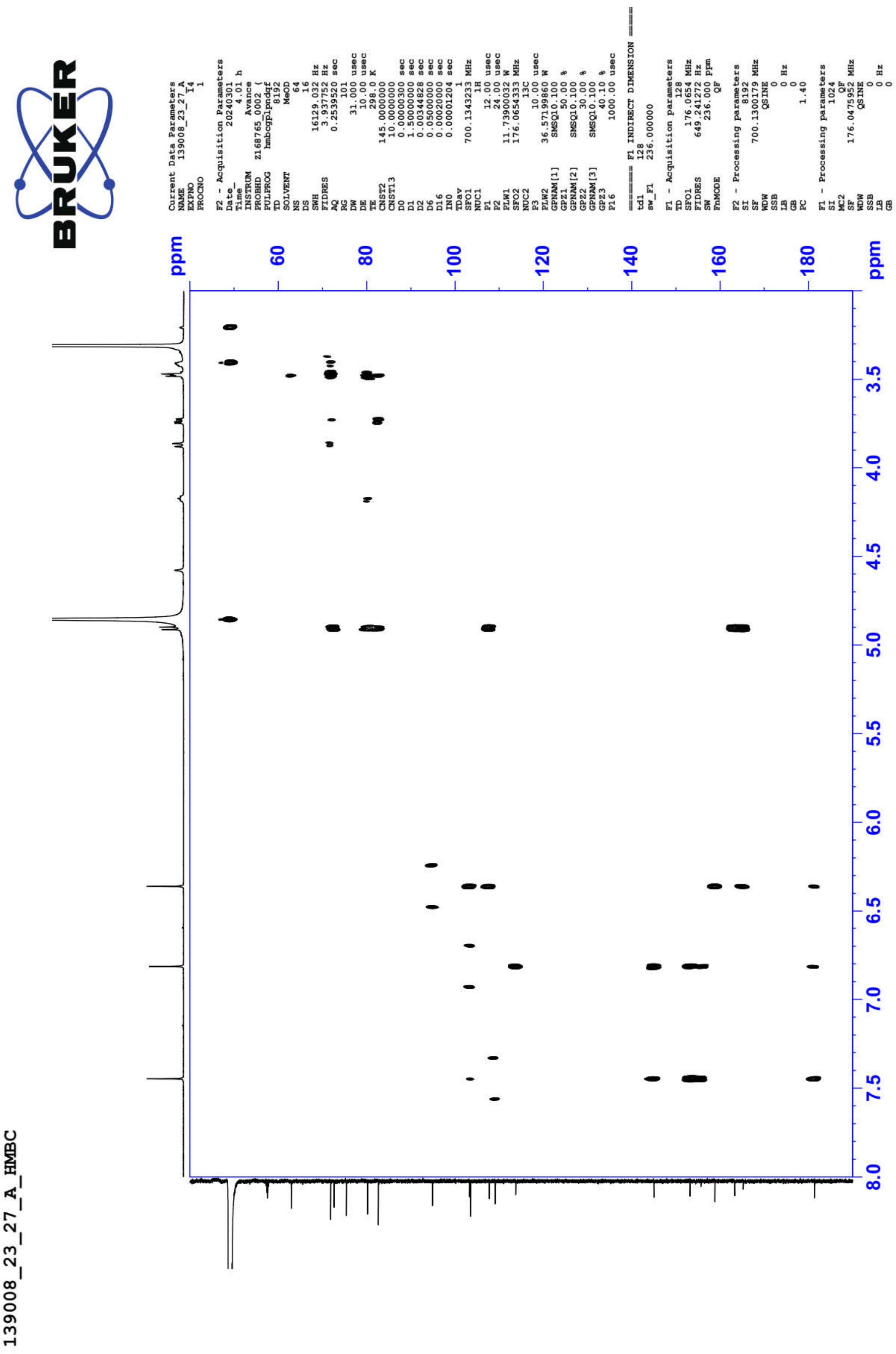

Fig D2. Chromatogram of 2D NMR HMBC of the isolated compound part 2

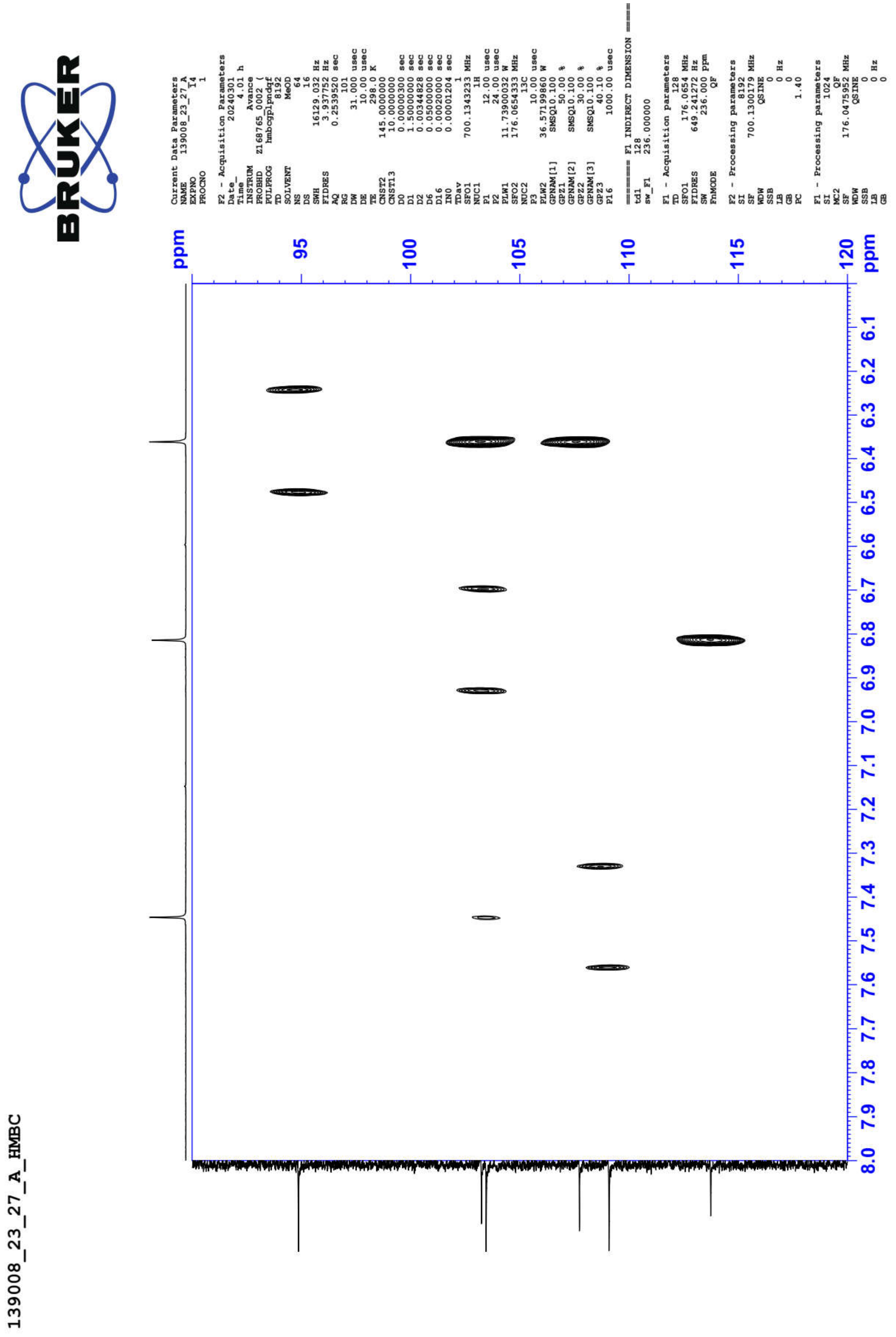

Fig D3. Chromatogram of 2D NMR HMBC of the isolated compound part 3

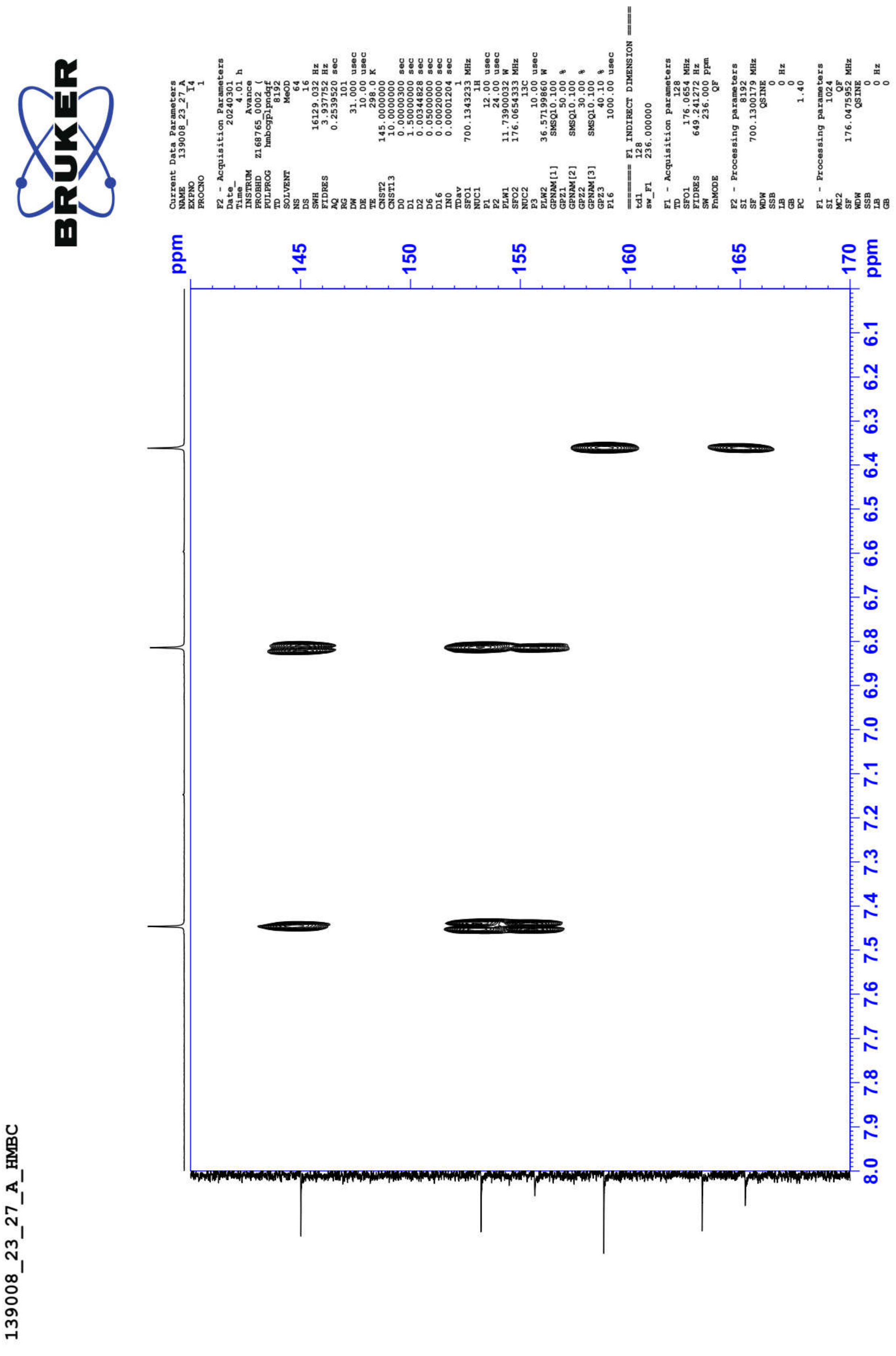

Fig D4. Chromatogram of 2D NMR HMBC of the isolated compound part 4

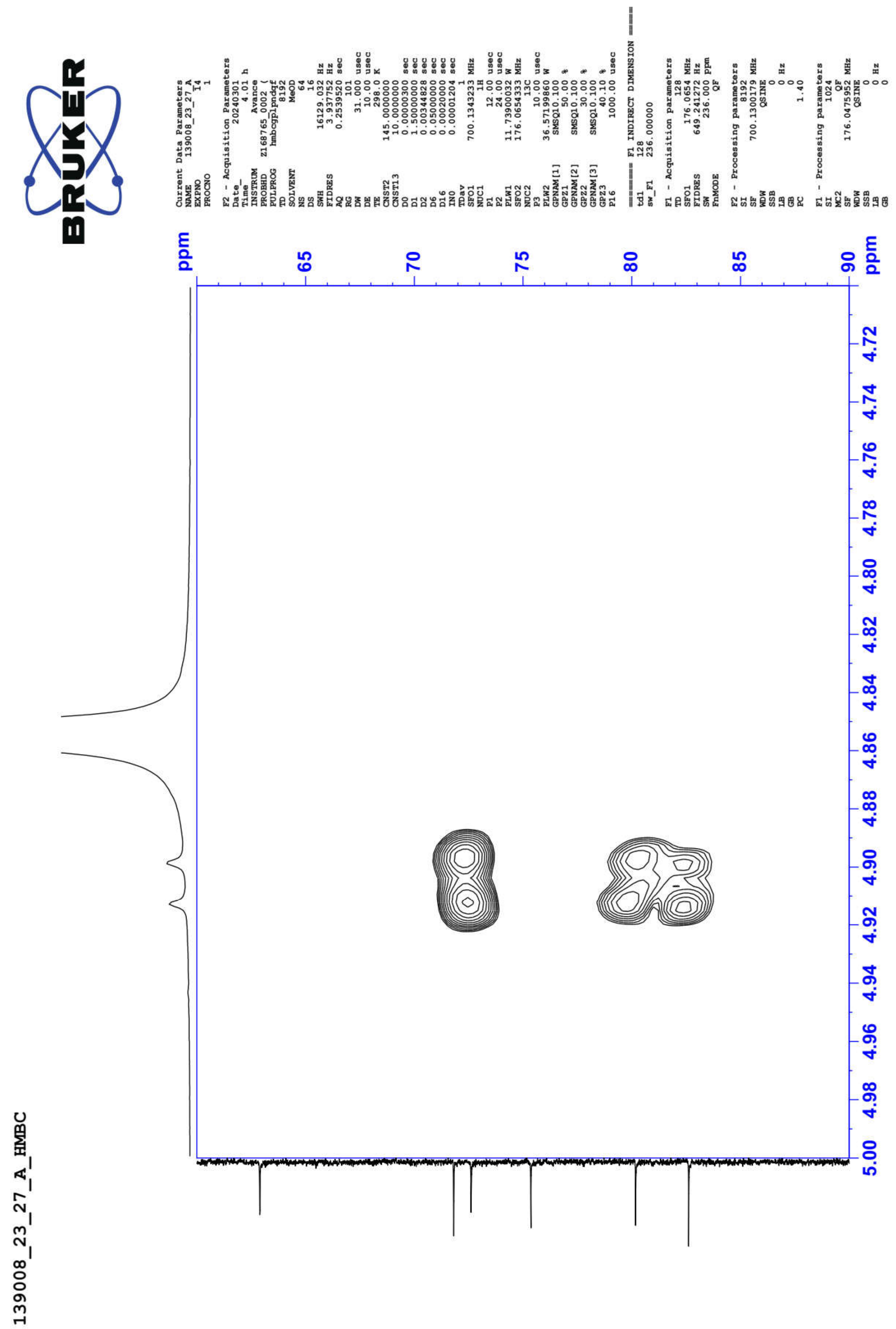

Fig D5. Chromatogram of 2D NMR HMBC of the isolated compound part 5

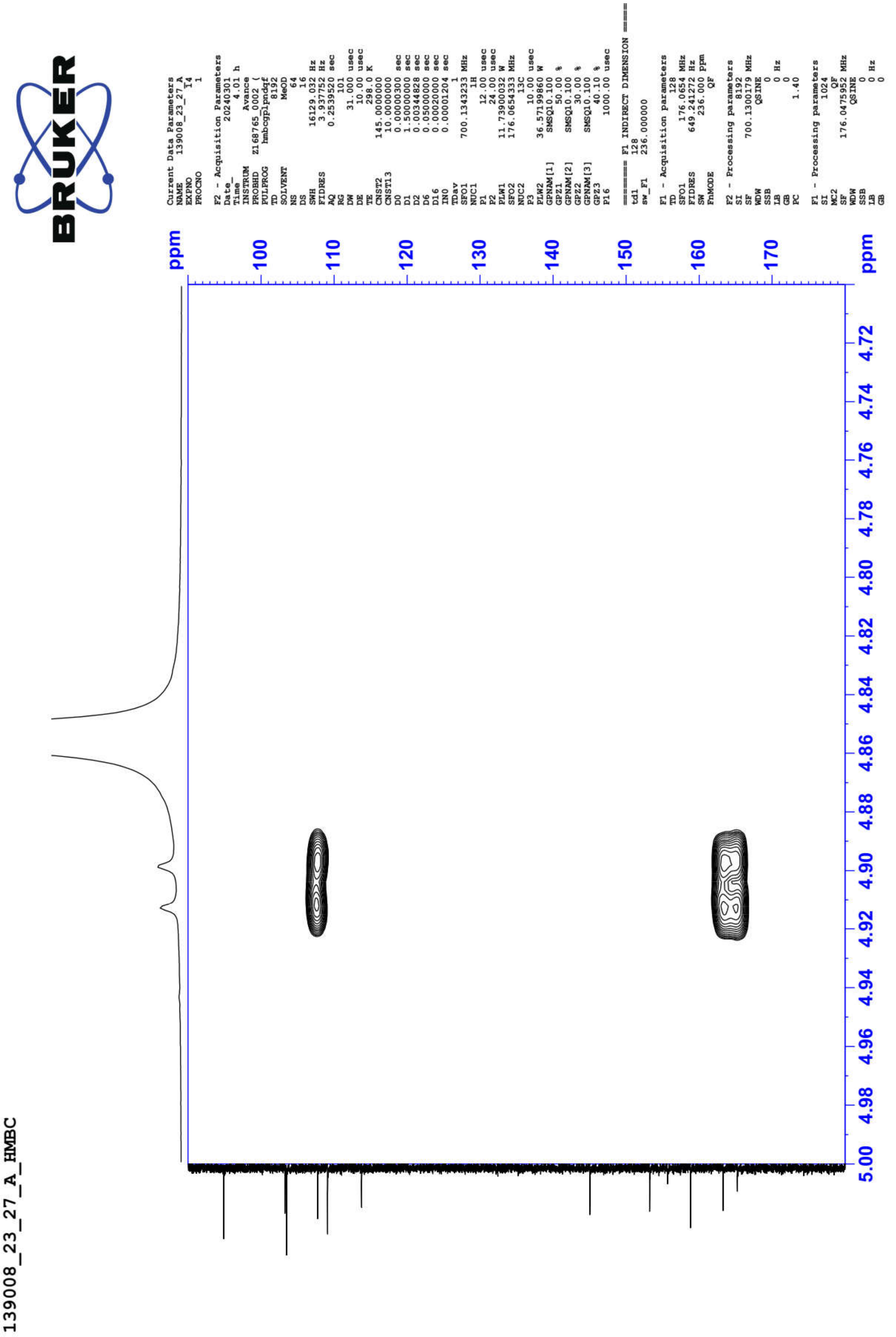

Fig D6. Chromatogram of 2D NMR HMBC of the isolated compound part 6

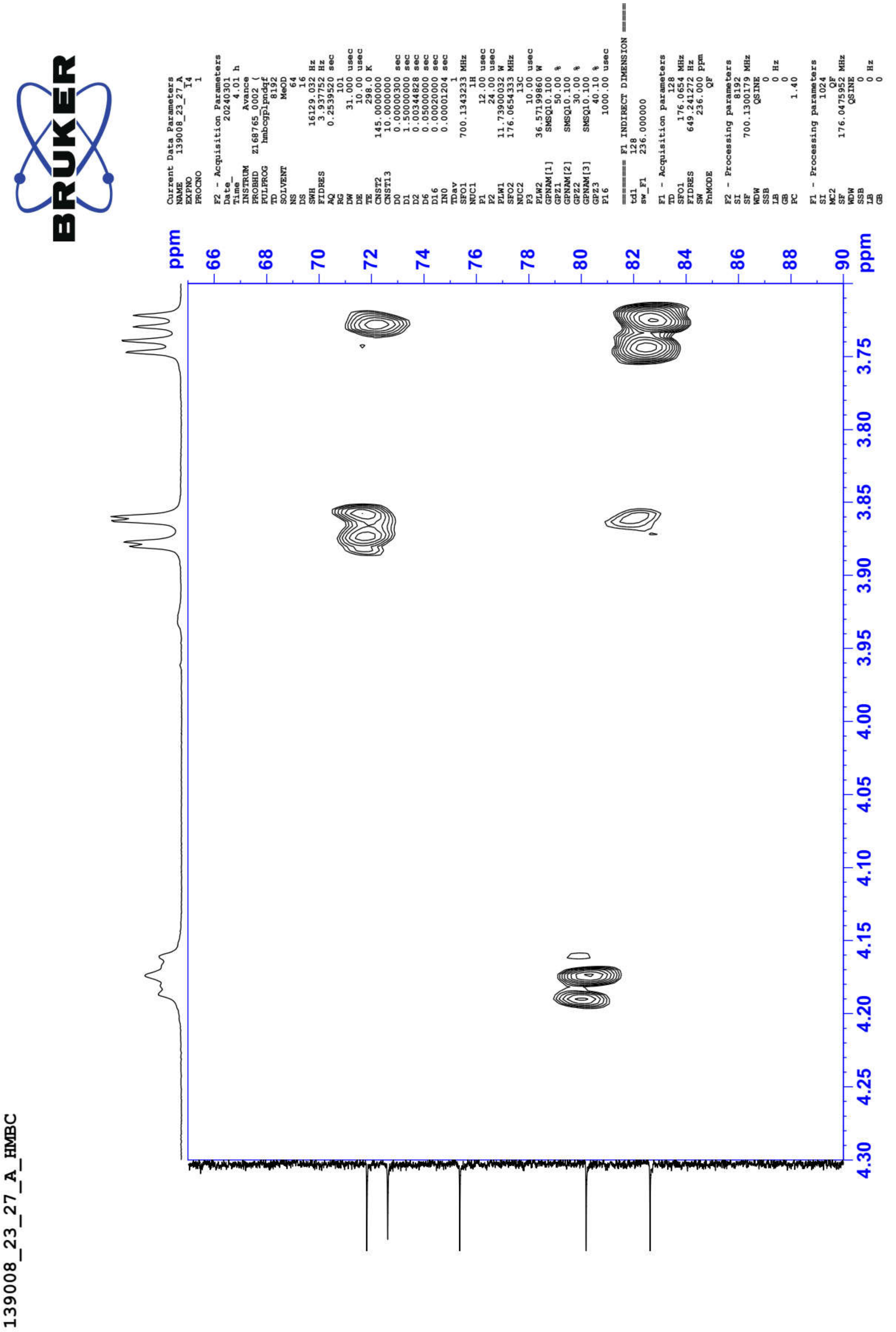

Fig D7. Chromatogram of 2D NMR HMBC of the isolated compound part 7
