# Supplementary material for: Investigation of alpha-glucosidase inhibition activity of Artabotrys sumatranus leaf extract using metabolomics, machine learning and molecular docking analysis
Source: PLoS One. 2025 Jan 3;20(1):e0313592. doi: 10.1371/journal.pone.0313592 (PMC11698457; doi:10.1371/journal.pone.0313592)
Supplement: S3 File — In this file, the binding sites of the identified predicted active compounds to 3A4A receptors and 3TOP receptors are shown. As comparison, the position and orientation of acarbose as a result of molecular docking are shown. Acarbose occupied the same binding site as the native ligands of the 3A4A and 3TOP receptors. (PDF) [file pone.0313592.s010.pdf]

S3 File. Binding sites of 15,16-dihydrotanshinone I, neomangiferin, apigenin-7-O-galactopyranoside, lirioferin, and norisocorydine to 3A4A and 3TOP receptors.

In this file, the binding sites of the identified predicted active compounds to 3A4A receptors and 3TOP receptors are shown. As comparison, the position and orientation of acarbose as a result of docking are shown. Acarbose occupied the same binding site as the native ligands of the 3A4A and 3TOP receptors.

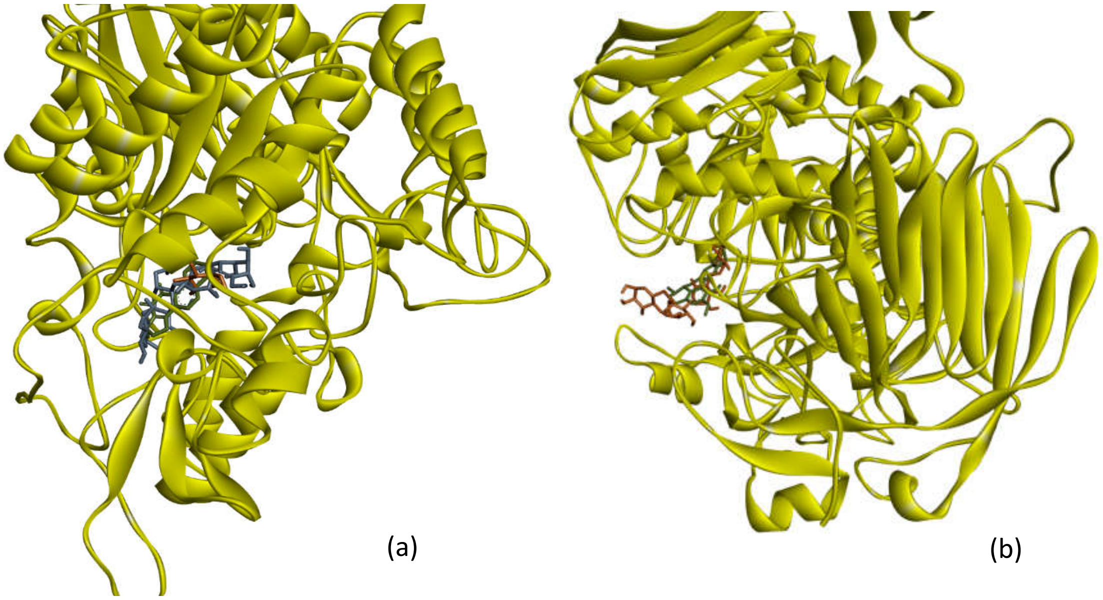

Fig S3.1. Position and 3D orientation of molecular docking results of 15,16 dihydrotanshinone I to (a) 3A4A receptor and (b) 3TOP receptor. In the plot the positions and orientations of acarbose which served as a comparison and native ligand of 3TOP receptor, as well as those of glucose, the native ligand of 3A4A receptor, are also shown. It can be seen that 15,16 dihydrotanshinone I and acarbose occupied the same binding sites, both for 3A4A and 3TOP receptors. The predicted active compound also occupied the same binding sites as the native ligands for both 3A4A and 3TOP receptors.

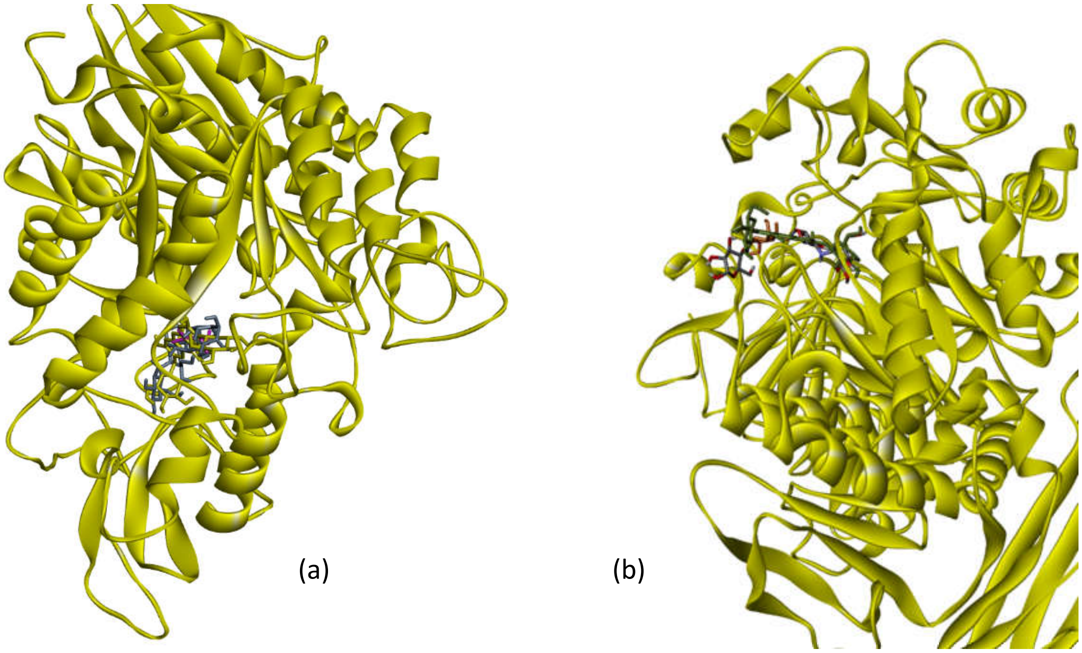

Fig S3.2. Position and 3D orientation of molecular docking results of neomangiferin to (a) 3A4A receptor and (b) 3TOP receptor. In the plot the positions and orientations of acarbose which served as a comparison and native ligand of 3TOP receptor, as well as those of glucose, the native ligand of 3A4A receptor, are also shown. It can be seen that neomangiferin and acarbose occupied the same binding sites, both for 3A4A and 3TOP receptors. The predicted active compound also occupied the same binding sites as the native ligands for both 3A4A and 3TOP receptors.

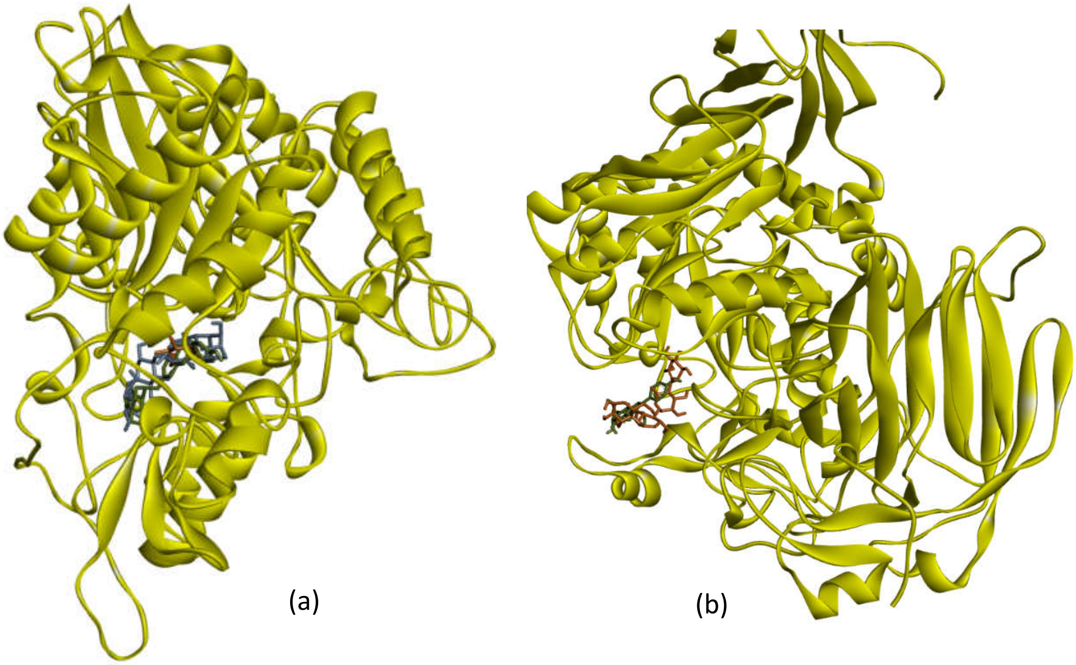

Fig S3.3. Position and 3D orientation of molecular docking results of apigenin-7-O-galactopyranoside to (a) 3A4A receptor and (b) 3TOP receptor. In the plot the positions and orientations of acarbose which served as a comparison and native ligand of 3TOP receptor, as well as those of glucose, the native ligand of 3A4A receptor, are also shown. It can be seen that apigenin-7-O-galactopyranoside and acarbose occupied the same binding sites, both for 3A4A and 3TOP receptors. The predicted active compound also occupied the same binding sites as the native ligands for both 3A4A and 3TOP receptors.

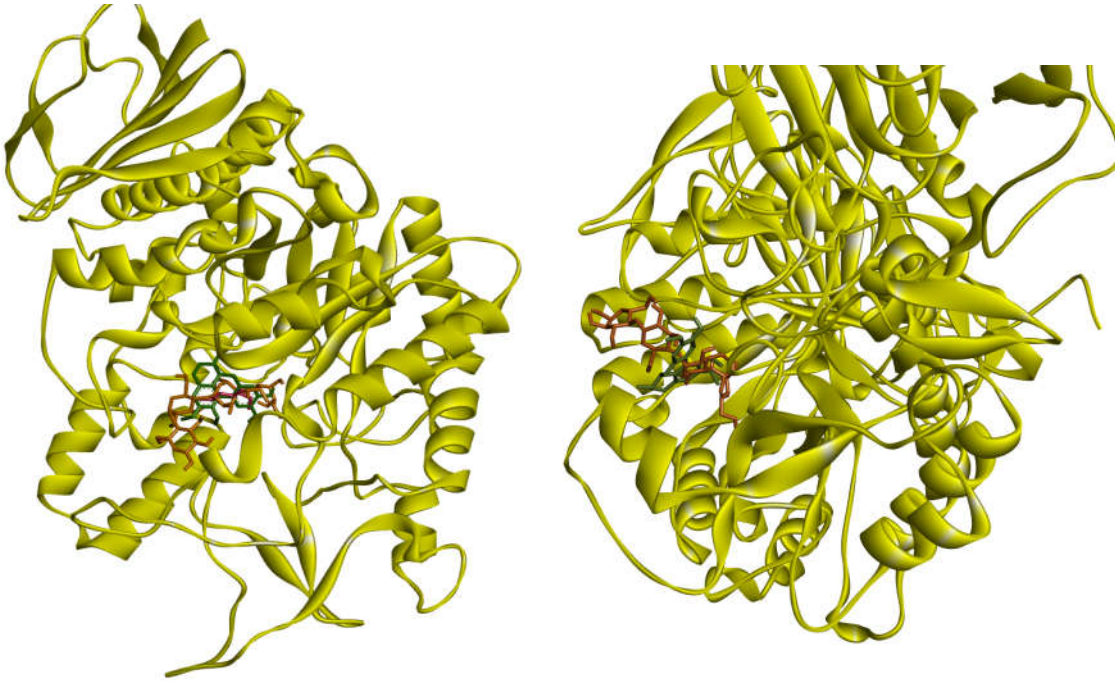

(a) (b)

Fig S3.4. Position and 3D orientation of molecular docking results of lirioferin to (a) 3A4A receptor and (b) 3TOP receptor. In the plot the positions and orientations of acarbose which served as a comparison and native ligand of 3TOP receptor, as well as those of glucose, the native ligand of 3A4A receptor, are also shown. It can be seen that lirioferin and acarbose occupied the same binding sites, both for 3A4A and 3TOP receptors. The predicted active compound also occupied the same binding sites as the native ligands for both 3A4A and 3TOP receptors.

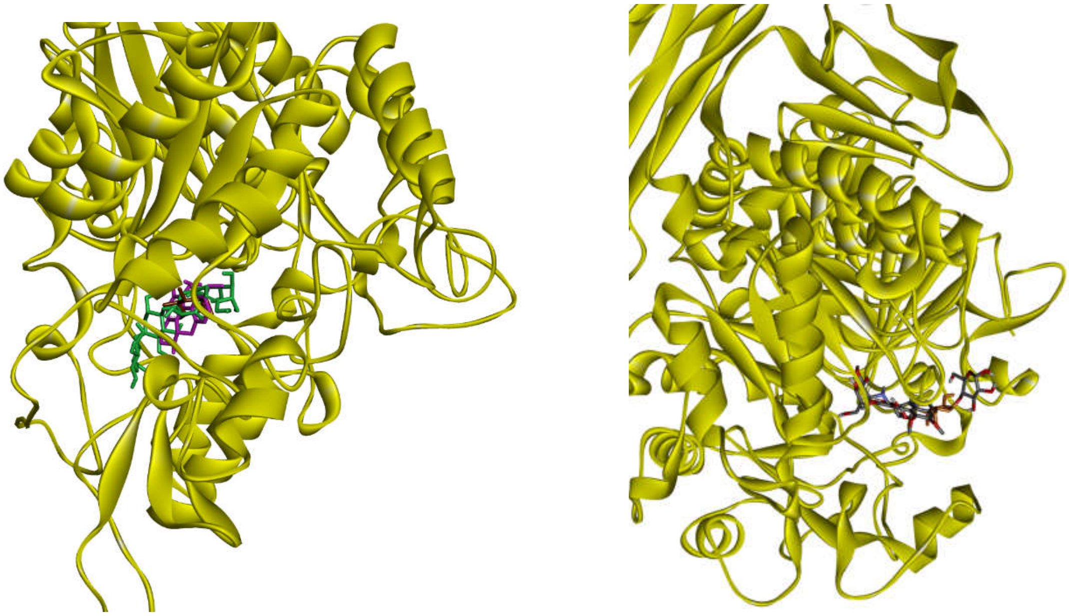

(a) (b)

Fig S3.5. Position and 3D orientation of molecular docking results of norisocorydine to (a) 3A4A receptor and (b) 3TOP receptor. In the plot the positions and orientations of acarbose which served as a comparison and native ligand of 3TOP receptor, as well as those of glucose, the native ligand of 3A4A receptor, are also shown. It can be seen that norisocorydine and acarbose occupied the same binding sites, both for 3A4A and 3TOP receptors. The predicted active compound also occupied the same binding sites as the native ligands for both 3A4A and 3TOP receptors.
